# Deep visual multi-omics profiling links morphology and molecular programs in clear cell renal cell carcinoma

**DOI:** 10.1038/s44321-026-00484-8

**Published:** 2026-08-06

**Authors:** Hella A Bolck, Ede Migh, Andras Kriston, Natalia Zajac, Susanne Kreutzer, Tiberiu Totu, Peter Leary, Ferenc Kovacs, Dorothea Rutishauser, Sibylle Pfammatter, Jonas Grossmann, Harini Lakshminarayanan, Debleena Basu, Cassandra Litchfield, Marija Buljan, Niels J Rupp, Peter Horvath, Holger Moch

**Affiliations:** 1 Department of Pathology and Molecular Pathology, University Hospital Zürich and University of Zürich, Zürich, Switzerland; 2 Centre for Artificial Intelligence, Zürich University of Applied Sciences, Winterthur, Switzerland; 3 Synthetic and Systems Biology Unit, Biological Research Centre, HUN-REN, Szeged, Hungary; 4 Single-Cell Technologies Ltd., Szeged, Hungary; 5 Functional Genomics Center Zürich, Eidgenössische Technische Hochschule Zürich/University of Zürich, Zürich, Switzerland; 6 Swiss Institute of Bioinformatics, Lausanne, Switzerland; 7 Nanomaterials in Health Laboratory, Swiss Federal Laboratories for Materials Science and Technology, St. Gallen, Switzerland; 8 Department of Health Sciences and Technology, Eidgenössische Technische Hochschule Zürich, Zürich, Switzerland; 9 Institute of AI for Health, Helmholtz Zentrum München, Neuherberg, Germany

**Keywords:** Cancer, Chromatin, Transcription & Genomics, Computational Biology

## Abstract

Clear cell renal cell carcinoma exhibits striking intra-tumoral heterogeneity at morphological and genetic levels, complicating treatment and contributing to disease progression. CcRCCs with rhabdoid differentiation are highly aggressive tumors characterized by distinct histopathologies. However, the relationship between morphology, underlying molecular alterations, and tumor behavior remains largely unclear. Here, we present Deep Visual Multi-Omics, an approach integrating digital pathology, morphology-guided single-cell isolation, and ultra-sensitive multi-omics profiling to link cell morphologies to their molecular underpinnings. Across five tumors, we profiled ~40,000 AI-classified and expert-curated cells. We identified progressive molecular dysregulation across cells with increasing histopathological grade coexisting within heterogeneous tumors as well as distinct molecular alterations associated with aggressive rhabdoid ccRCC cells, including signatures consistent with enhanced FOXM1-driven proliferation, altered cell-matrix interactions, and a putative immunomodulatory phenotype. Notably, rhabdoid cells exhibited elevated expression of IFN-beta, PD-L1, CD38, ITGB2, and integrin signaling, suggesting that they themselves may act as a source of signals influencing the local immune microenvironment. Besides providing new insights into the biology of ccRCC and highlighting avenues for future translational studies, this illustrates the potential of Deep Visual Multi-omics to dissect cancer heterogeneity and characterize high-risk cell populations.

## Synopsis

Intra-tumor heterogeneity drives tumor progression and therapy resistance. In ccRCC for example, distinct histopathologies coexist and are associated with patient outcome, yet their molecular basis is poorly understood. Deep Visual Multi-Omics links cell morphology to the underlying molecular state.

- Deep Visual Multi-Omics integrates digital pathology, morphology-guided single-cell isolation, and ultra-sensitive transcriptomic and proteomic profiling to characterize defined tumor cell populations from FFPE tissue.
- Morphology-resolved profiling of more than 40,000 individually selected cells revealed progressive molecular dysregulation across low-grade, high-grade, and rhabdoid cell populations coexisting within heterogeneous ccRCC tumors.
- Grade 4/rhabdoid ccRCC cells, a highly aggressive subpopulation, exhibited a distinct molecular profile characterized by FOXM1-driven proliferation and signals associated with extracellular matrix remodeling.
- In addition, rhabdoid ccRCC cell-intrinsic expression of factors like PD-L1, CD38, and ITGB2 implicated these cells as potential contributors to the local immune microenvironment.
- Deep Visual Multi-Omics provides a framework to link histopathological variation to molecular alterations, enabling identification of vulnerabilities in clinically relevant cell populations across many human cancers.

## Abstract (web summary)

Intra-tumor heterogeneity drives tumor progression and therapy resistance. In ccRCC for example, distinct histopathologies coexist and are associated with patient outcome, yet their molecular basis is poorly understood. Deep Visual Multi-Omics links cell morphology to the underlying molecular state.

## Graphical abstract

**Figure:**
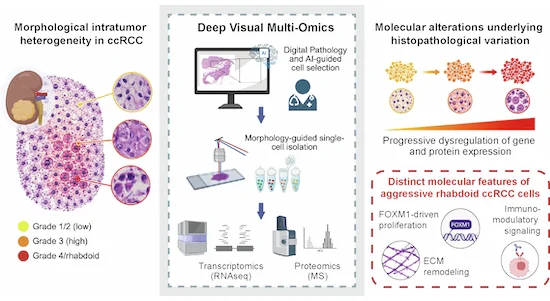

**Figure:**
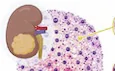

## Introduction

Clear cell renal cell carcinoma (ccRCC) is the most common subtype of renal cell carcinoma (RCC), accounting for the majority of metastatic cases and RCC-related deaths (Hsieh et al, 2017; Monda et al, 2023; Moch et al, 2022). The hallmark genomic alteration in ccRCC is loss of chromosome 3p concurrent with biallelic inactivation of the *VHL* tumor suppressor gene. *VHL* loss drives constitutive activation of hypoxia-inducible pathways and profoundly alters cellular programs controlling angiogenesis, metabolism, and survival (Hakimi et al, 2016). These early driver events also fuel extensive clonal evolution and genetic intra-tumor heterogeneity (ITH), hallmarks of ccRCC that complicate both accurate diagnosis and effective therapy (Turajlic et al, 2018b; The Cancer Genome Atlas Research Network, 2013; Bolck et al, 2021).

Morphological grading remains a cornerstone of patient management in ccRCC. The WHO/ISUP four-tiered system stratifies tumors from low (grades 1/2) to high (grades 3/4) grade based primarily on nucleolar prominence (Delahunt et al, 2019; Moch et al, 2016). Grade 4 tumors are additionally defined by the presence of sarcomatoid or rhabdoid differentiation, histopathological features that predict the poorest outcomes (Moch et al, 2016). Yet ccRCCs often display a mosaic of low- and high-grade phenotypes (Li et al, 2023b; Lopez et al, 2013), highlighting striking histo-morphological ITH and cell–cell variation (Gulati et al, 2014) and underscoring the limitations of bulk tissue profiling in capturing spatially distinct cell states.

Recently, sarcomatoid RCCs have received growing attention (Tannir et al, 2021; Bakouny et al, 2021) as they have shown notable responses to immune checkpoint inhibitor (ICI) treatments (Hahn et al, 2022; Wang et al, 2017; Malouf et al, 2020; Bakouny et al, 2021). This is potentially linked to their increased density of tumor-infiltrating lymphocytes (TILs) (Şenbabaoğlu et al, 2016; Hsieh et al, 2017). Much less is known about the molecular landscape and treatment outcomes of ccRCC with rhabdoid de-differentiation (Tannir et al, 2021), which are observed in 3–7% of RCC cases (Przybycin et al, 2014; Kara et al, 2016; Gökden et al, 2000). The available post hoc data suggest that these may also benefit from ICI treatment (Chahoud et al, 2020), but the mechanisms behind their putative ICI response represent a critical gap in our understanding. Unraveling the molecular drivers of rhabdoid morphology and their functional impact on tumor–immune interactions may provide new biological insights into these aggressive tumors.

Advances in artificial intelligence (AI)-based digital pathology, single-cell RNA sequencing (RNAseq), and ultra-sensitive proteomics have created new opportunities to dissect tumor heterogeneity at cellular resolution in situ (Brunner et al, 2022; Kashima et al, 2020; Marx, 2021; Baxi et al, 2022). AI enables reproducible, high-throughput classification of cellular phenotypes, while multi-omics molecular analysis can reveal the transcriptional and proteomic states of individually selected cells. However, these powerful technologies have not yet been combined into a single framework to link cellular morphology directly with the molecular programs to advance our understanding of cell-to-cell variation in human tumors (Brunner et al, 2022).

Here, we present Deep Visual Multi-Omics, a transformative workflow that bridges AI-guided histological cell classification, single-cell laser-capture microdissection, and ultra-sensitive multi-omics data collection from FFPE tissue samples. The strength of this study lies in morphology-resolved profiling of ~40,000 carefully selected patient from clinical ccRCC samples, where we map histopathological phenotypes to their underlying gene and protein expression landscapes, providing biological insights into molecular programs that drive cellular differentiation and define morphological ITH. In doing so, we generate new hypotheses regarding the distinct molecular features of rhabdoid ccRCC cells and how they may influence the clinical behavior of these aggressive tumors.

## Results

### Genome-wide genetic profiling of five ccRCCs with pronounced histo-morphological intra-tumor heterogeneity (ITH)

**Figure 1:**
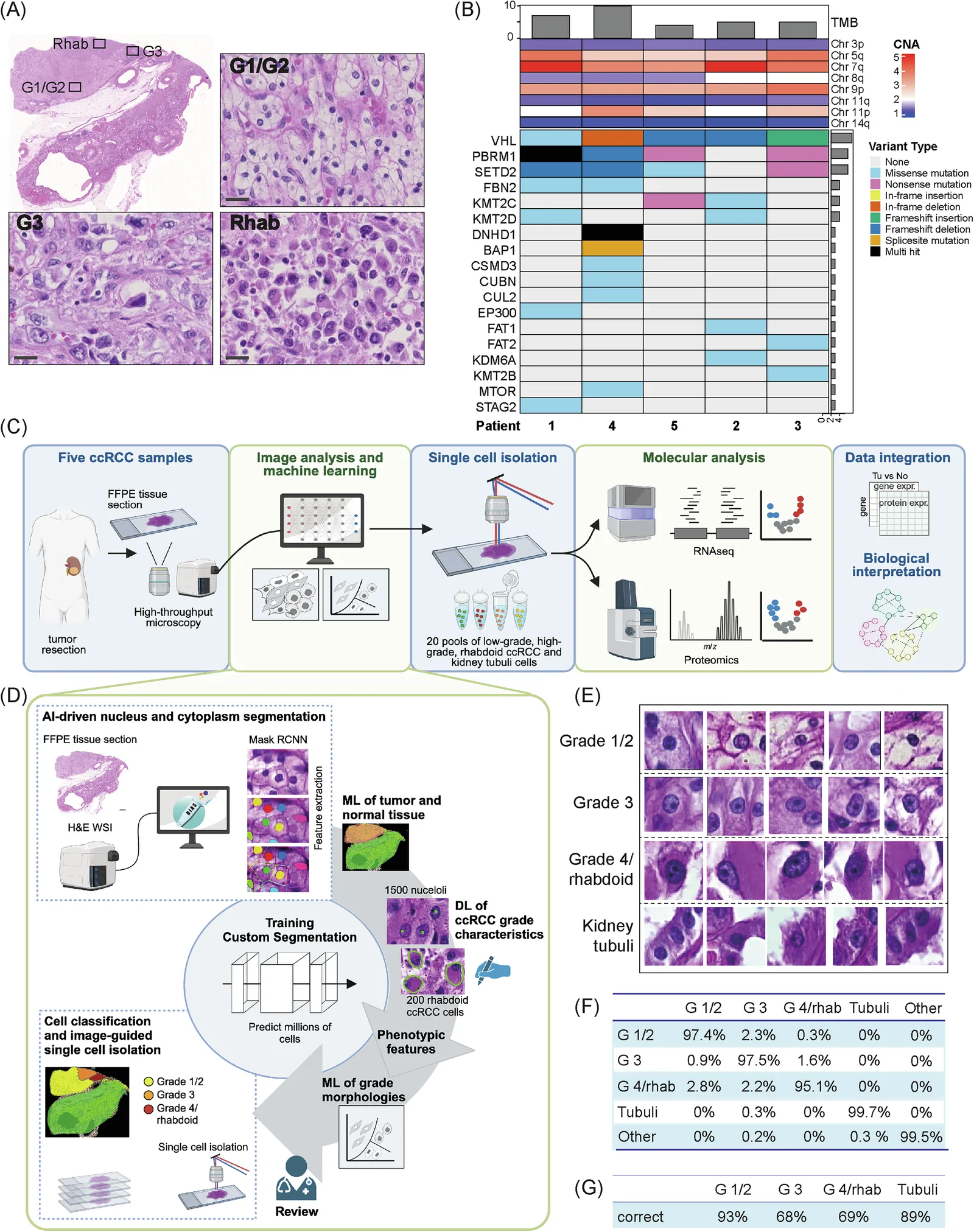
AI-driven, image-guided single-cell isolation and multi-omics analysis of ccRCC with pronounced morphological intra-tumor heterogeneity, including rhabdoid de-differentiation. (**A**) Representative H&E images (Patient 4) indicating ITH of grade features: Grade 1/2 (low-grade, G1/2), grade 3 (high-grade, G3), and grade 4 ccRCC with rhabdoid differentiation (Rhab). Scale bars: WSI—2 mm, high magnification panels—100 μm. (**B**) WGS was performed on all macrodissected tumor or normal regions per case (pooled), providing an overview of shared somatic alterations at the patient level, including TMB, selected copy number events, and driver mutations. (**C**) Overview of workflow combining AI-guided single-cell histological classification and isolation with transcriptomic and proteomic analysis. (**D**) AI-driven segmentation workflow for grade 1/2 (low), grade 3 (high), and grade 4/rhabdoid ccRCC as well as normal-appearing kidney tubuli cells in diagnostic tissue image using BIAS. (**E**) Example images of cells from the classes stated above identified by ML. (**F**) Cross-validation accuracies of our image analysis workflow using a randomized tenfold validation test. “Other” denotes artifacts as well as immune and stromal cells. Class-wise sensitivity (recall)/specificity values: G1/2: 97.4%/99.3%, G3: 97.5%/98.9%, G4/rhab: 95.1%/99.6%, Normal tubuli: 99.7%/99.9%. (**G**) Validation using independent unseen data (*N* = 6).

**Figure EV1:**
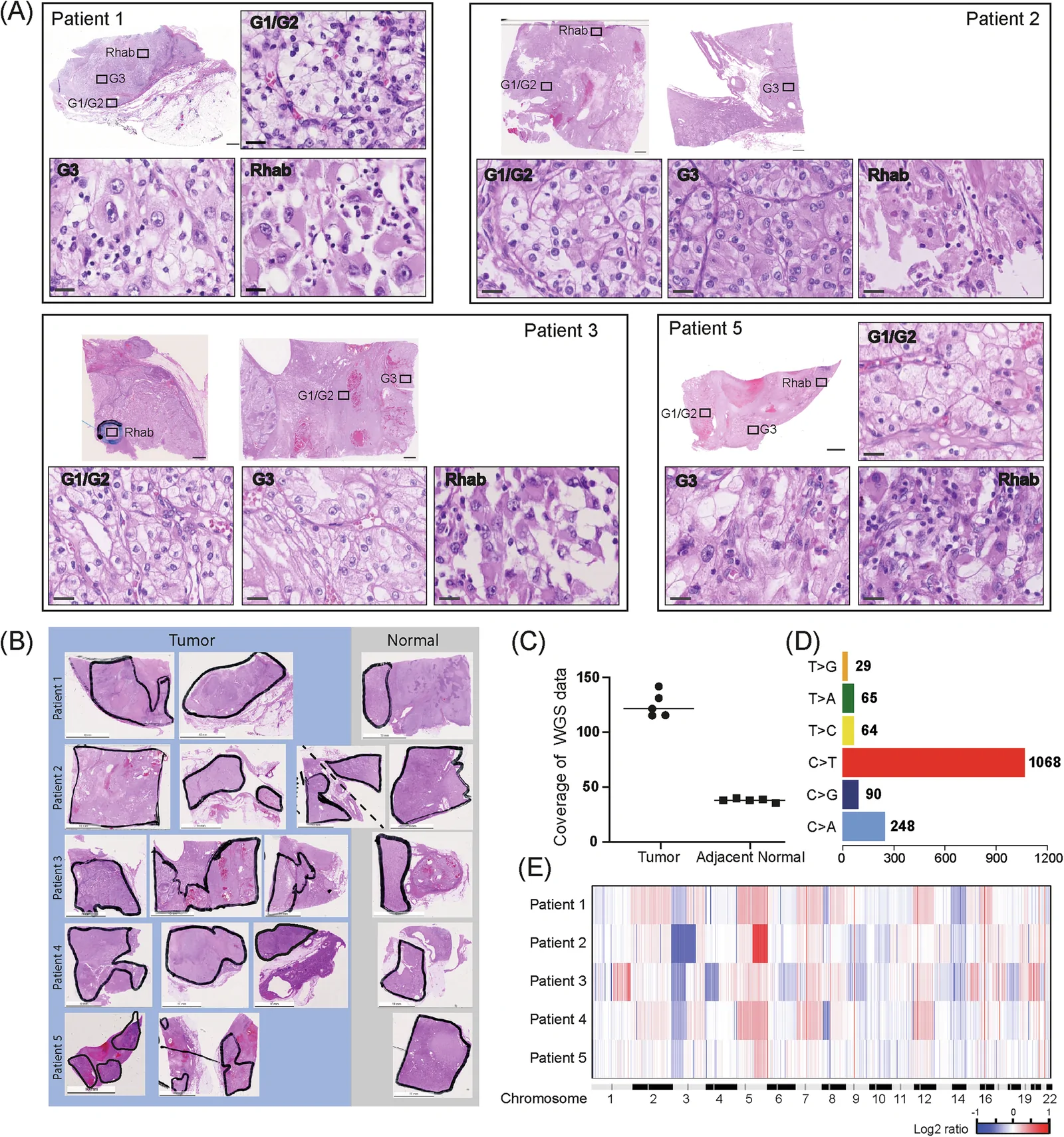
Image-guided single-cell isolation and multi-omics analysis of ccRCC with pronounced morphological heterogeneity and rhabdoid de-differentiation. (**A**) Representative H&E images indicating ITH of grade features: Grade 1/2 (low-grade, G1/2), grade 3 (high-grade, G3), and grade 4 ccRCC with rhabdoid differentiation (Rhab). Scale bars: WSI—2 mm, high magnification panels—100 μm. (**B**) Overview WSIs indicating the areas selected for dissection and subsequent WGS. The displayed sections were specifically prepared for region annotation on slides directly adjacent to those used for tissue dissection and WGS and are therefore distinct from the representative H&E sections shown in (**A**) and Fig. 1A. (**C**) Mean coverage of ccRCC and adjacent normal kidney in WGS data for all five cases. (**D**) Whole-genome mutation spectra indicating SNV classes, and their frequency, in our data, highlighting the presence of C > T transitions caused by cytosine deamination from formalin fixation of the tissue samples. (**E**) CNV profiles determined from WGS data. CNV loss/gain is indicated by color coding, blue indicates loss and red indicates gain.

We selected five ccRCC cases exhibiting pronounced tumor-intrinsic micro-heterogeneity defined by different grade morphologies within a tumor (Figs. 1A and  EV1A; Table 1 and Dataset EV1). Each sample underwent expert pathological assessment, confirming the co-occurrence of grade 1/2 (low grade), grade 3 (high grade), and rhabdoid (grade 4) ccRCC areas.

**Table 1:** Clinical data for the core ccRCC patient cohort were used for image-based single-cell isolation and molecular analysis in this study.

Patient	Subtype	ISUP grade	Tumor stage	Age	Gender	Progression	OS (months)
1	ccRCC	4	3a	66	m	Yes	98
2	ccRCC	4	3a	74	m	No	n/a
3	ccRCC	4	3a	77	w	Yes	2
4	ccRCC	4	3a	81	m	Yes	11
5	ccRCC	4	3a	74	m	No	n/a

Subsequently, we macrodissected tumor regions encompassing all morphological components (grades 1/2, 3, and 4) included in the image analysis and cell isolation workflow and pooled these into a single sample for DNA isolation per patient. These samples were subjected to whole-genome DNA sequencing (WGS) to capture the mutational background of each ccRCC at the case level (Fig. EV1C,D). Our analysis revealed that the tumor mutational burden (TMB) remained consistent across cases, aligning with previously reported values in ccRCC with and without rhabdoid features (The Cancer Genome Atlas Research Network, 2013; Bakouny et al, 2021) (Fig. 1B). Arm-level chromosomal aberrations included the most prevalent structural alterations found in ccRCC, 3p loss and 5q gain (Figs. 1B and  EV1E) (Mitchell et al, 2018). In addition, we consistently identified loss of chromosome 14q, a copy number variation (CNV) that has been associated with aggressive disease (Shen et al, 2011; Turajlic et al, 2018a). Our findings also include previously reported CNVs, such as gains in chromosomes 7q and 9p, as well as loss of chromosome 8q (Joseph C. Presti et al, 2002; Gupta et al, 2019; Turajlic et al, 2018a). Notably, all ccRCC samples exhibited mutations in the *VHL* tumor suppressor gene (*N* = 5) and harbored additional mutations in SETD2 (*N* = 4), PBRM1 (*N* = 4), BAP1 (*N* = 2) as well as other frequently mutated ccRCC drivers (Fig. 1B; Dataset EV1) (Turajlic et al, 2018a; The Cancer Genome Atlas Research Network, 2013). Thus, our genomic characterization largely corroborated findings from prior studies, providing confirmation of the typical molecular profiles of ccRCC samples.

### A deep digital pathology workflow quantified morphological heterogeneity in diagnostic images

For selection and isolation of the cells of interest (grade 1/2 (low-grade) ccRCC, grade 3 (high-grade) ccRCC, (grade 4/rhabdoid ccRCC, kidney normal tubuli) on HE-stained diagnostic whole-slide images (WSIs) with pronounced morphological heterogeneity (Fig. 1C,D), we used the BIAS software suite (Mund et al, 2022a). The AI workflow included training a deep neural network based on the Mask-RCCN architecture to segment nuclei across each WSI. To confirm the robustness of this segmentation backbone, we compared the resulting model “nucleAIzer” to several state-of-the-art models on publicly available WSI of 14 tissue types from The Cancer Genome Atlas (TCGA) evaluating 45,139 nuclei from randomly sampled regions. Across these, nucleAIzer showed competitive performance (Dataset EV1), supporting its suitability for nucleus segmentation in heterogeneous histopathology data. Although the original publication reported 4× scaling as optimal, performance at 3× scaling remained comparable in this study. As part of the ML module, we then extracted morphological and contextual features from nuclei, cytoplasm and surrounding cellular regions (Hollandi et al, 2020). Based on these, a pre-classification step, distinguished healthy kidney from tumor regions by aggregating features from the 10 closest neighbors for each individual cell (Toth et al, 2018). We additionally introduced a “junk/other” class to capture objects resulting from sample preparation, staining artifacts, or segmentation errors but also immune and stromal cells. This approach predominantly improved the differentiation between healthy and low-grade tumor cells during the downstream analysis. To enable grading, we trained a Mask-R convolutional neural network (R-CNN) using manual annotations of >1200 nucleoli typical for either grade 1/2 or grade 3/4 cells. In parallel, we developed a dedicated model for identifying rhabdoid differentiation (grade 4) by fine-tuning a Mask R-CNN using annotations from >250 rhabdoid cells derived from core cohort cases and additional samples (Dataset EV1). We iteratively expanded the training sets until the models reached stable performance within the annotated datasets, typically requiring 200–400 cells per class. We incorporated outputs from these segmentation models as additional features to train a classifier distinguishing (i) grade 1/2 (low-grade), (ii) grade 3 (high-grade), and (iii) grade 4 (rhabdoid) ccRCC from normal kidney tubuli cells (Fig. 1D,E). We assessed the performance of this cellular classification using tenfold cross-validation (Fig. 1F). For grading, it achieved high class-wise sensitivity (95–99%) and specificity (> 98%) within the annotated training domain (Fig. 1F), indicating that the feature-based approach captured morphological differences reliably. In addition, we evaluated performance on independent, unseen cases (Fig. 1G). Here, classification accuracy remained high for normal tubuli (89%) and grade 1/2 cells (93%), while performance decreased for grade 3 (68%) and grade 4/rhabdoid (69%), consistent with the known morphological continuum and overlap between these phenotypes.

For downstream multi-omics analysis, we used this AI system as a tool to prioritize candidate cells. We ranked predictions by confidence and manually reviewed top-scoring candidates prior to laser microdissection to exclude misclassified cells. We performed cell selection and laser microdissection on the same HE-stained sections, avoiding the need for registration to adjacent sections. We then selected two sets of 1200 cells for each morphologically defined class for each of the five ccRCC cases (Fig. 1C). For isolation, the nuclear contours were extended by 6 µm to define the cutting boundary and laser-captured these cells for downstream profiling.

### mRNA and protein-level alterations in ccRCC cells with morphologic heterogeneity reflect core disease genes and pathways

**Figure 2:**
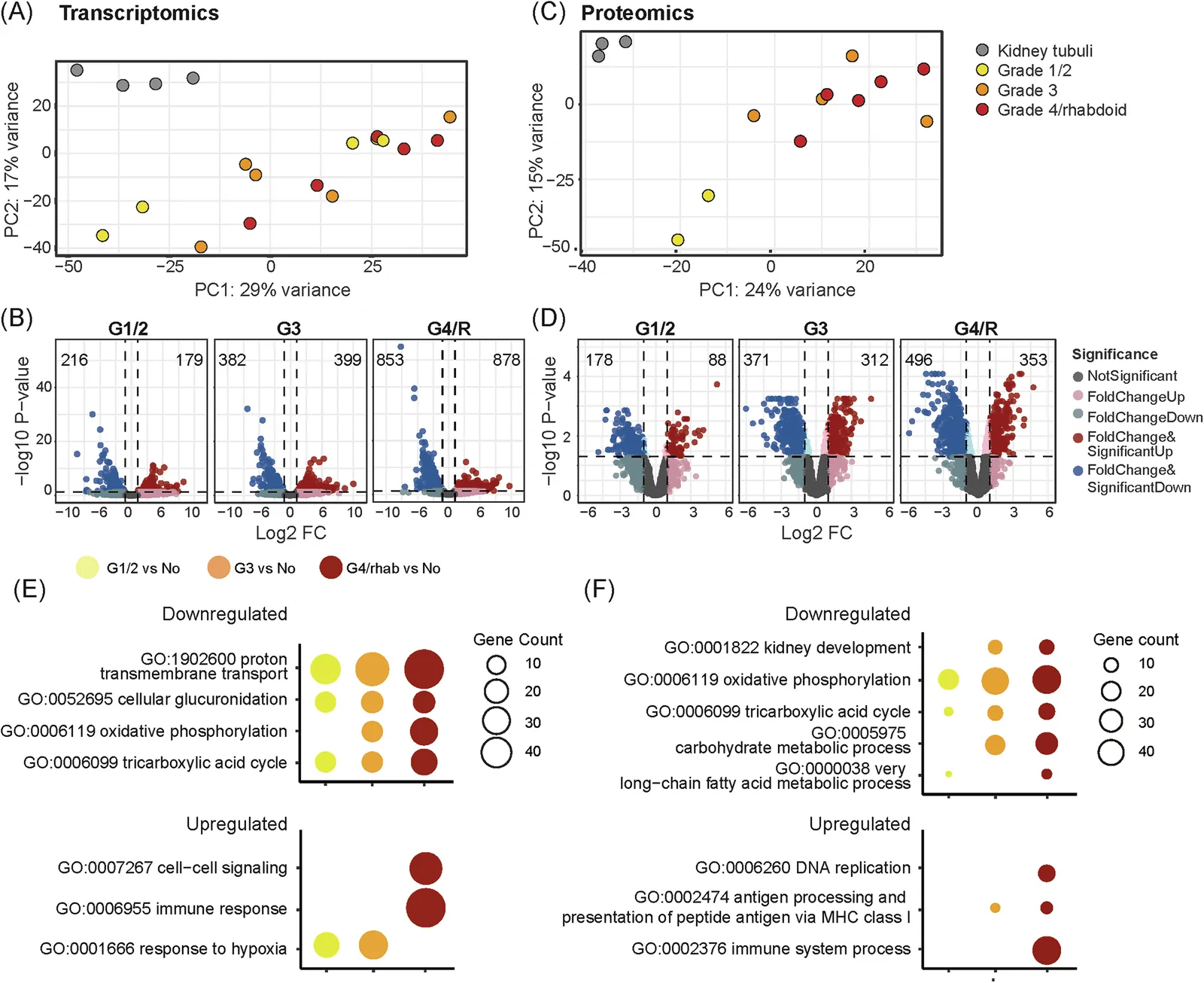
Differential expression analysis of mRNAs and proteins from individually isolated cells with distinct grade morphologies. (**A**) PCA of RNAseq data for 20,528 genes from five patients. (**B**) Volcano plot showing DEGs (DESeq2 negative binomial generalized linear model; Wald test, FDR < 0.05, logFC > |1 | ) in ccRCC grade groups compared to normal tissues. Analysis was performed between normal renal tubuli (*n* = 4 patients) and ccRCC grade groups (*n* = 5 patients per group). Genes that were significantly down- or upregulated are presented with red/blue-filled scatters and their number is indicated in the upper left and right corners, respectively. (**C**) PCA of LC-MS data for 4,382 proteins from five patients. (**D**) Volcano plot showing DEPs (moderated linear model implemented in prolfqua; Benjamini–Hochberg-adjusted FDR < 0.05, logFC > |1 | ) in ccRCC grade groups compared to normal tissues. Only proteomics samples passing quality control were included, and analysis was performed between normal renal tubuli (*n* = 3 patients) and ccRCC grade groups (*n* = 2 patients for grade 1/2, *n* = 4 patients for grade 3 and *n* = 5 patients for grade 4/rhabdoid). Proteins that were significantly downregulated or upregulated are presented with red/blue-filled scatters and their number is indicated in the upper left and right corners, respectively. (**E**) Enrichment analysis of associated GO biological pathways generated from over-representation analysis (hypergeometric test) for the transcriptomic datasets, indicating adjusted *P* values. (**F**) Same as (**E**) for the proteomic dataset. (**F**)

**Figure EV2:**
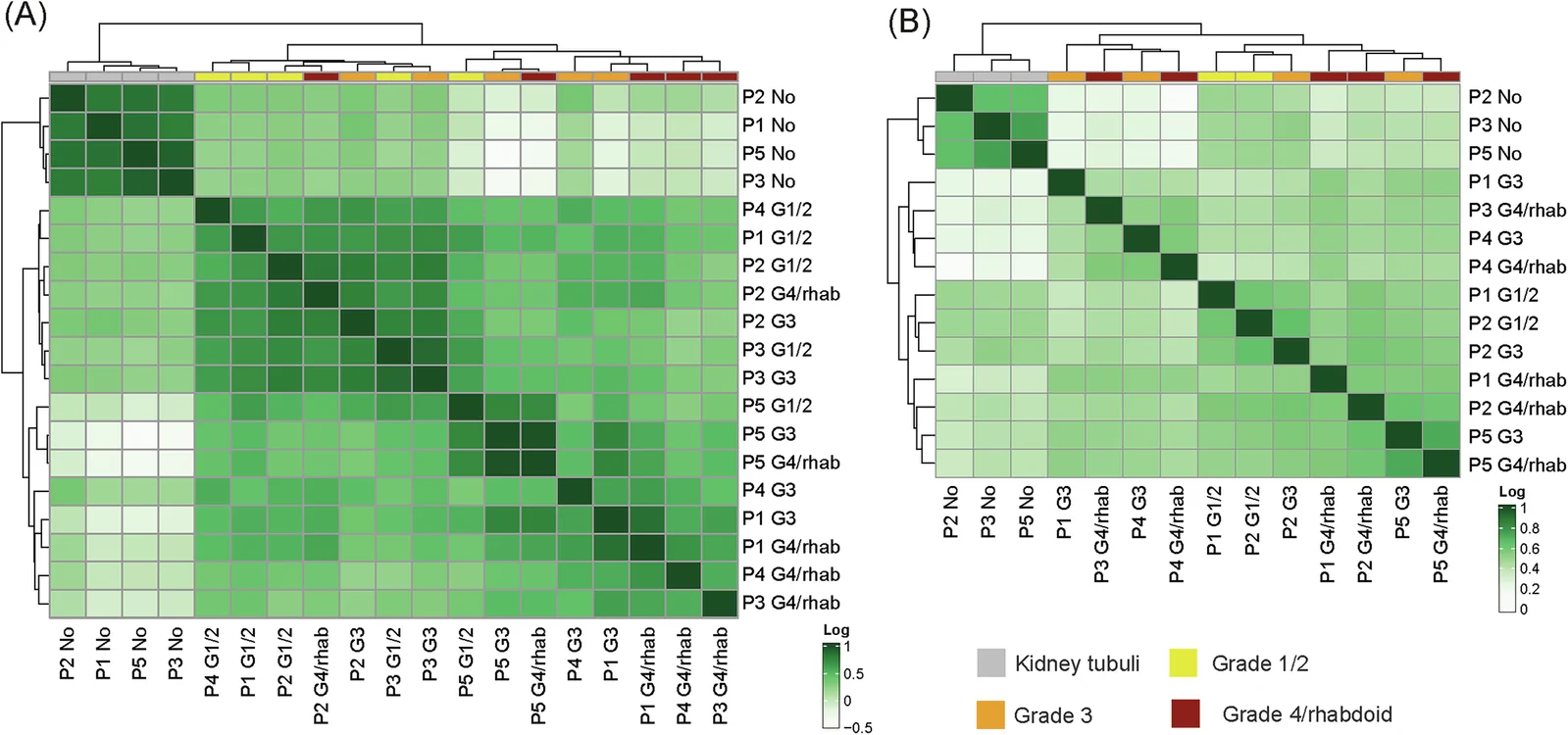
Analysis of mRNA and protein expression from individually selected cells with different grade morphologies. (**A**) Correlation analysis for top100 variance features of the transcriptomic dataset obtained from interrogating cells isolated from normal kidney tubuli tissue or ccRCC with distinct grade morphologies (*N* = 19). Correlation coefficients are indicated by the color code. (**B**) Correlation analysis for proteomics samples isolated from normal kidney tubuli tissue or ccRCC with distinct grade morphologies (*N* = 14). Correlation coefficients are indicated by the color code.

Following cell isolation, one pool of cells per morphological class was subjected to RNAseq analysis employing a protocol tailored for ultra-low sample inputs, which directly utilizes total RNA for library preparation input and relies on random priming. As a result, we obtained RNA expression data that mapped to 20,528 genes (Dataset EV1). The normal tubuli cells for patient 4 displayed substantial tubular atrophy and interstitial fibrosis and were therefore excluded from further analysis. Principal component analysis (PCA) and hierarchical clustering analysis of the gene expression data revealed a clear distinction between the profiles of normal tubuli and ccRCC cells of various morphologies (Figs. 2A and  EV2A). The PCA distances between ccRCC histologies were notably lower aligning with the expected similarity between these samples. However, a discernible trend of separation emerged between low- and higher-grade morphologies, particularly along PC1. Importantly, across all five tumors we observed similar separation patterns, indicating that these differences were not driven by single cases. We compared each of the ccRCC grade groups to the normal cells to determine differentially expressed genes (DEGs). The analysis revealed a significant increase in the number of both downregulated and upregulated mRNAs with higher grades (FDR < 0.05 and |Log2FC | > 1) (Fig. 2B).

Next, we investigated proteome alterations in ccRCC cells with heterogeneous histology by subjecting the second pool of isolated cells to LC-MS, adapting a highly sensitive workflow compatible with ultra-low sample inputs (Brunner et al, 2022). We successfully identified 4382 proteins. Six samples were excluded from further analysis due to irregularities during laser microdissection (LMD) that resulted in lower protein counts and noticeable batch effects (Dataset EV1). These exclusions were technical in nature and not associated with a specific morphological group. PCA and hierarchical clustering analysis of the remaining proteomics data confirmed a distinct separation of the normal tubuli and all morphological classes of ccRCC cells (Figs. 2C and  EV2B). Notably, the gradual transition among the ccRCC samples with increasing grades, as expressed by PCA distances, was more pronounced than observed in the transcriptomic data, particularly between cells with low (Grade 1/2) and higher (Grade 3/4) grades (Fig. 2A,C). Differential protein expression (DEP) analysis was subsequently performed using the remaining high-quality samples in an unpaired format, revealing an increasing number of differentially expressed proteins (DEPs) that correlate with higher ccRCC grade. This aligned with the trend observed in the transcriptomic data (Fig. 2D).

Over-representation analysis of the respective datasets showed that downregulation of biological processes was more prominent than upregulation. Biological enrichment in DEG and DEP downregulated datasets uncovered terms mirroring known ccRCC-specific tumorigenic changes, including oxidative phosphorylation and tricarboxylic acid cycle (TCA). Similarly, pathways related to normal kidney function (kidney development, glucuronidation, proton transmembrane transport) were increasingly downregulated with higher ccRCC grades (Fig. 2E,F; Dataset EV1). In contrast, biological pathways enriched in the upregulated datasets included response to hypoxia, DNA replication, and cell–cell signaling. Notably, among the pathways upregulated specifically in grade 4/rhabdoid cells, we identified the immune response and related processes.

Taken together, our data established that AI-driven classification of ccRCC cells with distinct histological features, followed by their individual LMD from diagnostic formalin-fixed paraffin-embedded (FFPE) tissue, yielded suitable samples for deep molecular profiling. We show that biologically meaningful patterns were evident in our RNAseq and proteomics data. Although patient-specific variability was visible, samples showed clear proximity by morphological grade in both datasets, indicating that grade-associated molecular programs are detectable across patients.

### Functional overlap between mRNA and protein dysregulation reveals progressive tumorigenic changes associated with increasing tumor grades

**Figure 3:**
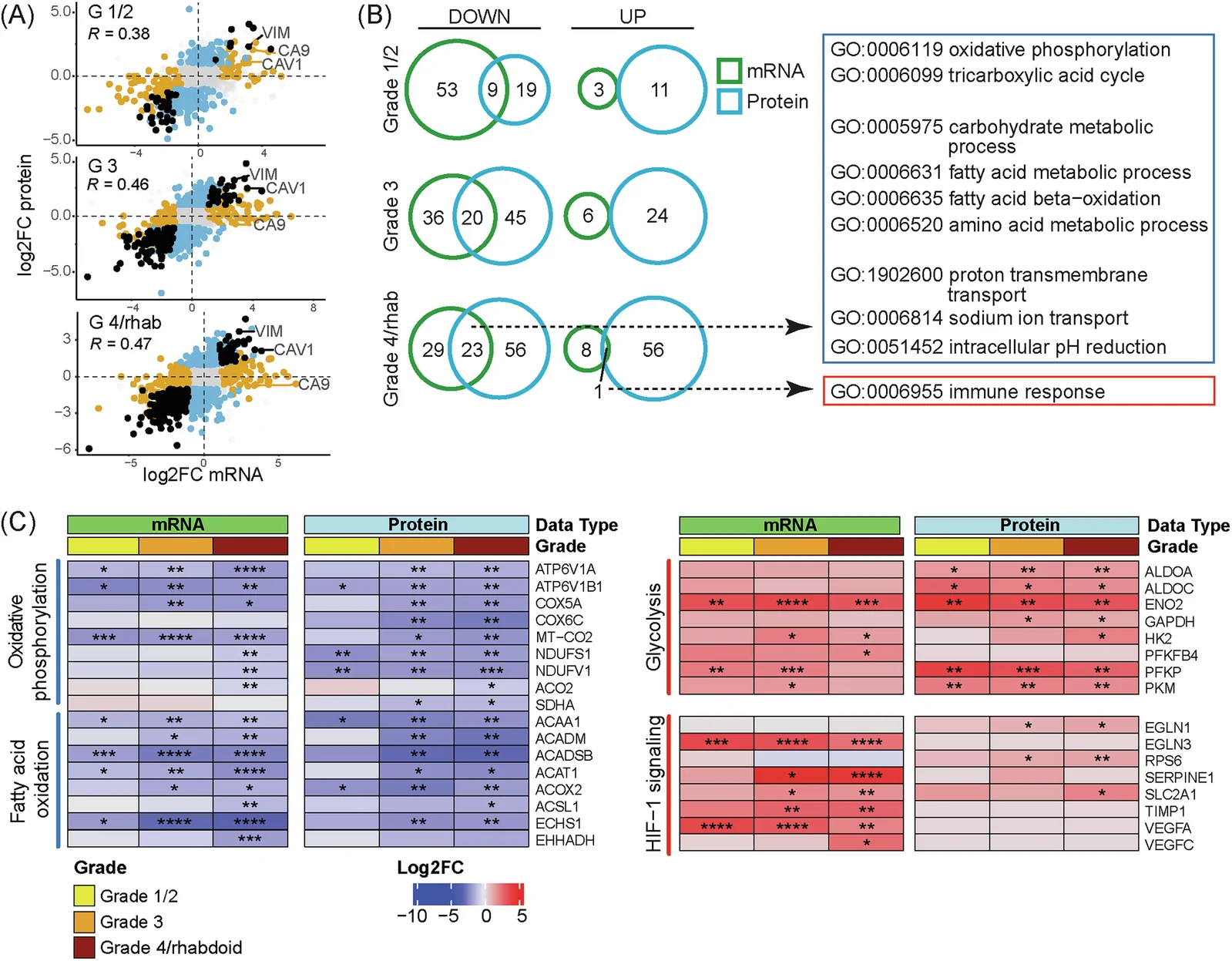
Functional overlap between mRNA and protein data. (**A**) Scatterplot of differential mRNA and protein expression between ccRCC grades and normal cells (Log2FC), indicating the correlation between datasets (R—correlation coefficient). Each dot represents one gene. Negative values indicate a decrease and positive values an increase in expression between tumor and NAT. (**B**) Venn diagram showing the number of enriched GO biological pathways in DEGs and DEPs for each grade group and the overlap between both data layers. Selected shared GO terms for grade 4/rhabdoid class of ccRCC cells are specified (for a complete overview, refer to Fig. EV4B). (**C**) Heatmaps depicting metabolic rewiring and upregulation of HIF1-signaling during ccRCC de-differentiation are evident on both data layers. Depicted genes were selected from significantly enriched pathways identified by ORA in the respective datasets and represent statistically deregulated and biologically relevant candidates. Alterations are shown as log2FC with respect to normal cells. The diagram on the left side depicts downregulated factors (blue), while on the right side upregulated factors are shown (red). Genes were selected from Glycolysis (Reactome R-HSA-70171), Fatty acid metabolism (Reactome R-HSA-8978868), Oxidative phosphorylation (KEGG path:hsa00190), and HIF-1 signaling (KEGG path:hsa04066). FDR is indicated by”****“ < =  0.0001, “***“ < = 0.001, “**“< = 0.01, “*“ < = 0.05.

**Figure EV3:**
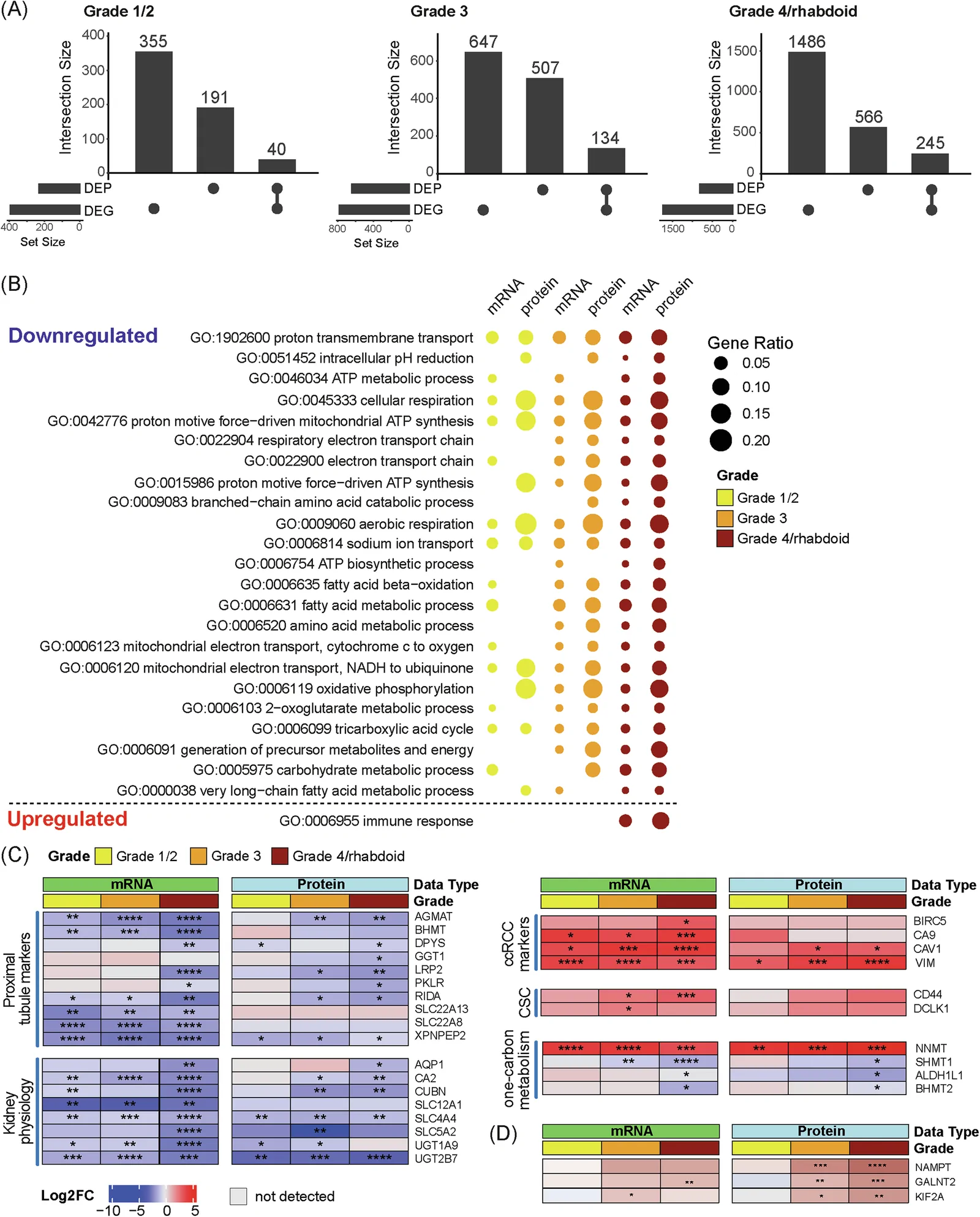
Functional overlap between mRNA and protein data. (**A**) Upset plots indicating the number of significant for mRNA and proteomics datasets for each grade group and their overlap. (**B**) Bubble plot depicting all 24 shared GO terms for mRNA and protein differential expression in the grade 4/rhabdoid class of ccRCC cells and their overlap with samples from other grade morphologies. (**C**) Heatmaps depicting selected alterations that mark kidney cell identity (proximal tubule cells markers and factors implicated in physiological kidney functions) as well as common ccRCC markers, cancer stem cell markers, and factors implicated in one-carbon metabolism. Representative genes and proteins are depicted as log2FC with respect to normal cells. FDR is indicated by “****“ <= 0.0001, “***“ < = 0.001, “**“< = 0.01, “*“ < = 0.05. (**D**) Heatmaps showing expression of KIF2A, NAMPT, and GALNT2 are consistent with previously reported signatures. Genes and proteins are depicted as log2FC with respect to normal cells. FDR is indicated by “****“ < = 0.0001, “***“ < = 0.001, “**“< =  0.01, “*“ < = 0.05.

We observed a notable correlation between mRNA and protein differential expression in our data (Fig. 3A). We detected 40, 134, and 245 significant genes shared across the mRNA and protein data for grade 1/2, grade 3, and grade 4/rhabdoid cells, respectively (Fig. EV3A). This degree of concordance is consistent with previous large-scale proteogenomic studies, which have shown that mRNA levels explain only a fraction of protein-level variation due to extensive post-transcriptional regulation, translation efficiency differences, and protein turnover mechanisms (Vogel and Marcotte, 2012; Liu et al, 2016). This relationship can be further modulated in cancer with extensive copy-number alterations (Fig. 1B) where transcript-level changes are buffered at the proteome level (Gonçalves et al, 2017; Di et al, 2025). Importantly, Vimentin (VIM) a key diagnostic marker was elevated in both datasets, while other ccRCC markers such as CA9 and CAV1, showed strong positivity in at least one of the -omics layers (Fig. 3A). Many regulated genes were distinct to one of the two data layers (Figs. 3A and  EV3A) prompting us to investigate whether these drive similar biological functions (Figs. 3B and  EV3B). Enrichment analyses uncovered widespread metabolic reprogramming in glucose and lipid metabolism as well as the repression of pathways related to physiological kidney functions in both datasets. Interestingly, often these alterations were observed across all ccRCC grades (Fig. EV3B). However, the number of overlapping biological pathways increased concomitantly with tumor grade (Fig. 3B), supporting the link between molecular dysregulation and higher-grade morphologies, aligning with known ccRCC pathobiology (Hakimi et al, 2016). The only upregulated biological pathway consistently shared across mRNA and protein datasets and specific to rhabdoid ccRCC cells was “immune response”, suggesting a potential contribution to an aggressive phenotype,

Consistent with the almost universal loss of VHL in ccRCC, upregulation of HIF-1 signaling was evident across all grade groups at both the mRNA and protein levels, and HIF1α transcriptional targets, such as VEGFA and GLUT1 (SLC2A1), showed significant increases in both -omics layers. (Fig. 3C). Concomitantly, HIF-signaling-mediated metabolic adaptations were evident from the data indicated by elevated expression of glycolytic enzymes across all ccRCC morphologies (e.g., ENO2, PFKB, PKM) and repression of oxidative phosphorylation, signified by downregulation of electron transport chain enzymes (NDUFS1, NDUFV1—Complex I; COX5A, COX6C – Complex IV; ATP6V1A, ATP6VB1—Complex V) and TCA cycle components (ACO2, SDHA) (Fig. 3C). Moreover, we observed significant alterations in lipid metabolism, with fatty acid beta-oxidation enzymes (ACAT1, ECHS1, EHHADH) being among the most significantly downregulated processes in both datasets. We also found evidence for progressive dysregulation of the one-carbon metabolism, a crucial pathway for tumor progression that has been recently described in relation to ccRCC aggressiveness (Fig. EV3C) (Qu et al, 2022). Reflecting the progressive loss of cellular identity during tumorigenesis and morphological transition towards higher-grade components, we observed a gradual downregulation of proximal renal tubule genes and enzymes related to physiological kidney functions, such as glucuronidation and transmembrane transport (Fig. EV3C). In parallel, grade 3 and grade 4/rhabdoid cells exhibited upregulation of stem cell markers such as CD44 and DCLK1 (Fig. EV3C), consistent with a de-differentiated cellular state (Weygant et al, 2015; Fendler et al, 2020).

Notably, support comes from a recent study (Li et al, 2023b) applying single-nucleus RNAseq to a rhabdoid ccRCC, which identified a distinct subcluster characterized by elevated expression of KIF2A, NAMPT, and GALNT2. In line with this, we found similar overexpression patterns in rhabdoid cells (Fig. EV3D). While not directly comparable, these observations support the ability of deep visual multi-omics to resolve morphology-associated molecular states.

### Integrated network analysis unveils cell–matrix interactions, and enhanced proliferation are key alterations characterizing rhabdoid ccRCC cells

**Figure 4:**
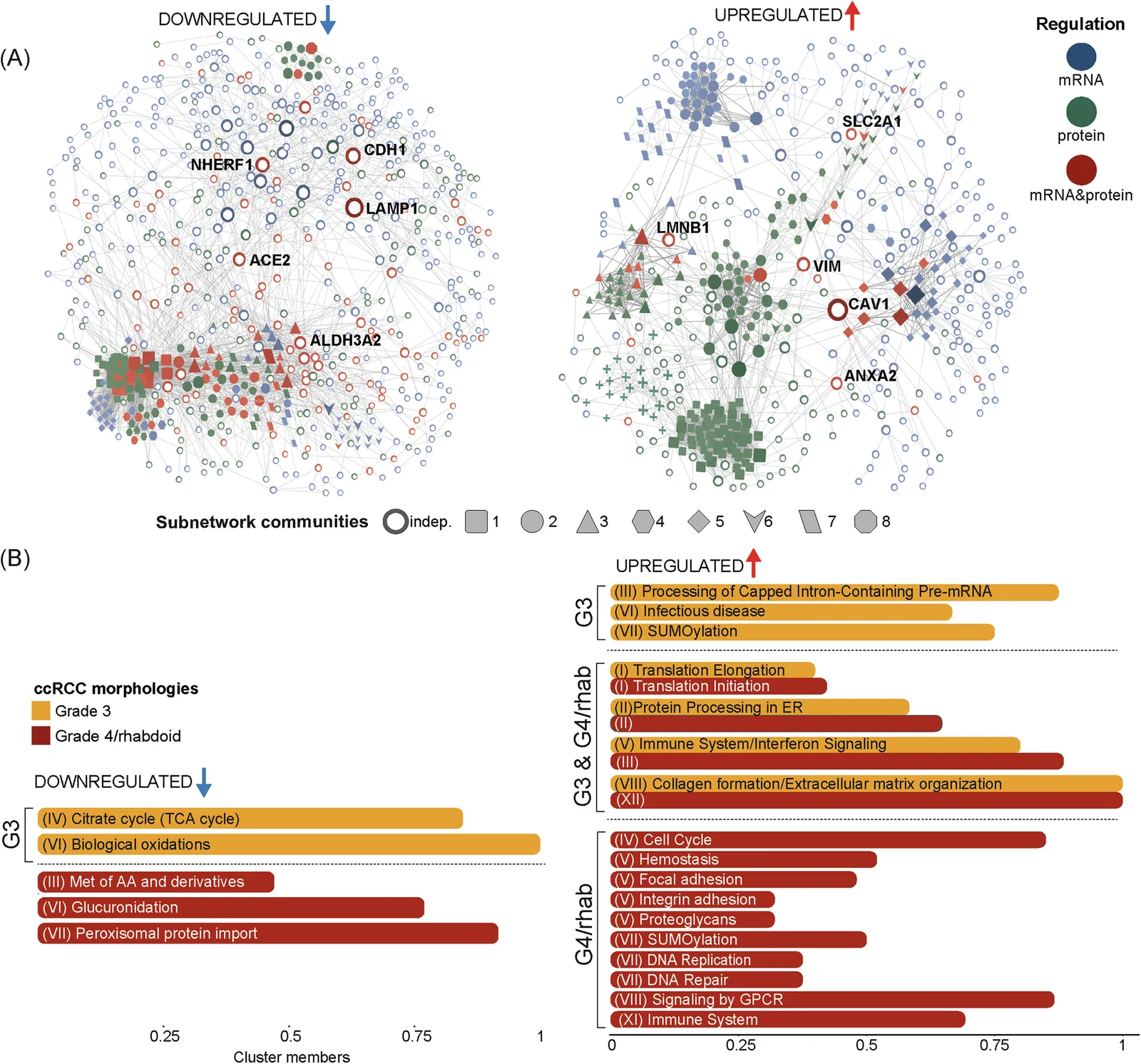
Multi-omics data integration uncovers distinct deregulated processes in aggressive high-grade and rhabdoid ccRCC cells. (**A**) PPI network distinct to G4/rhabdoid ccRCC cells built by integrating the significant elements from transcriptomic and proteomic datasets that were downregulated (*N* = 706) or upregulated (*N* = 501). Node sizes and darker colors correspond to higher centrality scores. The five top highly connected hub genes that are associated with no specific subnetwork are highlighted. Elements within network communities with defined biological functions, identified after the network decomposition, are specified. (**B**) Biological processes enriched in modules distinct to G3 and G4/rhabdoid cells with at least ten members following decomposition of the PPI networks in (**A**), based on KEGG and Reactome annotations. Enriched terms were determined by an FDR < 0.05 (Fisher’s exact test with Benjamini–Hochberg correction). Only non-redundant terms are presented, except where additional terms provided complementary information. The fraction of proteins in each module with the corresponding annotation is provided.

**Figure EV4:**
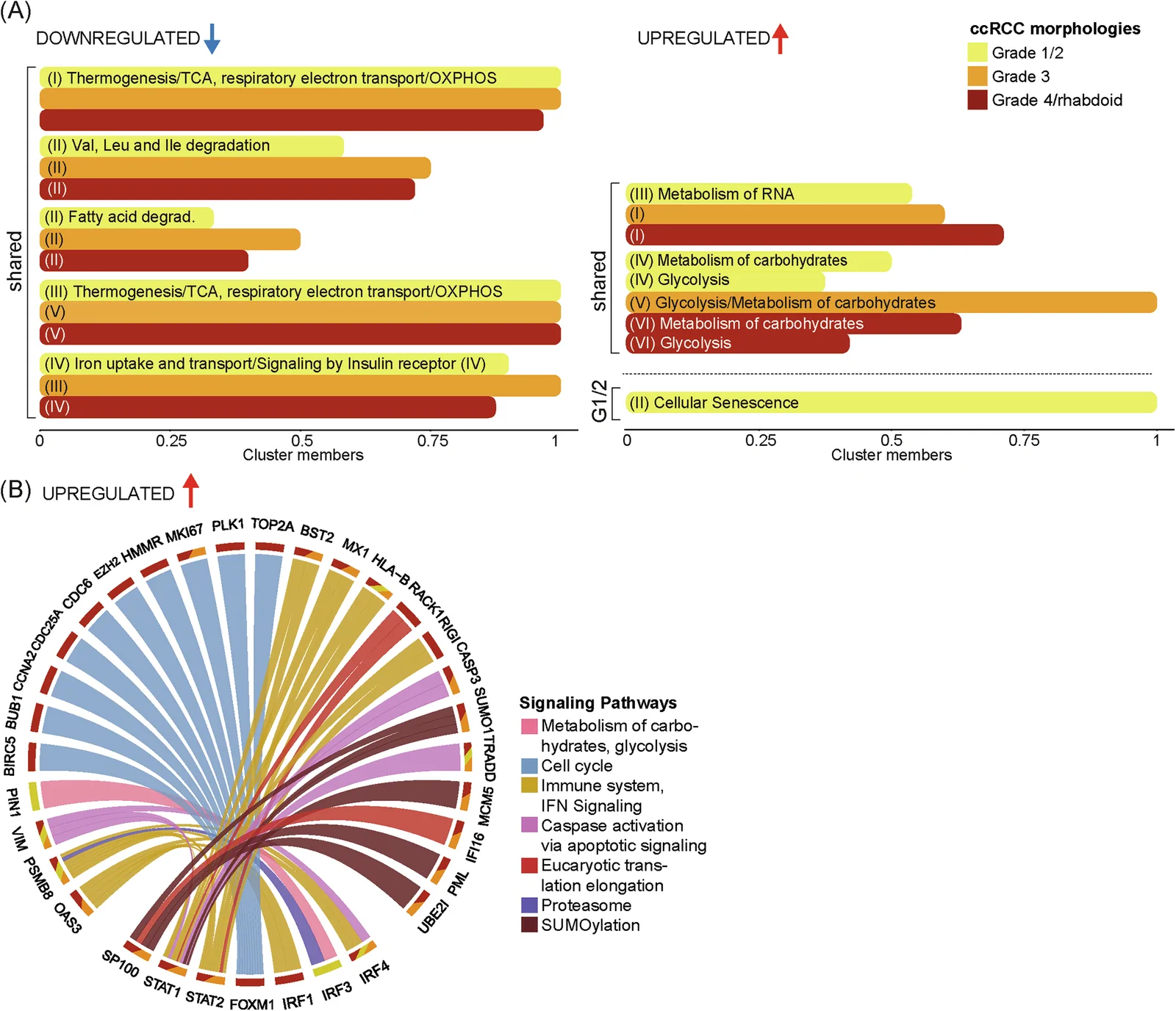
Multi-omics data integration uncovers distinct deregulated processes in aggressive cells and their transcriptional regulation. (**A**) Biological processes enriched in modules shared by Grade 1/2 ccRCC grade morphology with at least ten members following decomposition of the PPI networks in (**A**) based on KEGG and Reactome annotations. Enriched terms were determined by an FDR < 0.05 (Fisher’s exact test with Benjamini–Hochberg correction). Only non-redundant terms are presented, except where additional terms provided complementary information. The fraction of proteins in each module with the corresponding annotation is provided. (**B**) Circular diagram highlighting the observed interaction between upregulated TFs (bottom) and their targets (top) within the top 30% nodes of the PPI networks based on the current-flow betweenness centrality for the three cell morphologies analyzed individually. Tracks mark the grade groups in which they were found to be significantly dysregulated (corresponds to the color-code in (**A**)) and the bands color-code for the associated signaling pathways.

**Figure 5:**
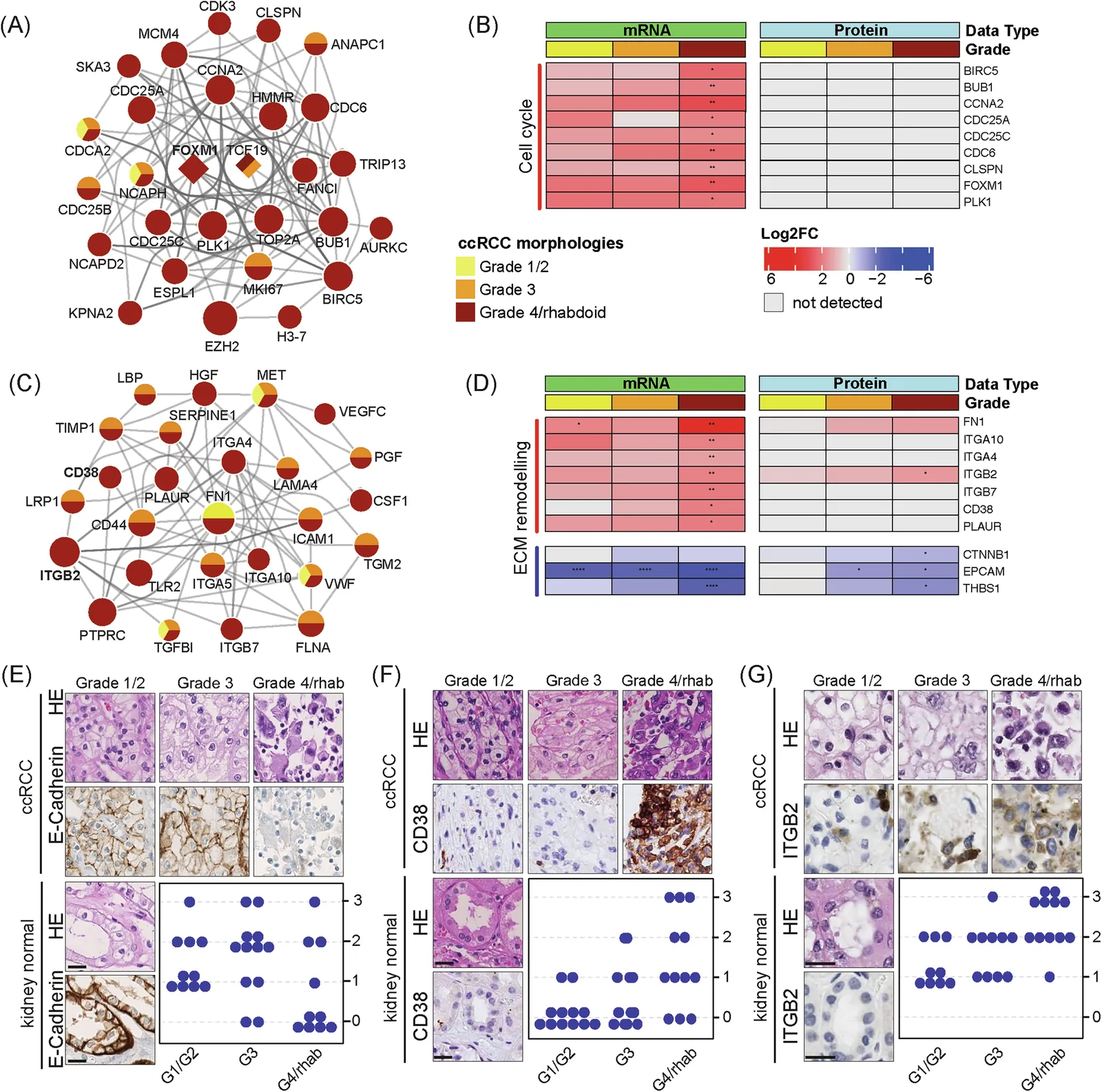
Grade 4/rhabdoid ccRCC cells are characterized by enhanced expression of cell cycle factors and dysregulation of cell–matrix interactions. (**A**) Network decomposition highlighting a subnetwork associated with upregulation of cell cycle factors (Reactome R-HSA-1640170) in the grade 4/rhabdoid ccRCC cells (Module IV). Factors that are significantly upregulated also in lower-grade ccRCC cells are indicated. Bigger nodes correspond to higher centrality values in the Grade 4/rhabdoid ccRCC PPI network. Grades are color-coded as in (**B**). (**B**) Heatmaps highlighting selected elements from module IV and their respective dysregulation during ccRCC de-differentiation on both data layers. Alterations of representative genes and proteins are depicted as log2FC with respect to normal cells. FDR is indicated by “****” <= 0.0001, “***” <= 0.001, “**” <= 0.01, “*” <= 0.05. (**C**) Network decomposition highlighting a subnetwork containing upregulated elements involved in focal adhesion (KEGG path:hsa04510), integrin cell surface interactions (Reactome R-HSA-216083), homeostasis (Reactome R-HSA-109582), and proteoglycans implicated in cancer (KEGG path:hsa05205) in the grade 4/rhabdoid ccRCC cells (Module V). Factors that are significantly upregulated in lower-grade ccRCC cells are indicated. Bigger nodes correspond to centrality values in the Grade 4/rhabdoid ccRCC PPI network. Color codes as in (**B**). (**D**) Heatmaps highlighting selected elements implicated in ECM remodeling and cell adhesion, and their respective dysregulation during ccRCC de-differentiation on both data layers. Alterations of representative genes and proteins are depicted as log2FC with respect to normal cells. Color codes are depicted in (**B**). FDR is indicated by”****“ <= 0.0001, “***“ <= 0.001, “**“<= 0.01, “*“ <= 0.05. (**E**) IHC analysis of E-cadherin (CDH1) expression ccRCC with distinct grade morphologies. Representative images are shown for each grade group, the scale bar represents 20 μm. Bottom right: Expression levels were scored from 0 (no expression) to 3 (high expression) and the quantification of *N* = 12 cases is shown (EV1). (**F**) Same as (**E**) for CD38. (**G**) Same as (**E**) for ITGB2.

**Figure EV5:**
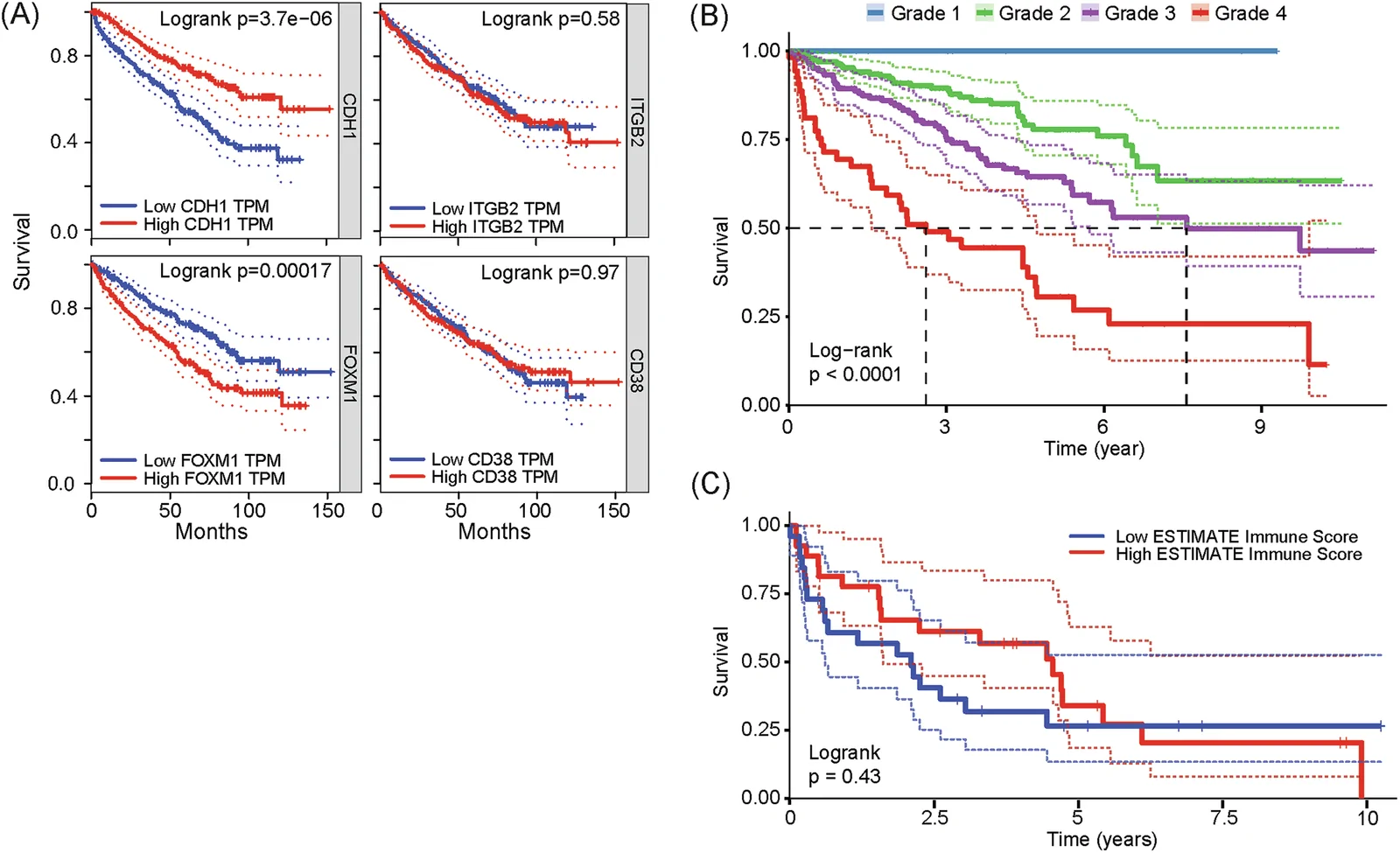
Survival associations of molecules and tumor grade in ccRCC. (**A**) Kaplan–Meier survival analysis in the TCGA-KIRC dataset (*N* = 516) showing overall survival. FOXM1 with HR (high) = 0.66 p(HR) = 0.0079. CDH1 with HR (high) = 0.48 p(HR) = 5.9e-06. CD38 with HR (high) = 1 p(HR) = 0.97. ITGB2 with HR (high) = 1.1 p(HR) = 0.58. (**B**) Kaplan–Meier survival curves stratified by tumor grade in the TCGA-KIRC cohort (*N* = 434). (**C**) Kaplan–Meier survival curves in Grade 4 ccRCC cases stratified by ESTIMATE immune score cohort (*N* = 53). HR (high) = 1 p(HR) = 0.64.

As the direct overlap between mRNA and protein expression was limited, we employed integrative network-based analysis of transcriptomic and proteomic profiles to assess whether dysregulation on both layers would point to similar processes, offering deeper insights into the molecular programs underlying ccRCC tumor progression and histological heterogeneity. Specifically, we combined differentially expressed elements for both data layers and constructed grade-specific networks based on knowledge-based high-confidence protein–protein interactions (PPI) networks. We used the current flow betweenness centrality metric in order to identify central nodes that connect elements in each network and further examined the top 30% most central elements (Totu et al, 2025) (Fig. 4A; Dataset EV2). Specifically, in the grade 4/rhabdoid-specific network, downregulated central nodes included factors implicated in renal functions (e.g., ACE2, NHERF1, ALDH3A2) as well as cell adhesion and EMT (e.g., CDH1). Conversely, in the network of upregulated factors, central elements reflected cellular adaptations related to processes such as EMT, cell migration, adhesion (e.g., VIM, ANXA2), as well as metabolic reprogramming consistent with the Warburg effect (e.g., SLC2A1). Together, these central network nodes, integrating mRNA and protein dysregulation, highlight perturbations in pathways known to be altered in ccRCC and support the reprogramming of cellular processes associated with a more aggressive and metastatic phenotype in grade 4/rhabdoid cells. We next applied a network modularization tool (MONET) to examine functionally related network modules that could describe distinct biological changes in ccRCC cells with different grade morphologies. In Grade 4/rhabdoid ccRCC cells, we discovered a number of unique modules with biological functions not shared by the other morphologies (Fig. 4B). Downregulated modules contained largely metabolic processes previously associated with ccRCC tumorigenesis, but pathways associated with upregulated elements provided important and novel insights into the biology of rhabdoid differentiation (Figs. 4B and  EV4A). For instance, Forkhead box transcription factor 1 (FOXM1) emerged as a prominent hub in the network constructed for cells with rhabdoid differentiation in Module IV (Fig. 5A). This node was of much lower significance in low- and high-grade ccRCC networks (Figs. 5A and  EV4A), indicating that FOXM1 may contribute to the elevated potential for proliferation in rhabdoid ccRCC cells) (Han et al, 2018). Consistently, we noted that FOXM1 expression was associated with poor prognosis in the TCGA-KIRC cohort (Fig. EV5A), supporting a potential relevance for patient outcomes. Additionally, the cell cycle subnetwork in rhabdoid cells encompassed several molecules that function downstream of the FOXM1 transcription factor, such as DNA replication factors (CLSPN, CDC6), elements implicated in the G1/S- (CDC25A, CCNA2, CDK3) and G2/M-checkpoints (PLK1, CDC25A/C, BUB1) (Figs. 5A,B and  EV4B). Their dysregulation could further contribute to the increased proliferative potential of rhabdoid ccRCC cells. Additionally, in this subnetwork, we observed rhabdoid ccRCC cell-specific upregulation of BIRC5 (Fig. 5B), a well-established inhibitor of apoptosis protein (IAP).

Module V in the G4/rhabdoid-specific network encompassed elements involved in focal adhesion, integrin cell surface interactions, homeostasis and proteoglycans implicated in cancer (Fig. 5C; Dataset EV2) thus suggesting extracellular matrix (ECM) remodeling processes are relevant for ccRCC progression to higher grades. Importantly, this module contained several genes that were specific for the grade 4/rhabdoid network with high centrality such as integrins (ITGA4, ITGA10, ITGB2, ITGB7) that have known roles in tumor microenvironment (TME) interactions, tumor growth, and metastatic dissemination (Arun et al, 2018). Other upregulated elements with known functions in modulating the TME were included in this module, including FN1, PLAUR, VEGFC, and CD38. Conversely, we observed downregulation of THBS1, CDH1, and EPCAM in the rhabdoid network (Fig. 5D; Dataset EV2), indicative of dysregulation of cell–matrix interactions and enhanced activation of EMT, processes known to be implicated in ccRCC progression. To provide orthogonal validation, we performed immunohistochemical (IHC) in an extended cohort of morphologically heterogeneous ccRCC cases staining for CD38, ITGB2, and E-cadherin (CDH1), confirming their expression patterns were consistent with our multi-omics findings (Dataset EV1; Fig. 5E–G). Furthermore, CDH1-mRNA expression associated with overall survival in the TCGA-KIRC cohort (Fig. EV5A) but not more strongly than histologic grade, which provided the most pronounced separation (Fig. EV5B), consistent with its dominant prognostic role. CD38 and ITGB2 displayed weaker or no clear separation.

These results provide insight into molecular programs associated with the malignant potential of rhabdoid ccRCC cells and highlight alterations in pathways related to cell adhesion and proliferation, which may relate to the observed morphological features, such as large cell size, discohesive growth patterns, and increased mitotic activity.

### Tumor-intrinsic immune-related programs in rhabdoid ccRCC cells

**Figure 6:**
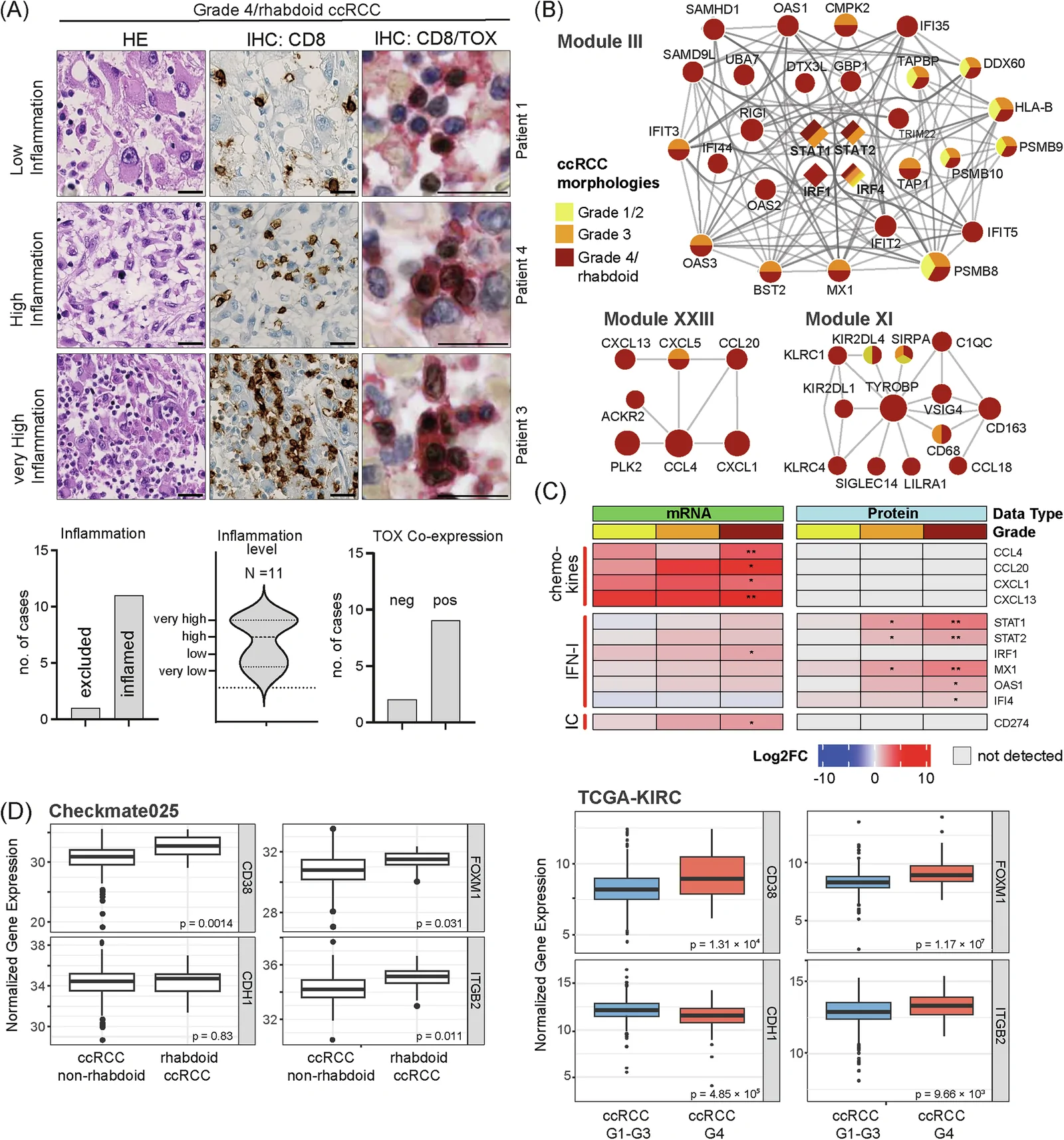
Aggressive ccRCC cells show upregulation of the immune response. (**A**) IHC analysis using antibodies against CD8 depicting infiltration of CD8^+^ T cells into the rhabodoid component of the ccRCC cases in this study (middle panel). CD8 + T cells were scored and assigned to the depicted immune phenotypes (inflamed or excluded) by pathologist review. Inflamed tumors were further categorized into low, high, or very high inflammation levels, and representative images for each phenotype are shown. The scale bar represents 20 μm (*N* = 12). Co-expression of TOX was assessed in CD8^+^ T cells as depicted in the right panel. The scale bar represents 20 μm (*N* = 12). The presence of TOX co-expression was recorded. (**B**) Integrated network analysis showed a significant enrichment of upregulation of immune response elements in Modules III (Interferone Signaling, Rectome R-HSA-913531 and Immune System, Reactome R-HSA-168256), XI (Immune System, Reactome R-HSA-168256), and XXIII (Chemokine receptors bind chemokines, R-HSA-380108) of the grade 4/rhabdoid ccRCC network. Factors that are significantly upregulated also in lower-grade ccRCC cells are indicated. Bigger nodes correspond to higher centrality values in the grade 4/rhabdoid ccRCC PPI network. (**C**) Heatmaps highlighting selected elements associated with the immune system-related pathways and their respective dysregulation during ccRCC de-differentiation on both data layers. Alterations of representative genes and proteins are depicted as log2FC with respect to normal cells. FDR is indicated by”****“ <= 0.0001, “***“ < = 0.001, “**“<= 0.01, “*“ < = 0.05. (**D**) Boxplots of normalized gene expression in non-rhabdoid vs. rhabdoid ccRCC patients from the CM-025 clinical trial cohort for the everolimus (Non-rhabdoid ccRCC *N* = 108, Rhabdoid ccRCC *N* = 6) as well as nivolumab (Non-rhabdoid ccRCC *N* = 139, Rhabdoid ccRCC *N* = 8) arms (left). Significance was tested using a Welch *t* test and *P* values are displayed. For the TCGA-KIRC cohort (right) grade 1–3 vs. grade 4 ccRCC cases were compared (*N* = 0.434). Significance was assessed using a two-sided Wilcoxon rank-sum test, and *P* values are displayed. All boxplots show the median (center line), the 25th and 75th percentiles (box boundaries), whiskers extending to 1.5× the interquartile range, and points representing outliers.

Our multi-omics analysis suggested that immune-related signatures were enriched in rhabdoid ccRCC cells (Figs. 2E,F and 3B). To further assess the immune TME, we evaluated T-cell infiltration in our extended cohort of grade 4/rhabdoid ccRCCs and detected CD8⁺ T-cell in all cases (Dataset EV1; Fig. 6A). Eleven cases displayed an inflamed phenotype, whereas one exhibited an immune-excluded pattern, with CD8⁺ T cells confined to the tumor border. At the molecular level, grade 4/rhabdoid ccRCC cells exhibited unique immune-related elements in upregulated Modules III, XI and XXIII (Fig. 6B). Specifically, we observed elevated expression of chemokines including CCL4, CCL20, CXCL1 and CXCL13 (Fig. 6C). In addition, we identified activation of type I interferon signaling, including increased STAT1/2 and enrichment of downstream interferon- beta-associated targets (Langbein et al, 2022) such as OAS1, MX1, and IFI44 among others (Figs. 6B,C and EV4C). These features were evident in both grade 3 and grade 4/rhabdoid cells. Moreover, we detected upregulation of the immune checkpoint ligand PD-L1 (CD274) in rhabdoid cells (Fig. 6C).

In the context of the immune response, CD38 and ITGB2, previously identified within the rhabdoid-specific ECM and cell adhesion network module (Fig. 5C,D), highlight a potential link between ECM remodeling and immune modulation. CD38 showed consistent upregulation in rhabdoid ccRCC cells and has been implicated in the modulation of T-cell activity Piedra-Quintero et al, 2020. In line with this, we additionally observed co-expression of the T-cell-exhaustion marker thymocyte selection-associated high mobility group box (TOX) in nine out of eleven inflamed cases with infiltrating CD8^+^ T cells (Dataset EV1; Fig. 6A), pointing to an exhausted T cell state in rhabdoid ccRCC. In contrast, ITGB2 is primarily involved in immune cell adhesion and signaling (Xu et al, 2022), implying that alterations in rhabdoid ccRCC ECM interactions may influence immune cell behavior.

We next assessed whether transcriptional programs identified in our morphology-resolved cohort were reflected in independent datasets. We first analyzed the CheckMate 025 cohort and observed concordant regulation of FOXM1, CD38, ITGB2, and CDH1 (E-Cadherin) in rhabdoid ccRCC across treatment arms (Fig. 6D). We then extended this analysis to the TCGA-KIRC cohort. As rhabdoid differentiation was not specifically annotated in this dataset, we analyzed all grade 4 tumors as a surrogate for aggressive ccRCCs. Likewise, we observed directionally consistent expression changes, including increased FOXM1, CD38, and ITGB2, and decreased CDH1 (E-Cadherin), supporting the association of these markers with aggressive disease phenotypes. To further examine the microenvironmental context, we analyzed the ESTIMATE immune score, which captures immune-cell admixture in bulk transcriptomic data (Yoshihara et al, 2013). Within Grade 4 tumors, higher immune scores showed a trend towards differences in overall survival; however, this did not reach statistical significance (Fig. EV5C), indicating that immune infiltration alone does not robustly stratify patient outcomes (Sobottka et al, 2024). We examined available treatment annotations in the CheckMate 025 and TCGA-KIRC cohorts and identified only a very small subset of Grade 4/rhabdoid cases with available expression data and recorded ICI exposure (*N* = 8 and *N* = 10, respectively), precluding any conclusions regarding treatment response.

Taken together, our findings point to a rhabdoid cell-specific contribution in altering the tumor immune microenvironment, characterized by activation of immune-modulatory factors that include established molecules like PD-L1 (CD274), as well as CD38 and ITGB2. Notably, these factors were expressed by rhabdoid ccRCC cells themselves, suggesting that they may directly participate in shaping their TME and could influence the activity of infiltrating CD8⁺ T cells.

## Discussion

Morphological ITH is a hallmark in ccRCC, yet the molecular drivers of histopathological variation have remained elusive (Li et al, 2023b; López, 2016; Singh et al, 2015). Molecular analyses specifically resolving the alterations underlying rhabdoid morphology in ccRCC remain scarce, with most available data coming from small pathology-driven cohorts (Gupta et al, 2019; Singh et al, 2015). Larger datasets either lack standardized rhabdoid annotation (The Cancer Genome Atlas Research Network, 2013), include only very small numbers of rhabdoid cases (Li et al, 2023b; Braun et al, 2020), or analyze sarcomatoid and rhabdoid RCCs in aggregate (Bakouny et al, 2021). By applying deep visual multi-omics to ccRCC tissue samples, our study provides morphology-resolved, multilayer molecular characterization of highly enriched, clinically relevant cell populations and offers new molecular insight into features associated with aggressive phenotypes. Our AI-driven automated classification system reliably categorizes ccRCC cell morphologies in HE-stained WSI. We demonstrated robust separation of the defined morphological classes (grade 1/2, grade 3, and grade 4/rhabdoid. For a minority of cells, misclassifications occurred between grade 3 and grade 4/rhabdoid cells, which form a recognized morphological continuum and can be difficult to distinguish even for experts. Such systems hold strong potential to enhance diagnostic accuracy and overcome limitations of manual annotation. In this work, our scalable approach also enabled the precise, morphology-guided selection of tumor and normal cells for downstream transcriptomic and proteomic analyses. While no image registration was required in our procedure, very minor contributions from surrounding stromal or immune cells cannot be entirely excluded but is markedly decreased in comparison to macrodissected samples. Our resulting expression data uncover a trajectory of transcriptional and proteomic dysregulation across tumor grades, suggesting non-genetic mechanisms as key contributors to ccRCC malignancy. Consistent with established ccRCC biology, our data recapitulate progressive metabolic rewiring with increasing grade, including repression of oxidative phosphorylation and TCA cycle activity, while glycolytic and HIF-associated programs are evident at the gene/protein level and become more pronounced at the pathway level upon multi-omics integration. The comparatively modest pathway-level dynamics of these processes likely reflect early VHL–HIF–driven reprogramming already present in low-grade tumors. Higher-grade and rhabdoid cells were characterized by more pronounced alterations in proliferative and ECM-related pathways. Within this context, we observed upregulation of FOXM1 and its downstream targets, consistent with increased FOXM1-associated transcriptional activity. Elevated FOXM1-mediated signals, previously linked to poor prognosis in ccRCC (Xue et al, 2012), point to an increased proliferative capacity of grade 4/rhabdoid cells. Concurrently, ECM reprogramming was evident through upregulation of integrins, which have been associated with adverse outcomes and enhanced tumor cell motility and dissemination (Chen et al, 2020; Morozevich et al, 2009; Li et al, 2023a). These mechanisms potentially involve the activation of matrix metalloproteinases (MMPs) (Arun et al, 2018). Moreover, these alterations align with known features of aggressive tumor behavior and may also relate to characteristic histopathological patterns of rhabdoid ccRCC, including their discohesive growth. Collectively, these observations highlight FOXM1-driven proliferative and ECM-related pathways as candidate vulnerabilities in high-grade ccRCCs.

While previous efforts often focused on sarcomatoid RCCs (Malouf et al, 2020; Bakouny et al, 2021), distinct molecular features of rhabdoid ccRCC remained underexplored. Bakouny and colleagues profiled the molecular landscapes in grade 4 ccRCC cells but did not identify alterations that distinguished rhabdoid differentiation (Bakouny et al, 2021). However, the majority of cases in their cohort comprised sarcomatoid RCCs, raising the question whether rhabdoid ccRCC is truly similar to or biologically distinct from sarcomatoid cases, as their morphologies suggest. Previous molecular investigations have associated the rhabdoid component with BAP1 mutations (Bakouny et al, 2021; Singh et al, 2015). However, in our genome-wide genetic profiling of five ccRCC tumors with rhabdoid features, BAP1 mutations were identified in only one case. Moreover, we did not observe the loss of chromosome 11p in our cohort, which has been reported for rhabdoid ccRCC (Perrino et al, 2015). Instead, our findings point to non-genetic alterations in a carefully selected cohort of ccRCCs with rhabdoid differentiation, based on the profiling of highly enriched, morphology-defined cell populations. Although based on a limited number of cases, the deep, multilayer profiling enabled by complementary transcriptomic and proteomic datasets allowed comprehensive characterization, demonstrating that higher-grade ccRCC cells increasingly separated from normal tubular kidney epithelial cells across both molecular layers. Owing to the rarity of rhabdoid ccRCC (Li et al, 2023b; Braun et al, 2021), particularly specimens with clearly differentiated and spatially matched grade 1/2, grade 3, and grade 4/rhabdoid components within the same tumor, our cohort represents a realistic foundation for exploratory molecular characterization. Based on sample size estimates, it is primarily powered to detect large expression changes in RNAseq and moderate-to-large changes in proteomics data, while more subtle effects may remain elusive. Despite these known constraints, the concordant regulation of key pathways across transcriptomic and proteomic layers supports the robustness of the observed molecular signatures. The level of concordance between both data layers is in line with prior observations (Vogel and Marcotte, 2012; Liu et al, 2016; Gonçalves et al, 2017) and was high at the pathway level, reflecting coordinated regulation of biological processes.

Notably, we identified a rhabdoid cell-specific overexpression network associated with the immune response. This involved established immune checkpoint receptors like PD-L1 but also additional factors such as CD38 and ITGB2 (Chen et al, 2018; Choueiri and Motzer, 2017; Xu et al, 2022). ITGB2 stands out as with a dual role, regulating the immune response while also contributing to the integrin–ECM signaling axis (Xu et al, 2022; Hamidi and Ivaska, 2018). CD38 is a multifunctional enzyme involved in NAD metabolism and has context-dependent roles across different cell types, including immune regulation (Piedra-Quintero et al, 2020). In tumor cells, CD38 has been linked to adenosine-mediated immune modulation and may contribute to altered T-cell function (Chen et al, 2018), although its role in ccRCC beyond the immune compartment remains insufficiently characterized. In support of a broader immunosuppressive program, we observed elevated expression of STAT1/2 and IFN-I signaling in rhabdoid ccRCC cells, both of which have been implicated in immune evasion, EMT activation, and an inflammatory phenotype (Langbein et al, 2022; Musella et al, 2017). STAT1/2 in particular are well-recognized markers of aggressiveness in ccRCC (Qu et al, 2022). These findings suggest that rhabdoid ccRCC cells themselves may contribute to shaping the local immune microenvironment (Pang et al, 2023) and influencing CD8⁺ T-cell function (Braun et al, 2021; Bi et al, 2021). In support of this, CD8⁺/TOX co-staining revealed expression of the exhaustion-associated marker TOX in the majority of our grade 4/rhabdoid ccRCC cases, indicating a T-cell state consistent with dysfunction. Such an inflamed yet altered immune context may relate to the responsiveness of rhabdoid ccRCC to ICI treatment (Chahoud et al, 2020), although our data did not allow conclusions regarding treatment benefit. Mechanistically, factors expressed by rhabdoid tumor cells, including PD-L1, CD38, and integrin-associated pathways, may contribute to this TME landscape. In particular, as CD38 has been implicated in NAD⁺ metabolism and the generation of immunomodulatory metabolites (Chen et al, 2018), providing a potential link between tumor-intrinsic expression programs and altered T-cell activity. These observations suggest that pathways involving CD38 and ITGB2 may represent potential avenues for further investigation, including in the context of combination strategies with ICI in aggressive rhabdoid ccRCC.

Previous studies have demonstrated the profound genetic ITH in ccRCC tissue samples (Turajlic et al, 2018b; Gerlinger et al, 2012) but were unable to directly relate these alterations to morphological features. Here, we integrate AI-driven image analysis, morphology-guided cell isolation, and deep multi-omics profiling to characterize molecular alterations in defined morphological phenotypes. The resulting expression profiles recapitulate known ccRCC tumorigenic changes (The Cancer Genome Atlas Research Network, 2013; Wettersten et al, 2017), supporting the accuracy of mRNA and protein dysregulation portraits obtained from as few as 1000 individually collected ccRCC cells from diagnostic FFPE sections. Our cohort size is limited, but analysis of independent datasets (The Cancer Genome Atlas Research Network, 2013; Braun et al, 2020) revealed similar numbers of rhabdoid cases with accompanying molecular data. Nevertheless, they demonstrated concordant FOXM1, CD38, and ITGB2 upregulation in grade 4/rhabdoid cells, supporting the relevance of our observations. In summary, our study addresses the gap between morphological heterogeneity and underlying molecular alterations in ccRCC by providing a morphology-resolved, multi-omics view of distinct tumor cell states. Limitations of this study include the absence of direct functional validation and the restricted scope of external datasets, which preclude conclusions regarding therapeutic response. Nevertheless, the integration of image-guided cell selection with multilayer molecular profiling establishes a framework for dissecting clinically relevant tumor heterogeneity and highlights candidate pathways for further investigation in aggressive ccRCC.

## Methods

**Table:** Reagents and tools table

Reagent/resource	Reference or source	Identifier or catalog number
**Experimental models**		
USZ FFPE tissue	University Hospital Zürich	n/a
TCGA-KIRC cohort	The Cancer Genome Atlas Research Network, 2013	https://doi.org/10.1038/nature12222 https://portal.gdc.cancer.gov/projects/TCGA-KIRC?
Checkmate025 cohort	Braun et al (2020)	https://doi.org/10.1038/s41591-020-0839-y
**Recombinant DNA**		
n/a		
**Antibodies**		
Anti-CD8 (Clone C8/144B)	DAKO	Cat# M7103
Anti-CDH1 (E-cadherin clone 36)	BD Biosciences	Cat# 610181
Anti-CD38 (SPC32)	Novocastra Laboratories	NCL-L-CD38-290
Anti-ITGB2	Sigma	HPA016894
Anti-TOX	Invitrogen	PA5-53781
**Oligonucleotides and other sequence-based reagents**		
SMARTer Stranded Total RNA-Seq Kit v2 – Pico Input Mammalian	Takara Bio	Cat# 634411
TruSeq DNA PCR Free Library Prep protocol HT	Illumina	Cat# 20015963
**Chemicals, enzymes, and other reagents**		
Xylene	Thermo Fisher Scientific	Cat# 253-VL03K
Ethanol	Thermo Fisher Scientific	Cat# 180-VL03K
RNAlater	Qiagen	Cat# 12559069
Triethylammonium bicarbonate (TEAB)	Sigma-Aldrich	Cat# T7408
Acetonitrile	Thermo Fisher Scientific	Cat# A955
Trifluoroacetic acid (TFA)	Thermo Fisher Scientific	Cat# 28904
Tris-Cl	Thermo Fisher Scientific	Cat# J67233.22
Tween-20	Thermo Fisher Scientific	Cat# P6585
LysC	WAKO	Cat# 129-02541
Trypsin	Promega	Cat# V5111
Maxwell® 16 FFPE Tissue LEV DNA Purification Kit	Promega	Cat# A826E
Optiview DAB IHC Detection Kit	Roche Ventana	Cat# 760-700
Bond Polymer Refine Detection Kit	Leica Biosystems	Cat# DS9800
RNeasy FFPE kit	Qiagen	Cat# 73504
**Software**		
DRAGEN Bio-IT Platform	Illumina	v3.8.4 and v3.9.5
R	R Foundation	v4.2.0
Bioconductor	Bioconductor	v3.15
Variant Effect Predictor	Ensembl	https://doi.org/10.1186/s13059-016-0974-4
maftools (R package)	Bioconductor	v2.12.0
GISTIC	Broad Institute	v2.0.23
BIAS software suite	Single-Cell Technologies	–
NDPI split		https://doi.org/10.1186/1746-1596-8-92
SUSHI	Hatakeyama et al (2016)	https://doi.org/10.1186/s12859-016-1104-8
FastQC	Babraham Bioinformatics	v0.11.9
STAR (Spliced Transcripts Alignment to a Reference)	Dobin et al (2013)	https://doi.org/10.1093/bioinformatics/bts635
GENCODE reference	GENCODE	GRCh38 release 42
Rsubread (featureCounts)	Bioconductor	v2.10.5
DESeq2	Bioconductor	v1.36.0
clusterProfiler	Bioconductor	v4.4.4
exploreDE	Leary and Rehrauer, 2023	https://doi.org/10.5281/zenodo.8167437
DIA-NN	Demichev et al (2020)	v1.8.1.8 https://doi.org/10.1038/s41592-019-0638-x
prolfqua (R package)	Wolski et al, 2023	https://doi.org/10.1021/acs.jproteome.4c00911
Ensembl homo_sapiens database	Ensembl	release-108
NOODAI platform	Totu et al, 2025	v1.0.0 https://doi.org/10.1093/bioinformatics/btaf553
STRING	STRING	v11.5
BioGRID	BioGRID	v4.4.218
IntAct	Del Toro et al (2022)	https://doi.org/10.1093/nar/gkab1006
MONET toolbox	Tomasoni et al (2020)	https://doi.org/10.1093/bioinformatics/btaa236
Cytoscape	Cytoscape	v3.10.1
Reactome		https://doi.org/10.1093/nar/gkad1057
KEGG	Kanehisa et al (2023)	https://doi.org/10.1093/nar/gkac963
GEPIA	Tang et al (2017)	https://doi.org/10.1093/nar/gkx247
RColorBrewer (R package)	CRAN	v1.1.3
readr (R package)	CRAN	v2.1.4
stringr (R package)	CRAN	v1.5.0
gtools (R package)	CRAN	v3.9.4
gridBase (R package)	CRAN	v0.4.7
ComplexHeatmap (R package)	Bioconductor	v2.12.1
tidygraph (R package)	CRAN	v1.2.3
biomaRt (R package)	Bioconductor	v2.56.1
readxl (R package)	CRAN	v1.4.3
**Other**		
PEN MembraneSlides 1.0	Carl Zeiss	415190-9081-000
PCR caps	VWR	732-0548
twin.tec PCR plate (384-well)	Eppendorf	EP0030128508
Sealing tape	Corning	CLS6569-100EA
Evotips	Evosep Biosystems	EV-2001
AVENIO Millisect System	Roche	–
Maxwell® 16 machine	Promega	AS2000
Covaris LE220-plus	Covaris	–
iSeq100	Illumina	–
NovaSeq 6000	Illumina	–
Hamamatsu C9600 scanner	Hamamatsu	–
LMD6 microscope	Leica	–
TapeStation	Agilent	–
Bio-Rad S1000 thermocycler	Bio-Rad	–
Mastercycler X50a	Eppendorf	–
Evosep One LC	Evosep Biosystems	–
timsTOF Pro	Bruker	–
Ventana Benchmark automated system	Roche/Ventana	–
Leica BOND system	Leica Biosystems	–

### Patient cohort

The Department of Pathology and Molecular Pathology at University Hospital Zürich made available archival FFPE tissue samples for this study. The study was approved by the local Ethics Committee (BASEC# 2019-01959) and conducted in accordance with Swiss law (Swiss Human Research Act), the principles of the World Medical Association Declaration of Helsinki, and the ethical principles outlined in the Belmont Report. All patients gave written informed consent. The tumor samples used in this study were diagnosed as ccRCC, and the overall grade was noted from the diagnostic histopathologic patient reports according to the WHO/ISUP (Moch et al, 2016). Two board-certified pathologists re-assessed all FFPE tissue samples in the context of this study to confirm the presence of malignant and normal areas as well as different grade morphologies. Associated clinical information is shown in Table 1 and Dataset EV1.

Post hoc sample size and power calculations were performed for the core cohort of five patients used for RNAseq and proteomics using variability estimates (biological coefficient of variation for RNAseq and trimmed protein-level standard deviation for proteomics). Assuming a two-sided significance level of α = 0.05 and 80% power, we estimated the number of samples required per group to detect log2 fold changes (Δ) of 0.59, 1, and 2 (corresponding to 1.5×, 2×, and 4× changes). Variability was summarized at the median (50th percentile) across measured features. Based on these estimates, the available sample sizes per group are powered to detect large expression changes in RNAseq (~fourfold) and moderate-to-large changes in proteomics ( ≥ twofold).

### Genomic characterization by whole-genome sequencing (WGS)

To extract tumor and normal DNA from our core cohort, we macrodissected areas encompassing all regions included in the image analysis and single-cell isolation workflow to capture mutations across these sites and define the shared mutational background of each tumor. To facilitate this a 2 µm section was stained with hematoxylin and eosin (H&E) to evaluate the morphology and an experienced pathologist marked the areas for dissection (Fig. EV1). This slide was used as a reference image for the AVENIO Millisect System (Roche), and subsequently the marked areas were dissected from a consecutive unstained 10 µm section prepared on a glass slide. Tissue areas from at least two 10 µm sections were collected in a tube containing lysis buffer (Promega, A826E from Maxwell® 16 FFPE Tissue LEV DNA Purification Kit) and subjected to genomic DNA extraction on a Maxwell® 16 machine (Promega) following the manufacturer’s instructions.

Whole-genome sequencing libraries were constructed according to Illumina TruSeq DNA PCR Free Library Prep protocol HT (Illumina Inc., San Diego, CA, USA). This included: (1) fragmentation of 400 ng genomic DNA to 350 bp inserts by Covaris LE220-plus, (2) cleanup of fragmented DNA, (3) end repair, (4) removal of large and small DNA fragments, (5) 3′-end adenylation, and (6) adapter ligation. The resulting libraries were quantified and quality-assessed with the iSeq100 (Illumina). The samples were sequenced with the NovaSeq 6000 platform (Illumina) using S4 flow cells with 300 cycles (2 × 150 reads) and measured 2× to reach an average coverage of 30× for normal kidney tissue and 120× for the tumor fraction per patient.

The raw sequencing files (bcf files) were converted to fastq format and demultiplexed in a single step using Illumina’s bcl2fastq program via Illumina DRAGEN Bio-IT Platform (v3.8.4). First steps in secondary analysis, including read trimming, alignment to GRCh38, sorting, duplicate marking, and somatic variant calling were done using Illumina DRAGEN Bio-IT Platform (version 3.9.5) (Wright et al, 2017) or Mutect2 (Cibulskis et al, 2013) and Strelka2 (Kim et al, 2018). To identify and annotate somatic variants, we annotated and converted the VCF files into MAF format using VEP (McLaren et al, 2016). The MAF files were analyzed using Maftools in R (Mayakonda et al, 2018). In addition, Maftools was used for comprehensive analysis and visualization, including tumor mutational burden (TMB) assessment, mutational signature analysis, and identification of significantly mutated genes. Genome-wide copy number aberrations were visualized with gistic (v2.0.23) using default parameters and log-transformed segmentation files from DRAGEN as input.

### Sample preparation for morphological analysis

For each ccRCC case from our core cohort, we selected one to four representative FFPE blocks in order to include areas displaying low-grade, high-grade, and rhabdoid areas as well as normal kidney histology. Subsequently, 10 µm sections were prepared using PEN MembraneSlides 1.0 (Carl Zeiss) and stained with H&E. Slides were digitized scanning z-stacks with seven z-slices in 0.5 µm increments using a Hamamatsu C9600 scanner at ×40 optical magnification. The scanner resolution was 0.228 µm per pixel. The NDPI files generated by the Hamamatsu C9600 scanner were processed into a BIAS-compatible format as follows. First, the NDPI split tool was used to extract image data and generate non-overlapping tiles with a resolution of 1024 × 1024 pixels. Tiles were saved in TIFF format, preserving spatial information. After tile extraction, the Simple Focus tool was applied to automatically select the optimal focus plane. The algorithm computes a sharpness value for each image field with seven Z positions based on four-neighborhood pixel differences. These differences are accumulated into a single sharpness score, and the field with the highest sharpness value is selected.

### Image analysis

Using the BIAS software suite (Mund et al, 2022a), we developed a pipeline to assign every cell to one of the following classes (i) grade 1/2 (low-grade) ccRCC, (ii) grade 3 (high-grade) ccRCC, (iii) grade 4/rhabdoid ccRCC, (iv) normal kidney, (v) other/junk. This workflow was composed of several steps: segmentation of nuclei, specification of cytoplasmatic areas, pre-classification into tumor and normal kidney cells, segmentation of nucleoli, segmentation of rhabdoid cells, and classification into the distinct classes (i–v) mentioned above.

We used Artificial Neural Networks (ANN) for both segmentation and classification. The classification models were multilayer perceptron networks (MLPs) trained on features extracted from the segmented cells. To segment nuclei, we integrated nucleAIzer models into BIAS (Hollandi et al, 2020). Dilating the nuclear contours by 10 µm created the cytoplasmic masks that were used to extract features from the cytoplasm. These were aggregated with features of the 10 neighboring cells, and an artificial neural network pre-classified the tissue into healthy and tumor.

Instance segmentation algorithms based on a Mask-RCNN implementation with a Resnet101 backbone achieved custom segmentation of prominent nucleoli and rhabdoid cells. Additional cases were annotated as training data (Dataset EV1). Subsequently, cRCC cells were classified into grade 1/2, grade 3, grade 4/rhabdoid, normal kidney or other/junk by MLP classification models trained on features extracted from the segmented cells. The classification models relied on explicitly extracted morphological and contextual features derived from nuclear, cytoplasmic, and neighborhood characteristics, enabling biologically interpretable phenotype separation rather than relying solely on end-to-end black-box predictions. Phenotype predictions were ranked by confidence, and the 2000 best cells were manually curated. For performance evaluation (testing), K-fold stratified cross-validation (where *K* = 10) was performed using the annotated cell dataset of the core patient cohort (Table 1). In each iteration, nine folds were used for training and the remaining fold for validation. Annotated cells were partitioned into stratified 10-fold cross-validation splits, ensuring balanced representation of all morphological classes across folds while preventing over-representation of cells from the same image region. Moreover, we evaluated the generalization of our approach using five independent grade 4 ccRCC cases with rhabdoid morphology and grade ITH (Dataset EV1).

### Sample preparation and single-cell isolation

Cell selection and laser microdissection were performed on the same HE-stained section, avoiding the need for registration to adjacent sections. After generation of WSIs, HE-stained FFPE tissue sections on PEN MembraneSlides were subjected to coverslip removal by immersing them in xylene for 1–2 h (until the coverslip loosened) followed by xylene removal and hydration using descending ethanol concentrations and air-drying. Subsequently, cells were isolated by LMD based on the morphologies assigned by the machine learning classification module of BIAS in the same sections. Relevant cell contours were transferred to a Leica LMD6 microscope for automated single-cell isolation. The Leica LMD6 system, equipped with an HC PL FLUOTAR L ×63/0.70 objective, was used for cell excision. Cells were excised based on nuclei contours plus a 6 µm offset to approximate the cytoplasm, resulting in highly enriched epithelial cell populations. For each morphological class in each sample, two sets of 1,000 cells were microdissected and collected into PCR caps (VWR, 732-0548) or 384-well plates (Eppendorf® Twin tec PCR, 384 wells EP0030128508)

To cells isolated for RNAseq analysis, 5 μl of PKD buffer (Qiagen) and 25 μl of RNA later (Qiagen) were added, followed by centrifugation at 10,000× *g* for 5 min. For proteome analysis, we added 10 μl of ammonium bicarbonate buffer to the isolated cells.

### RNA sequencing and data analysis

RNA was extracted from microdissected single cells using the RNeasy FFPE kit (Qiagen, Hilden, Germany). Entire RNA extracts were transferred into NGS Library preparation without QC. Due to the low concentration of total RNA the SMARTer® Stranded Total RNA-Seq Kit v2 -Pico Input Mammalian (Takara, California, USA) was used to prepare sequencing libraries.

Briefly, total RNA samples were reverse-transcribed using random priming into double-stranded cDNA in the presence of a template switch oligo (TSO). When the reverse transcriptase reached the 5’ end of the RNA fragment, the enzyme’s terminal transferase activity added non-templated nucleotides to the 3’ end of the cDNA. The TSO paired with the added non-templated nucleotide, enabling the reverse transcriptase to continue replicating to the end of the oligonucleotide. This resulted in a cDNA fragment that contained sequences derived from the random priming oligo and the TSO. PCR amplification using primers binding to these sequences could then be performed. The PCR added full-length Illumina adapters, including dual barcodes for multiplexing. Ribosomal cDNA was cleaved by ZapR in the presence of the mammalian-specific R-Probes. Remaining fragments were enriched with a second round of PCR amplification using primers designed to match Illumina adapters (16 cycles). The quality and quantity of the enriched libraries were validated using the Tapestation (Agilent, Waldbronn, Germany). The product was a smear with an average fragment size of ~300–360 bp. The libraries were normalized to 5 nM in Tris-Cl 10 mM, pH 8.5, with 0.1% Tween-20.

A shallow sequencing approach on the iSeq (Illumina, Inc, California, USA) was used to check the read distribution among samples, rRNA content, and reference genome mapping. The Novaseq 6000 (Illumina, Inc, CA, USA) was used for deep sequencing according to the standard manufacturer’s protocol. Sequencing configuration was single-end 100 bp. Demultiplexing was performed with bcl2fastq (Illumina, Inc, CA, USA) allowing for 0 mismatch on Index 1 and 1 mismatch on Index 2. Between 70 million and 146 million reads were generated per sample.

RNA sequencing data analysis was performed using the SUSHI framework (Hatakeyama et al, 2016). The analysis encompassed the following steps: Read quality was inspected using FastQC, and sequencing adapters were removed using fastp; Alignment of the RNASeq reads using the STAR aligner (Dobin et al, 2013) and with the GENCODE human genome build GRCh38 (Release 42) as the reference (Frankish et al, 2021); the counting of gene-level expression values using the “featureCounts” function of the R package Rsubread (Liao et al, 2013). Genes with fewer than 10 total counts across samples were excluded prior to differential expression analysis. Differential expression was performed the DESeq2 Bioconductor package (Love et al, 2014) which applies size-factor normalization, dispersion estimation, and generalized linear modeling of count data. Gene Ontology (GO) term pathway analysis was performed using hypergeometric over-representation and GSEA tests *via* the “enrichGO” and “gseGO” functions, respectively, of the clusterProfiler Bioconductor R package (Yu et al, 2012).

As RNAseq libraries were generated from FFPE-derived total RNA without polyA-enrichment, a substantial fraction of reads mapped to intronic or intergenic regions that do not overlap annotated gene features and are therefore not counted during gene-level quantification. However, the high sequencing depth (58–170 million STAR input reads per sample) ensured sufficient read counts (2–12 million counted reads per sample) for robust differential expression analysis. LOESS bias assessment confirmed that differential expression estimates were not systematically influenced by gene length or GC content. Principal component analysis (PCA) and sample correlation analysis confirmed that biological variation between morphological groups exceeded technical variability.

For differential gene expression analysis (DEG), we used an unpaired comparison design, contrasting gene expression in cRCC grade groups with that in the normal renal tubuli samples to obtain three ccRCC-specific comparisons: (i) grade 1/2 (low-grade), (ii) grade 3 (intermediate/high-grade, and (iii) grade 4/rhabdoid. Additional visualizations were generated in the exploreDE Interactive Shiny app (Leary and Rehrauer, 2023). All R functions were executed on R version 4.2.0 (R Core Team, 2020) and Bioconductor version 3.15.

### Proteomics data acquisition and analysis

For sample lysis, the collected cells were spun down, and 20 μl of 50 mM Triethylammonium bicarbonate (TEAB) was added to each tube or well. The 384-well plate was closed with sealing tape (Corning, CLS6569-100EA) and centrifuged at 2000×*g* for 2 min. All samples were incubated for 1 h at 95 °C. For the plates a PCR thermo cycler with 384-well reaction module (Bio-rad S1000) was used, for the PCR tubes, the 96-well module (Eppendorf, Mastercycle X50a) was installed. The lid temperature for all incubation steps was always 110 °C. Prior to incubation for 60 min at 75 °C, 5 µl of 60% acetonitrile in 50 mM TEAB was added. For in-solution protein digestion, samples were cooled to room temperature and subjected to pre-digestion using 1 µl LysC (4 ng/µl) for 4 h at 37 °C. Subsequently, 1 µl Trypsin (4 ng/µl in TEAB) was added for overnight digestion at 37 °C. In the morning, another 4 ng of Trypsin was added, and the samples were incubated for 5 h at 37 °C (Mund et al, 2022b). The digestion was stopped by acidification, adding 10 µl 5% aqueous trifluoroacetic acid, and the sample were stored at −20 °C until LC-MS analysis.

For proteomics data acquisition, half of the digested samples were loaded on Evotips (Evosep Biosystems) following the provided instructions and analyzed on an Evosep One LC coupled to a TIMS TOF Pro mass spectrometer (Bruker). The mass spectrometry proteomics data were handled using the local laboratory information management system b-fabric (LIMS) (Panse et al, 2022), and all relevant data have been deposited to the ProteomeXchange Consortium via the PRIDE (http://www.ebi.ac.uk/pride) partner repository with the dataset identifier PXD061011.

The acquired data-independent-acquisition (DIA) spectra were processed with DIA-NN (Version 1.8.1.8) (Demichev et al, 2020), using a library-free approach with the ensembl homo_sapiens database (release-108) and oxidation at methionine as variable modification. The R package prolfqua (prolfqua: A Comprehensive R-Package for Proteomics Differential Expression Analysis | Journal of Proteome Research) was used to process, handle, and analyze the differential expression and to determine group differences, confidence intervals, and false discovery rates for all quantifiable proteins. After exclusion of samples that did not meet quality control criteria, specifically samples with substantially lower numbers of quantified proteins and distinct clustering patterns indicative of technical variation, differential protein expression (DEP) analysis was performed on the remaining high-quality samples using an unpaired comparison design across the five samples per morphological group. Starting with the report.tsv files generated by DIA-NN, which included the precursor ion abundances for each raw file, we determined gene centric abundances by first aggregating the precursor abundances to peptidoform abundances after filtering for DIA-NN protein-level *q*-values (Lib.PG.Q.Value/PG.Q.Value < 0.01). Then, we employed the Tukeys-Median Polish to estimate gene abundances. This was possible with a mapping table where for each ensemble protein the gene name was listed (if there was no gene name listed, the ensemble protein identifier was used). Using the gene names instead of the protein identifier allowed us to roll up the peptidoform abundances to gene names. Furthermore, before fitting the linear models, we transformed the gene abundances using the log2 transformation followed by robust scaling normalization. Missing proteomics measurements were handled within the prolfqua framework without direct abundance imputation; for proteins completely absent in one experimental condition, the limit of quantification (LOQ) was estimated and used to approximate the mean of the missing condition, enabling statistical testing while avoiding imputation of abundance values.

Differential protein expression (DEP) analysis was performed using moderated linear models within the prolfqua framework, and statistical significance was assessed using Benjamini–Hochberg false discovery rate (FDR) correction. We contrasted protein expression in each ccRCC grade to that in the normal renal tubuli samples to obtain three ccRCC-specific comparisons: (i) grade 1/2 (low-grade), (ii) grade 3 (intermediate/high-grade, and (iii) grade 4/rhabdoid. Principal component analysis was used to confirm that biological variation between morphological classes exceeded technical variability. Gene Ontology (GO) term pathway analysis was performed in the same way as in the RNA sequencing data analysis pipeline using the hypergeometric over-representation and GSEA tests *via* the “enrichGO” and “gseGO” functions respectively from the clusterProfiler Bioconductor R package (Yu et al, 2012).

### Immunohistochemistry (IHC)

Sections (2 µm) were prepared from FFPE specimens and were stained with hematoxylin and eosin (H&E) for histological evaluation. For IHC, sections (2 μm) were transferred to glass slides, followed by antigen retrieval. IHC was performed using the Ventana Benchmark automated system (Roche, Ventana Medical Systems, Oro Valley, AZ) and Ventana reagents. The Optiview DAB IHC detection kit (Roche, Ventana Medical Systems) was used to stain with the antibody against CD8 (Clone C8/144B, cat. number M7103, DAKO, used 1:100). We used the automated Leica BOND system with the Bond Polymer Refine Detection Kit (Leica Biosystems, Wetzlar, Germany) staining for CD38 (Anti-CD38 SPC32 from Novocastra Laboratories, cat. number NCL-L-CD38-290, 1:100), for ITGB2 (Anti-ITGB2 form Sigma, cat. number HPA016894, 1:100) and for E-Cadherin (Anti-CDH1, E-cadherin clone 36 form BD Biosciences cat. number 610181, 1:500). CD8/Tox co-stain was previously described (Anti-TOX from Invitrogen cat. number PA5-53781, 1:200, and Anti-CD8 Clone C8/144B, cat. number M7103, DAKO, used 1:400) (Sobottka et al, 2024).

### Survival analysis

Survival curves were generated using the Gene Expression Profiling Interactive Analysis (GEPIA) web tool (http://gepia.cancer-pku.cn) to assess the prognostic significance of gene expression levels for CDH1, FOXM1, and ITGA4 in the TCGA Kidney Renal Clear Cell Carcinoma (TCGA-KIRC) patients. Patients were divided into high and low expression groups based on the median expression level. Kaplan–Meier survival curves were generated, and the log-rank test was used for statistical comparison. Hazard ratios (HR), 95% confidence intervals (CI), and *P* values were calculated and displayed. Survival differences were considered significant if *P* values were <0.05. HRs >1 indicated a higher risk, while HRs <1 indicated a protective effect.

### Public dataset and external validation analyses

Patient and upper-quartile (UQ) normalized transcripts-per-million (TPM) RNAseq data from CheckMate 025 (CM-025; NCT01668784) prospective clinical trial of the anti-PD-1 antibody nivolumab in ccRCC were obtained from the accompanying paper (Braun et al, 2020). CM-025 was a randomized phase III trial that demonstrated an improved overall survival (OS) with nivolumab over the mTOR inhibitor everolimus. Patients were included based on the availability of RNAseq data and information on rhabdoid differentiation. After excluding cases with incomplete annotations, CDH1, FOXM1, CD38, and ITGB2 expression levels were plotted and analyzed in R.

RNAseq normalized gene expression values and clinical data were obtained from the TCGA-KIRC cohort (Data ref: Genomic Data Commons TCGA-KIRC, 2013). and analyzed in R. Tumor grade annotation was used to stratify samples, with Grade 4 tumors serving as a surrogate for aggressive disease states, as rhabdoid differentiation is not explicitly annotated. Normalized gene expression values were used to assess differential expression of selected markers (FOXM1, CD38, ITGB2, CDH1). Overall survival analyses were performed using Kaplan–Meier estimates and log-rank tests, with patients stratified by median expression or composite score. Tumor microenvironmental context was assessed using the ESTIMATE algorithm (Yoshihara et al, 2013), which infers immune-cell admixture from bulk transcriptomic data. Patients were stratified into high and low immune score groups based on the median score.

### Statistical testing

We performed differential expression analysis (DEG/DEP) comparing ccRCC grade groups (grade 1/2, grade 3, grade 4/rhabdoid) to normal kidney tubule cells. To assess significant changes, we applied a false discovery rate (FDR) threshold of <0.05 and considered log2 fold change (log2FC) greater than |1 | . The significance levels for FDR are denoted as follows: **** <= 0.0001, *** <= 0.001, **<= 0.01, * <= 0.05. In addition, we color-coded the log2FC values for visual representation.

For enrichment analysis in the transcriptomic and proteomic datasets, we conducted over-representation analysis using the hypergeometric test. Specifically, we examined Gene Ontology (GO) biological pathways. Benjamini–Hochberg adjusted *P* values were calculated, and Significance levels are denoted as follows: **** for *P* <= 0.0001, *** for *P* <= 0.001, ** for *P* <= 0.01, * for *P* <= 0.05.

### Data integration by PPI network analysis

For comparison of proteomic and transcriptomic datasets, we used the results from the differential expression and GO enrichment analyses performed for each data type independently between the normal and the three grades of the ccRCC. Clustering plots were generated using all genes with a standard deviation in log2 fold change between samples of at least 0.5 and a maximum of 2000 genes.

To highlight the synergies between the measured proteomics and transcriptomics profiles, we performed integrated network analysis using the NOODAI software platform (Totu et al, 2025). Differentially expressed elements of the proteomics and transcriptomics analysis were used as input and searched against the default publicly available protein–protein interaction (PPI) databases containing only high-confidence human interactors from STRING (Szklarczyk et al, 2019), BioGRID (Oughtred et al, 2021), and IntACT (Kerrien et al, 2012) databases were collected. For STRING (version 11.5), complete interactome data from all sources were filtered to include only entries with a combined score above 0.7. For BioGRID (version 4.4.218), a mitab data file that contained a dataset of interactors with physical interactions supported by independent validations was used. It was filtered to exclude entries without an associated confidence value. For the IntACT database, the psimitab from 13/07/2022 was used and filtered to keep only interactions with a confidence score above 0.7. Interaction pairs obtained from the different databases were overlapped and merged into a joint dataset. This was used in NOODAI to construct a network, in which only interaction pairs with both members significantly differentially expressed in at least one of the data layers (transcriptomics or proteomics) were retained. Only the network with the highest number of connected members was further analyzed (without allowing edge repetition). In order to identify highly connected central nodes in the networks, the default current flow betweenness centrality metric was used (Newman, 2005).

Functional network modules (Choobdar et al, 2019) were subsequently identified through NOODAI by using the MONET software (Tomasoni et al, 2020). Modularity optimization with undirected edges was selected, setting the target average node degree within the identified modules to 10. The modules were default-sorted based on the number of nodes they contained. To assess the robustness of the network decomposition and to identify the main signaling axes present in our PPI networks, NOODAI performed pathways over-representation analysis for individual modules by searching against the pre-loaded Reactome and KEGG databases. By default, background datasets were composed of all genes and proteins that were used to construct the full-scale network of the respective conditions. Results were filtered to retain significant pathways with at least five significant hits and an FDR below 0.05 (Benjamini–Hochberg (Benjamini and Hochberg, 1995) correction of Fisher’s exact test *P* values)., Non-specific pathways with more than 300 members were omitted. Pathways that did not have more than two different overrepresented members were considered redundant and were investigated in the context of their explanatory power. For the visualization of results, NOODAI generated circular charts that highlighted the top 30% of most central nodes (based on the current-flow betweenness centrality). For selected modules and full-size networks, the graphical representation was done in Cytoscape 3.10.1 (Shannon et al, 2003) using the webtool-generated edge files. For edge routing, the *organic routing* from yFiles plugin was used (yWorks Gmb, 2024). Additional R packages used for data handling and visualization of the NOODAI results included RColorBrewer (Neuwirth and Brewer, 2014), readr (Wickham et al, 2023b), stringr (Wickham and Wickham, 2019), gtools (Bolker and Warnes, 2022), gridBase (Murrell, 2014), ComplexHeatmap (Gu et al, 2016), tidygraph (Pedersen, 2023), biomaRt (Durinck et al, 2009), and readxl (Wickham and Bryan, 2023). For visualization purposes, representative genes were selected from biological pathways in enriched modules based on statistical significance and biological relevance.

## Supplementary information

Peer Review File

Dataset EV1

Dataset EV2

Expanded View Figures

## Data Availability

Histological images used for automated image analysis and cell classification are deposited in FigShare using the following link: https://doi.org/10.6084/m9.figshare.c.7682516. A demo dataset and online material of how to install BIAS and reproduce our work can be accessed at https://drive.google.com/drive/folders/13QlATL8r9TW47tDxjnJ2owfNT4C2PwOs?usp=drive_link. Whole-genome sequencing (WGS) and RNAseq data were generated under a de-identified protocol that does not permit deposition in a public repository. It can be made available from the corresponding authors upon reasonable request. RNAseq read count data have been deposited in Zenodo together with the accompanying code https://doi.org/10.5281/zenodo.14922387. The mass spectrometry proteomics data have been deposited to the ProteomeXchange Consortium via the PRIDE (Perez-Riverol et al, 2025) partner repository with the dataset identifier PXD061011. H&E and IHC WSI are available via the BioImage Archive under accession number S-BIAD3059 and can be accessed using this https://doi.org/10.6019/S-BIAD3059. A free compiled version of BIAS with limited high-throughput capabilities is available at https://drive.google.com/drive/folders/13QlATL8r9TW47tDxjnJ2owfNT4C2PwOs?usp=drive_link containing all features applied in the described workflows. Algorithms used for omics data analysis are publicly available from the indicated references in the Methods section. Specialized code used for RNAseq and proteomics data analysis in this study were deposited in Zenodo: https://doi.org/10.5281/zenodo.14922387. The code for multi-omics data integration analysis as well as for automated network representations in Cytoscape can be found on GitHub at https://github.com/TotuTiberiu/NOODAI and corresponds to version 1.0.0 of the NOODAI platform (Totu et al, 2025) that can be freely accessed at https://omics-oracle.com/NOODAI (including instructions and tutorials). The source data of this paper are collected in the following database record: biostudies:S-SCDT-10_1038-S44321-026-00484-8.
