# Supplementary material for: Deep visual multi-omics profiling links morphology and molecular programs in clear cell renal cell carcinoma
Source: EMBO Mol Med. 2026 Aug 6;18(9):3850–77. doi: 10.1038/s44321-026-00484-8 (PMC13562643; doi:10.1038/s44321-026-00484-8)
Supplement: Supplementary file 1 [file 44321_2026_484_MOESM1_ESM.pdf]

EMBO Molecular Medicine

Deep visual multi-omics links morphology and molecular programs in clear cell renal cell carcinoma

Hella Bolck, Ede Migh, Andras Kriston, Natalia Zajac, Susanne Kreutzer, Tiberiu Totu, Peter Leary, Ferenc Kovacs, Dorothea Rutishauser, Sibylle Pfammatter, Jonas Grossmann, Harini Lakshminarayanan, Debleena Basu, Cassandra Litchfield, Marija Buljan, Niels Rupp, Peter Horvath, and Holger Moch

Corresponding authors: Peter Horvath (horvath.peter@brc.hu) , Holger Moch (holger.moch@usz.ch), Hella Bolck (hella.bolck@zhaw.ch)

Review Timeline:

Submission Date:	8th Oct 25
Editorial Decision:	3rd Nov 25
Revision Received:	9th Apr 26
Editorial Decision:	25th May 26
Revision Received:	12th Jun 26
Accepted:	1st Jul 26

Editor: Lise Roth

Transaction Report:

(Note: With the exception of the correction of typographical or spelling errors that could be a source of ambiguity, letters and reports are not edited. Depending on transfer agreements, referee reports obtained elsewhere may or may not be included in this compilation. Referee reports are anonymous unless the Referee chooses to sign their reports.)

3rd Nov 2025

Dear Dr. Horvath,

Thank you for submitting your manuscript to EMBO Molecular Medicine. We have now received feedback from the three reviewers who agreed to evaluate your manuscript. As you will see from the reports below, the reviewers acknowledge the innovative nature of the study and its interest, but they also raise concerns relating to the small cohort size, missing methodological information, and the lack of experimental validation and quantification.
If you feel you can satisfactorily address the points raised by the referees, you may wish to submit a revised version of your manuscript. Please note that adding new samples will NOT be required if this is not feasible within the scope of these revisions; however, this limitation should be clearly mentioned and discussed.

Addressing the reviewers' concerns in full will be necessary for further considering the manuscript in our journal, and acceptance of the manuscript will entail a second round of review. EMBO Molecular Medicine encourages a single round of revision only and therefore, acceptance or rejection of the manuscript will depend on the completeness of your responses included in the next, final version of the manuscript. For this reason, and to save you from any frustrations in the end, I would strongly advise against returning an incomplete revision.

If you would like to discuss further the points raised by the referees, I am available to do so via email or video. Let me know if you are interested in this option.

We are expecting your revised manuscript within three months, if you anticipate any delay, please contact us.

When submitting your revised manuscript, please carefully review the instructions that follow below. We perform an initial quality control of all revised manuscripts before re-review; failure to include requested items will delay the evaluation of your revision.

We require:

1) A .docx formatted version of the manuscript text (including legends for main figures, EV figures and tables). Please make sure that the changes are highlighted to be clearly visible.

2) Individual production quality figure files as .eps, .tif, .jpg (one file per figure). For guidance, download the 'Figure Guide PDF' (https://www.embopress.org/page/journal/17574684/authorguide#figureformat).

3) At EMBO Press we ask authors to provide source data for the main manuscript figures. You will receive a separate email with instructions for providing source data with your revised manuscript, including how to upload and organize the files.

Additional information on source data and instruction on how to label the files are available

4) A .docx formatted letter INCLUDING the reviewers' reports and your detailed point-by-point responses to their comments. As part of the EMBO Press transparent editorial process, the point-by-point response is part of the Review Process File (RPF), which will be published alongside your paper.

5) A complete author checklist, which you can download from our author guidelines (https://www.embopress.org/page/journal/17574684/authorguide#submissionofrevisions). Please insert information in the checklist that is also reflected in the manuscript. The completed author checklist will also be part of the RPF.

6) All Materials and Methods need to be described in the main text using our 'Structured Methods' format. According to this format, the Methods section includes a Reagents and Tools Table (listing key reagents, experimental models, software and relevant equipment and including their sources and relevant identifiers) followed by a Methods and Protocols section describing the methods, ideally using a step-by-step protocol format. The aim is to facilitate adoption of the methodologies across labs. Please download and fill our Reagents and Tools Table template (.docx), which you can find in our author guidelines: https://www.embopress.org/page/journal/14693178/authorguide#structuredmethods.
When submitting your revised manuscript, please do not include the Reagents and Tools Table in the Methods section of the manuscript but upload it as a separate file choosing the file type "Reagent Table".

7) Please note that all corresponding authors are required to supply an ORCID ID for their name upon submission of a revised manuscript.

8) It is mandatory to include a 'Data Availability' section after the Materials and Methods. Before submitting your revision, primary datasets produced in this study need to be deposited in an appropriate public database, and the accession numbers and

database listed under 'Data Availability'. Please remember to provide a reviewer password if the datasets are not yet public (see https://www.embopress.org/page/journal/17574684/authorguide#dataavailability).

In case you have no data that requires deposition in a public database, please state so in this section. Note that the Data Availability Section is restricted to new primary data that are part of this study.

9) For data quantification: please specify the name of the statistical test used to generate error bars and P values, the number (n) of independent experiments (specify technical or biological replicates) underlying each data point and the test used to calculate p-values in each figure legend. The figure legends should contain a basic description of n, P and the test applied. Graphs must include a description of the bars and the error bars (s.d., s.e.m.). Please provide exact p values.

10) Our journal encourages inclusion of *data citations in the reference list* to directly cite datasets that were re-used and obtained from public databases. Data citations in the article text are distinct from normal bibliographical citations and should directly link to the database records from which the data can be accessed. In the main text, data citations are formatted as follows: "Data ref: Smith et al, 2001" or "Data ref: NCBI Sequence Read Archive PRJNA342805, 2017". In the Reference list, data citations must be labeled with "[DATASET]". A data reference must provide the database name, accession number/identifiers and a resolvable link to the landing page from which the data can be accessed at the end of the reference. Further instructions are available at .

11) We replaced Supplementary Information with Expanded View (EV) Figures and Tables that are collapsible/expandable online. EV Figures should be cited as 'Figure EV1, Figure EV2" etc... in the text and their respective legends should be included in the main text after the legends of regular figures.
- For the figures that you do NOT wish to display as Expanded View figures, they should be bundled together with their legends in a single PDF file called *Appendix*, which should start with a short Table of Content. Appendix figures should be referred to in the main text as: "Appendix Figure S1, Appendix Figure S2" etc.
- Additional Tables/Datasets should be labeled and referred to as Table EV1, Dataset EV1, etc. Legends have to be provided in a separate tab in case of .xls files. Alternatively, the legend can be supplied as a separate text file (README) and zipped together with the Table/Dataset file.
See detailed instructions here:

12) The paper explained: EMBO Molecular Medicine articles are accompanied by a summary of the articles to emphasize the major findings in the paper and their medical implications for the non-specialist reader. Please provide a draft summary of your article highlighting
- the medical issue you are addressing,
- the results obtained and
- their clinical impact.
This may be edited to ensure that readers understand the significance and context of the research. Please refer to any of our published articles for an example.

13) Author contributions: CRediT has replaced the traditional author contributions section because it offers a systematic machine readable author contributions format that allows for more effective research assessment. Please remove the Authors Contributions from the manuscript and use the free text boxes beneath each contributing author's name in our system to add specific details on the author's contribution. More information is available in our guide to authors.

14) Disclosure statement and competing interests: We updated our journal's competing interests policy in January 2022 and request authors to consider both actual and perceived competing interests. Please review the policy https://www.embopress.org/competing-interests and update your competing interests if necessary.

15) Every published paper now includes a 'Synopsis' to further enhance discoverability. Synopses are displayed on the journal webpage and are freely accessible to all readers. They include a short stand first (maximum of 300 characters, including space) as well as 2-5 one-sentences bullet points that summarizes the paper. Please write the bullet points to summarize the key NEW findings. They should be designed to be complementary to the abstract - i.e. not repeat the same text. We encourage inclusion of key acronyms and quantitative information (maximum of 30 words / bullet point). Please use the passive voice. Please attach these in a separate file or send them by email, we will incorporate them accordingly.

Please also suggest a visual abstract to illustrate your article as a PNG file 550 px wide x 300-600 px high. A cropped portion of this image will serve as thumbnail for the table of content on our webpage.

16) As part of the EMBO Publications transparent editorial process initiative (see our Editorial at http://embomolmed.embopress.org/content/2/9/329), EMBO Molecular Medicine will publish online a Review Process File (RPF) to accompany accepted manuscripts.
In the event of acceptance, this file will be published in conjunction with your paper and will include the anonymous referee reports, your point-by-point response and all pertinent correspondence relating to the manuscript. Let us know whether you

agree with the publication of the RPF and as here, if you want to remove or not any figures from it prior to publication.
Please note that the Authors checklist will be published at the end of the RPF.

EMBO Molecular Medicine has a "scooping protection" policy, whereby similar findings that are published by others during review or revision are not a criterion for rejection. Should you decide to submit a revised version, I do ask that you get in touch after three months if you have not completed it, to update us on the status.

I look forward to receiving your revised manuscript.

Yours sincerely,

Lise Roth

Lise Roth, PhD
Senior Editor
EMBO Molecular Medicine

***** Reviewer's comments *****

Referee #1 (Comments on Novelty/Model System for Author):

The manuscript, "Deep visual multiomics profiling reveals mechanisms that underlie aggressiveness in clear cell renal cell carcinoma," presents an ambitious attempt to integrate AI-based morphologic segmentation with laser-capture microdissection and transcriptomic/proteomic profiling of morphologically distinct (based on nucleolar grade) ccRCC cells. The study's combination of digital pathology and multi-omics is technically sophisticated and could provide valuable insights into histologic heterogeneity and rhabdoid differentiation.
However, the work is exploratory in scope and limited by an extremely small cohort (n=5). Its eventual use of pooled rather than true single-cell data, and lack of independent or functional validation makes the results not robust. The conclusions, particularly regarding immune evasion mechanisms and therapeutic implications are therefore descriptive and hypothesis-generating rather than mechanistically established.
While the paper demonstrates an elegant methodological framework, its scientific robustness falls short of what would be required for mechanistic or translational claims. It may best fit as a technical or methodological resource paper if reframed appropriately and supported by additional validation.
I provide some feedback that may be helpful regarding the work-

1. Four regions were selected from five ccRCC cases for whole-genome sequencing, but Figure 1B and the associated results are presented at the patient level. Please clarify how mutations and chromosomal alterations from different regions or grades within the same patient were combined. If sequencing was performed on only one region per case, this should be clearly stated, along with the corresponding grade.
2. Machine learning models were applied for single-cell segmentation, yet quantitative performance metrics (accuracy, precision, recall, F1 score, etc.) are not provided. These are critical to assess the discriminatory ability of the models. Moreover, given that tumor cells in ccRCC are often surrounded by stromal and immune elements and that 20 μm sections were used for cell isolation the alignment of segmented cells to flanking H&E sections may introduce spatial inaccuracies. The subsequent merging of these segmented cells for analysis effectively approximates bulk RNA-seq. Since ccRCC grade-specific clones typically form discrete clusters (as shown in Supplementary Fig. 1B), it would be helpful to compare this approach to bulk sequencing from macrodissected grade-specific regions (or punch biopsies).
3. The differential gene and protein expression (DEG and DEP) analyses are technically neat, but the small number of samples limits statistical power and the reliability of the observed trends.
4. As shown in Supplementary Figure 2, inter-patient variability appears to dominate over grade-related differences. Please specify how this confounding factor was accounted for during the analyses.
5. Since normal controls were excluded from DEG/DEP analyses for at least two cases, please clarify how these comparisons were conducted. Were remaining normals pooled or substituted from other patients?
6. Gene selection in several figures including Figure 3C: Please describe the rationale for choosing the genes displayed in Figure 3C were these based on significance, pathway enrichment, or prior biological relevance?
7. Have any of the transcriptomic or proteomic findings-particularly discrepant ones-been validated using IHC or another orthogonal method to distinguish technical versus biological differences?
8. Label is missing for Fig 3A.

9. Prior studies have demonstrated progressive dysregulation of glycolytic and oxidative phosphorylation pathways with increasing tumor grade. Since your analysis does not reproduce these patterns, it would be helpful to address potential reasons for this discrepancy in the Discussion.
10. Validation of E-cadherin, CDH1, CD38, and related markers in larger datasets would strengthen the conclusions. It would be particularly valuable to determine whether expression of these markers varies between regions of different grades within the same patient.
11. Prognostic performance of FOXM1, ITGA4, and CDH1: In Supplementary Figures 4B and 5A/B, did these markers demonstrate superior prognostic discrimination compared with histologic grade alone?
12. The Discussion would benefit from placing these results in the context of previously published large-scale NGS studies that examined both grade and rhabdoid morphologies in ccRCC.
13. The authors are encouraged to tone down claims and reframe findings as hypothesis-generating.

Referee #1 (Remarks for Author):

This manuscript is methodologically creative however biologically seems underpowered. It serves more as a proof-of-concept than a robust mechanistic study. The AI, laser capture and omics pipeline is neat and could be impactful if validated on larger cohorts and linked to clinical outcomes. Currently, however, conclusions about rhabdoid morphology specific immune suppression and therapeutic implications are a bit overstated relative to sample size and data depth.

Referee #2 (Remarks for Author):

1.Insufficient sample size and generalizability. The principal findings are derived from five cases with morphology-defined subregions. Although the study demonstrates grade-progressive molecular dysregulation and an immunosuppressive program in rhabdoid-like cells, the small cohort and hierarchical (patient/subregion) structure may amplify random effects and limit external validity; increasing the sample size is recommended.

2.Inadequate reproducibility and generalization of the AI pathology-guided LCM pipeline. The closed loop of "AI-driven cellular grading → LCM-directed single-cell capture → deep multi-omics" is emphasized, but details on model training/validation splits, performance metrics (AUC, sensitivity, specificity, confusion matrices), failure cases, and interpretability evidence are lacking; independent generalization on external whole-slide images (WSI) is also not reported.

3.Insufficient quantification of multi-omics QC, missing-data handling, and cross-layer consistency. Separation and functional concordance between transcriptome and proteome are largely qualitative; systematic reporting of LCM cell counts, library/protein identification QC metrics, missing-value handling, and batch-effect control is absent; transcript-protein consistency lacks quantitative matrices and formal statistical testing.

4.Immune biology conclusions lack direct functional and spatial corroboration. The claim that rhabdoid-like tumor regions exhibit an immunosuppressive network (IFN-β, PD-L1, CD38, ITGB2) in a CD8-rich context-potentially driving T-cell exhaustion/functional suppression-relies mainly on expression, pathway, and network evidence, without functional readouts (e.g., spatial co-localization of exhaustion markers Ki67/GZMB/TOX/TIM-3/PD-1) or perturbation data. mIHC/IMC or spatial transcriptomics should be performed to verify spatial co-/anti-localization between CD38/ITGB2-high areas and CD8 exhaustion (PD-1/TOX/TIM-3) and cytotoxic markers (GZMB/IFN-γ), and to assess the impact of CD38 blockade or ITGB2 perturbation on T-cell effector function/migration.

5.Underpowered clinical inference from CheckMate 025; need for external anchoring. The exploratory re-analysis shows elevated CD38 in rhabdoid cases, but the small sample size is insufficient to support conclusions about ICI benefit. Additional immunotherapy cohorts (or real-world mini-cohorts) should be included for directional concordance; alternatively, in non-ICI public datasets (e.g., TCGA-KIRC), a composite score based on CD38/ITGB2/FOXM1/ECM can be constructed to test associations with survival and immune infiltration, thereby strengthening clinical relevance indirectly.

Referee #3 (Remarks for Author):

This manuscript describes a "Deep Visual Multi-Omics" workflow (integrating AI-guided histology, laser capture microdissection (LMD), and multi-omics) to investigate the association between morphological heterogeneity and molecular mechanisms in clear cell renal cell carcinoma (ccRCC), with a focus on aggressive rhabdoid subtypes.
The study exhibits reasonable innovation and addresses a meaningful clinical question; however, it overrelies on AI and sequencing data for validation, with insufficient experimental verification, which limits its current practical utility. The manuscript should be published after appropriate revisions.
Major Concerns:
Interference of tumor microenvironment cells (e.g., fibroblasts, immune cells) on AI classification is not mentioned, and there is no data (e.g., immunofluorescence staining) to confirm the purity of LMD-isolated "tumor cells."

Quality control data is lacking due to low cell numbers and formalin effects; while the authors have provided justifications, examples from other tissue sites could enhance the reliability of the workflow.
The study relies heavily on AI and sequencing data, with insufficient experimental validation; immunofluorescence staining of selected specimens should be performed to verify pathway activation results.
Conclusions overly depend on formalin-fixed specimens (raising concerns about reliability) and should be validated using fresh specimens, preferably supplemented with spatial transcriptomics analysis.
Minor Concern:
In independent validation, the classification accuracy of Grade 3 (68%) and Grade 4 (69%) cells is significantly lower than that of Grade 1/2 (93%) and normal renal tubules (89%), and the authors do not explain why high-grade cells have lower classification accuracy.

Point by point response to reviewer's comments
EMM-2025-22752

Referee #1 (Comments on Novelty/Model System for Author):

The manuscript, "Deep visual multiomics profiling reveals mechanisms that underlie aggressiveness in clear cell renal cell carcinoma," presents an ambitious attempt to integrate AI-based morphologic segmentation with laser-capture microdissection and transcriptomic/proteomic profiling of morphologically distinct (based on nucleolar grade) ccRCC cells. The study's combination of digital pathology and multi-omics is technically sophisticated and could provide valuable insights into histologic heterogeneity and rhabdoid differentiation.
However, the work is exploratory in scope and limited by an extremely small cohort (n=5). Its eventual use of pooled rather than true single-cell data, and lack of independent or functional validation makes the results not robust. The conclusions, particularly regarding immune evasion mechanisms and therapeutic implications are therefore descriptive and hypothesis-generating rather than mechanistically established.
While the paper demonstrates an elegant methodological framework, its scientific robustness falls short of what would be required for mechanistic or translational claims. It may best fit as a technical or methodological resource paper if reframed appropriately and supported by additional validation.

Thank you for your comments. We understand the reviewers' concerns and have carefully revised the manuscript in response. In particular, we have adjusted the wording throughout to avoid overinterpretation and to more clearly frame our findings as descriptive and hypothesis-generating. However, we would like to clarify that the central strength of this study lies not in the number of patients, but in the precision and depth of morphology-resolved molecular sampling. Across five patients with ccRCCs, we isolated and profiled approximately 40,000 individual epithelial cells selected through high-confidence AI-based classification combined with expert pathological oversight. This approach enabled the generation of transcriptomic and proteomic profiles from almost pure, morphology-defined cell populations with minimal contamination.

To our knowledge, few studies have achieved comparable morphology-specific molecular profiling of clinically relevant ccRCC cell states at this level of spatial and phenotypic resolution. Importantly, the resulting molecular signatures are consistent across cases and datasets and align well with established ccRCC biology, supporting the robustness and relevance of the observations despite the modest cohort size and associated statistical constraints.
Thus. our study establishes a framework for morphology-resolved detailed molecular profiling and, on this basis, provides initial biologically meaningful and hypothesis-generating insights into molecular programs associated with aggressive ccRCC cell states. Our design prioritizes cellular specificity over cohort breadth and allowed us to interrogate clinically relevant morphologies that are difficult to resolve using bulk or region-based approaches.

I provide some feedback that may be helpful regarding the work-

1. Four regions were selected from five ccRCC cases for whole-genome sequencing, but Figure 1B and the associated results are presented at the patient level. Please clarify how mutations and chromosomal alterations from different regions or grades within the same patient were combined. If sequencing was performed on only one region per case, this should be clearly stated, along with the corresponding grade.

We thank the reviewer for pointing out this lack of clarity. Our intention with WGS was to establish the general genomic context of each case. Thus, WGS was not performed on morphology-specific regions. Instead, for each patient, we macro-dissected tumor areas encompassing all regions that were also included in the image analysis and cell isolation workflow (grades 1-4) and combined these into one DNA sample per ccRCC case. This is illustrated in Supplementary Fig. 1B, which shows the dissected regions that were macro-dissected and pooled for genomic analysis. By doing so, we captured the overall mutational background of each ccRCC in our cohort. Consequently, the WGS results are presented at the patient level in Figure 1B and reflect the genomic landscapes for each of the ccRCCs for patients 1-5. We have clarified this in the manuscript text (Results, Methods and Figure Legens of Fig 1B).

2. Machine learning models were applied for single-cell segmentation, yet quantitative performance metrics (accuracy, precision, recall, F1 score, etc.) are not provided. These are critical to assess the discriminatory ability of the models. Moreover, given that tumor cells in ccRCC are often surrounded by stromal and immune elements and that 20 µm sections were used for cell isolation the alignment of segmented cells to flanking H&E sections may introduce spatial inaccuracies. The subsequent merging of these segmented cells for analysis effectively approximates bulk RNA-seq. Since ccRCC grade-specific clones typically form discrete clusters (as shown in Supplementary Fig. 1B), it would be helpful to compare this approach to bulk sequencing from macrodissected grade-specific regions (or punch biopsies).

We acknowledge this lack of clarity. Quantitative validation of the image analysis workflow was included in the original manuscript through cross-validation (Fig. 1F) and independent validation on unseen cases (Fig. 1G), but we agree that this was not sufficiently emphasized.

In the revised manuscript, we now present these validation results more prominently and have expanded the quantitative reporting. Specifically, we extended Fig. 1F to include the category "junk/other", which captures artefacts arising from tissue preparation staining or segmentation, as well as immune and stromal cells. For the classification task, we now explicitly report class-wise sensitivity and specificity values (Figure Legend 1F). To further support the robustness of the segmentation backbone, we included benchmarking of the nucleAIzer against several state-of-the-art models (Suppl. Table 3).

Of note, during development of this multi-class, morphology-driven workflow, we primarily evaluated performance using confusion matrices, as these provide the most interpretable representation of phenotype separation between histopathological grades and thus helped to advance our workflow. Importantly, the goal of our approach was not perfect per-cell labelling, but rather the robust identification of cells with characteristic morphological features. Our method achieves this consistently, which is also reflected in the high sensitivity and specificity values reported for each class. Moreover, this strategy aligns with routine clinical practise, where diagnostic categories such as rhabdoid differentiation are based on confident identification of characteristic cells rather than quantitative labelling of every individual cell.

Imaging, cell classification and laser microdissection were performed on the same HE-stained section, so no registration to adjacent sections was required in our workflow. This largely avoided z-plane mismatch or misalignment between image analysis and cell isolation. We added a sentence to the manuscript to highlight this.

Moreover, we now clearly describe our use of 10 µm sections. Importantly, we defined target cells by circling the nucleus and extending the contour by 6 µm to approximate the cell body. Given that ccRCC nuclear diameters are typically in the range of 10–20 µm and often larger in high-grade and rhabdoid cells (Delahunt et. al. Modern Pathology 2008, DOI: 10.1038/modpathol.2008.183) our approach enables highly enriched targeting of individual cells. High-grade and rhabdoid ccRCC cells in particular are large and cytoplasm-rich, which reduces the likelihood of isolating intermingling stromal or immune cells. Laser microdissection itself has a spatial accuracy on the order of 1–2 µm, further minimizing unintended tissue capture. In this context, we believe it is fair to say that with our approach of using a 10 µm section combined with nucleus-centred contouring minimized inclusion of adjacent stromal or immune cells. While cells were pooled for downstream RNAseq and proteomics analysis, these collections originated from highly pure, morphology-defined cells. In contrast, macrodissection or punch biopsies inevitably contain mixed stromal and immune components.

Singh et al. (https://doi.org/10.1038/modpathol.2015.68) performed paired gene expression analysis of macrodissected epithelioid and rhabdoid ccRCC components but did not identify significantly differentially expressed genes distinguishing the two morphologies. This observation motivated our

approach as it highlights the importance of analysing stringly enriched, near-pure cellular fractions to characterize molecular differences between these histological phenotypes.

3. The differential gene and protein expression (DEG and DEP) analyses are technically neat, but the small number of samples limits statistical power and the reliability of the observed trends.

We performed sample size calculations for both RNAseq and proteomics datasets. Assuming a two-sided significance level of α = 0.05 and 80% power, we estimated the number of samples required per group to detect log2 fold changes (delta) of 0.59, 1, and 2 (corresponding to 1.5X, 2X, and 4X changes).

RNAseq

(α = 0.05, power = 80%, BCV - biological coefficient of variation - at 50th percentile)

		Required Samples per group		
	**BCV**	**delta = 0.59**	**delta = 1.00**	**delta = 2.00**
Grade 1/2	0.706	48	17	5
Grade 3	0.691	46	16	4
Grade 4/rhabdoid	0.621	37	13	4

Proteomics

(α = 0.05, power = 80%, SD at 50th percentile)

		Required Samples per group		
	**SD_trimmed**	**delta = 0.59**	**delta = 1.00**	**delta = 2.00**
Grade 1/2	0.350	7	4	2
Grade 3	0.515	13	6	3
Grade 4/rhabdoid	0.555	15	6	3

Given the available sample size (N = 5 per group), the study is well powered to detect large expression changes in RNAseq and moderate-to-large changes in proteomics, whereas smaller effects may be underpowered. Accordingly, results are interpreted using a reporting threshold of log2FC ≥ 1 and within a discovery-oriented framework.

Given the rarity of rhabdoid ccRCC, particularly specimens with clearly differentiated and spatially matched G1/2, G3, and rhabdoid components within the same tumor and available research consent (see external cohort comparison in Question 12 and Reviewer 2, Question 5), this sample size represents a realistic and informative cohort for exploratory molecular characterization. We have added appropriate statements to the Methods and Discussion sections to transparently acknowledge these limitations.

4. As shown in Supplementary Figure 2, inter-patient variability appears to dominate over grade-related differences. Please specify how this confounding factor was accounted for during the analyses.

We respectfully disagree on the interpretation of Supplementary Fig. 2. While inter-patient variability is present, the samples do not cluster predominantly by patient identity. Instead, normal samples group together across patients, and ccRCC samples demonstrate visible proximity by morphological grades both for RNAseq and proteomics data. In several instances, grade-specific samples from different patients cluster more closely together than samples of different grades within the same patient. To support this notion, we additionally labeled samples by patient identifier in the PCA plots (Fig. 2A and C) and show that only samples from patient 5 exhibit proximity, while samples from patients 1-4 are distributed according to morphological phenotype rather than patient identity. Thus, although patient-specific background effects exist, grade-associated molecular structure remains clearly discernible. We have added a sentence to the relevant paragraph of the Results section to emphasize this.

In addition, inter-patient variability has been an expected phenomenon, as substantial inter-tumor heterogeneity is well-established for ccRCC (Bolck et al. Eur Urol Focus 2021, doi: 10.1016/j.euf.2019.06.009 and Turajilic et al. Cell 2018, doi: 10.1016/j.cell.2018.03.043). We carefully considered this in our study design. To minimize potential inter-patient confounding, we deliberately selected ccRCC cases that displayed all morphological phenotypes of interest side-by-side, enabling comparison of distinct morphologies within a shared patient-specific genomic and phenotypic context (as opposed to all grade 1/2 cells comeing from one patient and all rhabdoid cells from another). Of

note, this restricted the number of available specimens considerably, even with a large tissue collection at hand.

We also explored models incorporating patient identity as a factor (paired design). However, particularly in the proteomics dataset, not all grade categories were available for each patient, and a strictly paired analysis would have substantially reduced statistical power. We therefore performed the main analyses in a non-paired framework while confirming that key grade-associated molecular patterns were reproducible across tumors. Together, these observations support that the detected differences reflect consistent morphology-associated programs rather than single-patient effects.

5. Since normal controls were excluded from DEG/DEP analyses for at least two cases, please clarify how these comparisons were conducted. Were remaining normals pooled or substituted from other patients?

Differential expression analyses were performed using an unpaired group comparison design in which ccRCC grade groups were contrasted against the remaining histologically normal tubuli samples pooled across the cohort. This approach was chosen to maximize statistical power given the moderate number of samples and is supported by the PCA and hierarchical clustering analyses, which show grouping of normals and clear separation from ccRCC samples. No substitution from external patients or datasets was performed. The Methods section has been updated to clarify this.

6. Gene selection in several figures including Figure 3C: Please describe the rationale for choosing the genes displayed in Figure 3C were these based on significance, pathway enrichment, or prior biological relevance?

Gene selection in Fig. 3C (and EV Fig 3C), 5 B, D and 6C was guided by pathway enrichment analysis. For Figure 3, over-representation analyses (ORA) were performed separately for the transcriptomic and proteomic datasets. From significantly enriched pathways (e.g., oxidative phosphorylation, fatty acid metabolism, glycolysis, HIF-1 signalling), we selected representative genes that were significantly deregulated within the respective datasets and are well-established in ccRCC biology.

For Figures 5 and 6, gene selection was based on the integrated multi-omics analysis and subsequent network modelling. Candidates were first prioritized based on statistical significance, cross-layer consistency, and network properties (e.g., connectivity and centrality). We then highlighted biologically interpretable nodes with known or plausible relevance to ccRCC aggressiveness. We have clarified this rationale in the revised manuscript.

7. Have any of the transcriptomic or proteomic findings-particularly discrepant ones-been validated using IHC or another orthogonal method to distinguish technical versus biological differences?

For the validation, we did not specifically focus on discordant results from RNA and protein expression analyses, as this was not the centre of our study. However, we performed orthogonal validation of some key biologically findings using IHC. Specifically, we validated CD38, ITGB2 and CDH1 (Figure 5 E-G). These experiments confirmed the spatial expression patterns observed in our multi-omics data and support that the identified molecular programs reflect biological differences rather than technical artifacts. We have clearly described this in the revised manuscript.

Another study (Li et al.) showed elevated expression of KIF2A, NAMPT, and GALNT2 as a feature of rhabdoid cells and we can confirm these findings in our datasets (EV. Fig 3D)

8. Label is missing for Fig 3A.

We have carefully reviewed Fig. 3A but were unable to identify a missing label. We would be grateful for further clarification regarding which specific label is missing, and we will be happy to amend the figure accordingly.

9. Prior studies have demonstrated progressive dysregulation of glycolytic and oxidative phosphorylation pathways with increasing tumor grade. Since your analysis does not reproduce these patterns, it would be helpful to address potential reasons for this discrepancy in the Discussion.

Consistent with established ccRCC biology, our data recapitulate progressive metabolic rewiring with increasing grade, including repression of oxidative phosphorylation and TCA cycle activity across both molecular layers (Fig. 2E–F). While glycolysis and HIF-1 pathway enrichment are not uniformly detected at the GO-term level, glycolysis-related signatures are present in the proteomics ORA (GO:0006096; Suppl. Table 7) and become more pronounced following multi-omics integration (EV Fig. 4B). Importantly, inspection of key pathway components confirms the expected expression patterns, including induction of glycolytic enzymes (e.g., HK2, ALDOA) and HIF targets (e.g., SLC2A1, VEGFA), alongside coordinated repression of mitochondrial programs (Fig. 3C). The comparatively modest pathway-level dynamics likely reflect that these metabolic programs are already strongly deregulated in low-grade tumors due to early VHL–HIF–driven reprogramming being a hallmark of low and higher grade ccRCC.We added a statement clarifying this to the Discussion.

10. Validation of E-cadherin, CDH1, CD38, and related markers in larger datasets would strengthen the conclusions. It would be particularly valuable to determine whether expression of these markers varies between regions of different grades within the same patient.

We have extended our analysis of CDH1 (E-cadherin), CD38, ITGB2, and FOXM1 to the TCGA-KIRC and CheckMate 025 cohorts. Across these datasets, we observe a consistent direction of effect that mirrors the expression patterns identified in our morphology-resolved cohort. Of note, in TCGA-KIRC, we used grade 4 ccRCC as the closest available proxy as explicit rhabdoid annotation is not available but we acknowledge that this may category also contain other grade 4 morphologies.

Importantly, these large-scale datasets lack morphology-resolved and multi-region sampling and therefore do not permit assessment of expression differences between distinct histological regions within the same tumour. To address this, we complemented our analysis with an expanded in-house cohort, in which we validated CD38 and ITGB2 expression (Figure 5E-G) alongside the results for CDH1 in a morphology-resolved way.

11. Prognostic performance of FOXM1, ITGA4, and CDH1: In Supplementary Figures 4B and 5A/B, did these markers demonstrate superior prognostic discrimination compared with histologic grade alone?

To address this point, we performed multivariate Cox regression analyses and assessed model performance using the concordance index (C-index). Histologic grade demonstrated the highest standalone prognostic discrimination (C-index 0.668), exceeding FOXM1 (0.634), CDH1 (0.595), and ITGB2 (0.515). While inclusion of FOXM1 or CDH1 alongside grade resulted in modest improvements in model performance (C-index up to ~0.697), none of the individual gene markers outperformed histologic grade alone (see below).

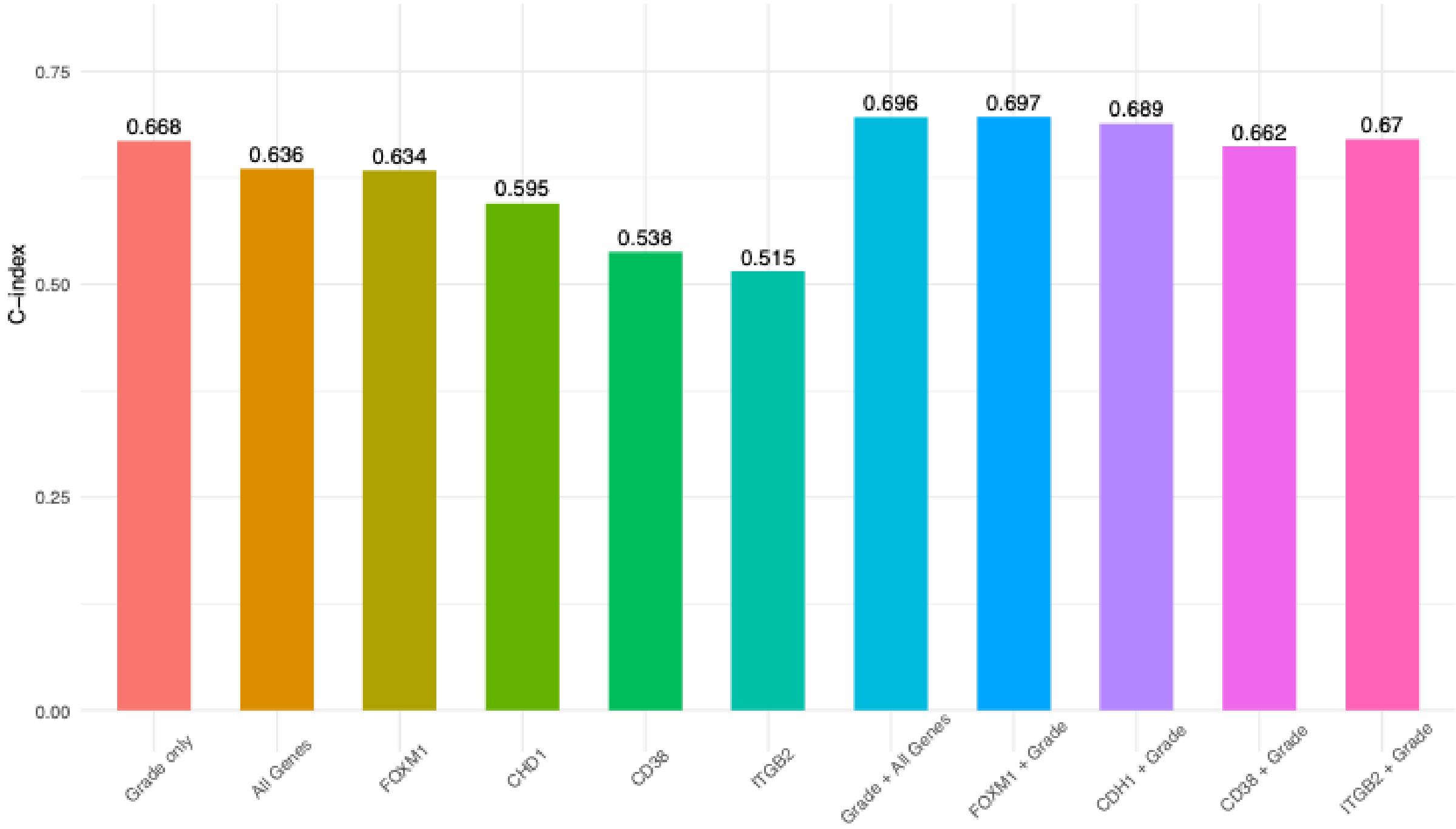

C-Index Comparison of Prognostic Models

In response to the reviewer's comment, we have further extended the survival analyses using the TCGA-KIRC cohort and now include Kaplan–Meier curves for the most relevant factors discussed by our study: FOXM1, CDH1, CD38, and ITGB2 (EV Fig. 5A). Consistent with the C-index results, FOXM1 and CDH1 showed moderate associations with overall survival, whereas CD38 and ITGB2 demonstrated weaker or no clear separation, and histologic grade provided the most pronounced stratification.

Importantly, our intention was not to propose that any of these factors would be superior prognostic markers relative to histologic grade. Rather, these factors were identified as components of molecular programs associated with higher-grade and rhabdoid ccRCC cell states. The survival analyses were included as supportive external context. The primary evidence for their importance derives from their grade-associated differential expression, network centrality, pathway context, and orthogonal validation. We have clarified this distinction in the revised manuscript.

12. The Discussion would benefit from placing these results in the context of previously published large-scale NGS studies that examined both grade and rhabdoid morphologies in ccRCC.

We thank the reviewer for this important point and have expanded the manuscript accordingly. While prior genomic studies have provided important insights into ccRCC biology, rhabdoid differentiation remains incompletely resolved in existing (large-scale) datasets:

- ***TCGA-KIRC** (Nature 2013): Rhabdoid morphology was not included as a primary stratifier and was only retrospectively annotated (in Bakouny et al., 2021); it remains unclear whether rhabdoid regions were samples for DNA analyses.*
- ***CheckMate 010/025** (Braun et al., Nat Med 2020): Whole-exome NGS data (WES) are available from 454 patients, including 18 rhabdoid cases; however, no rhabdoid-focused genomic analysis was reported.*
- ***Bakouny et al.** (Nat Commun 2021): Re-analysis of TCGA and CheckMate cohorts deliberately combines sarcomatoid and rhabdoid tumors (S/R; n=208) into one cohort and relies on single-region bulk sampling, noted by the authors as a limitation.*
- ***CPTAC** (Li et al., Cancer Cell 2023): Includes rhabdoid ccRCC as a subgroup but was not designed for rhabdoid-resolved inference; only three rhabdoid ccRCCs were profiled by WES.*
- ***Gupta et al.** (Mod Pathol 2019): analyzed 17 rhabdoid cases and reported enrichment of 9p24.1 amplification associated with PD-L1 expression.*
- *Dedicated, morphology-aware studies (**Singh et al. 2015; Lu et al. 2025**): examined similarly sized cohorts as ours (n=4–17) but with more limited molecular depth and less refined sampling of rhabdoid components.*

Consistently, prior studies have not been able to establish recurrent rhabdoid-specific genomic drivers. Primarily, they identified alterations associated with aggressive ccRCC, most reproducibly BAP1 mutations. Even morphology-aware analyses demonstrate substantial genetic overlap between the rhabdoid and epithelioid tumor component, which motivated our focus on transcriptomic and proteomic profiling as potential determinants of the rhabdoid phenotype. We have revised the Discussion to better position our findings within this context and to highlight how our morphology-resolved multi-omics approach provides additional resolution for this rare type of ccRCC.

13. The authors are encouraged to tone down claims and reframe findings as hypothesis-generating.

We revised the manuscript accordingly.

<u>**Referee #2 (Remarks for Author):**</u>

This manuscript is methodologically creative however biologically seems underpowered. It serves more as a proof-of-concept than a robust mechanistic study. The AI, laser capture and omics pipeline is neat and could be impactful if validated on larger cohorts and linked to clinical outcomes. Currently, however, conclusions about rhabdoid morphology specific immune suppression and therapeutic implications are a bit overstated relative to sample size and data depth.

We thank the reviewer for this evaluation of our work.

Referee #2 (Remarks for Author):

1.Insufficient sample size and generalizability. The principal findings are derived from five cases with morphology-defined subregions. Although the study demonstrates grade-progressive molecular dysregulation and an immunosuppressive program in rhabdoid-like cells, the small cohort and hierarchical (patient/subregion) structure may amplify random effects and limit external validity; increasing the sample size is recommended.

While the cohort size may appear limited, we wish to emphasize that we are analysing 40 000 individually selected cells. As already stated in response to similar questions by the first reviewer, we want to highlight that combining transcriptomic and proteomic profiling of only 1000 individually selected cells based on clinically relevant morphologies provide an exceptional resolution and internal consistency across the selected samples. Moreover, samples size estimation shows that our study is sufficiently powered to detect significant expression changes. Strong alignment of the results with known ccRCC tumor biology support the robustness of our conclusions. We have ensured these points are more clearly emphasized in the revised manuscript and also adjusted the tone to avoid overstatement. Moreover, we would like to note that we validated several key findings using orthogonal approaches such as IHC in a larger cohort, supporting that important findings are not driven by single cases.

2. Inadequate reproducibility and generalization of the AI pathology-guided LCM pipeline. The closed loop of "AI-driven cellular grading → LCM-directed single-cell capture → deep multi-omics" is emphasized, but details on model training/validation splits, performance metrics (AUC, sensitivity, specificity, confusion matrices), failure cases, and interpretability evidence are lacking; independent generalization on external whole-slide images (WSI) is also not reported.

We agree that the description of the AI workflow and its validation was not sufficient in the original version and have therefore substantially expanded it. We now provide a clearer description of the model development and validation strategy (Results, Methods (Image Analysis), and Suppl. Table 4). Performance metrics are reported more explicitly and include class-wise sensitivity and specificity values in addition to confusion matrices (Figure legend 1F and Suppl. Table 3). This is addressed in more detail in our response to Reviewer 1 (Question 2). We also clarified how the models retain interpretability relying on explicitly extracted morphological and contextual features derived from nuclear, cytoplasmic, and neighbourhood characteristics besides substantial expert feedback.

Prompted by the question about generalization, we additionally performed a patient-based leave-one-out (LOO) cross-validation analysis for our core cohort (N=5). This approach represents a particularly stringent validation strategy, as it tests model performance on unseen patients' ccRCC morphologies with potentially distinct patterns. LOO nevertheless yielded encouraging results:

**ccRCC**	Grade 1/2	Grade 3	Grade 4/rhabdoid
Grade 1/2	93.60%	1.74%	4.65%
Grade 3	11.88%	87.81%	0.31%
Grade 4/rhabdoid	7.14%	18.68%	74.18%

We extended the Discussion stating that the largest source of classification ambiguity occurs between grade 3 and grade 4/rhabdoid cells, which form a morphological continuum and can be challenging to distinguish even for experienced pathologists. Independent generalization to external whole-slide images was already included in the original submission (Fig. 1G), where the model was evaluated on previously unseen cases with expert-annotated ground truth.

Moreover, to facilitate reproducibility and independent evaluation of the digital pathology workflow, we provide access to the datasets and software resources used in this study (Data Availability Statement).

We note that the models were trained on a limited number of ccRCC cases (N=10; Suppl. Table 4), which inherently constrains generalization performance, particularly in a tumor type characterized by pronounced morphological heterogeneity such as ccRCC. As LOO is extremely stringent and unstable for small cohorts, in the manuscript we emphasize our primary validation strategy based on 10-fold cross-validation and evaluation on independent unseen cases. We have also added a clarifying

statement in the Discussion acknowledging this limitation and contextualizing the scope of the AI component. We perceive that in its current form, the manuscript provides a clearer and more comprehensive description of the training data, validation strategy, and applicability of the digital pathology workflow used in this study.

3.Insufficient quantification of multi-omics QC, missing-data handling, and cross-layer consistency. Separation and functional concordance between transcriptome and proteome are largely qualitative; systematic reporting of LCM cell counts, library/protein identification QC metrics, missing-value handling, and batch-effect control is absent; transcript-protein consistency lacks quantitative matrices and formal statistical testing.

In the revised manuscript, we have expanded the sections on QC metrics and procedures, particularly on missing-data handling. Specifically, we clarified this for the RNAseq analysis workflow, including read processing, alignment, gene-level quantification, and differential expression analysis using the DESeq2 framework. We also added additional information on filtering of lowly expressed genes, sequencing depth considerations for FFPE-derived total RNA libraries, and the evaluation of potential technical biases (LOESS-based bias assessment for gene length or GC content). For the proteomics layer, we expanded the description of the DIA-NN processing workflow, protein-level filtering criteria, and the statistical analysis performed using the prolfqua framework. We also clarified how missing proteomics values were handled during statistical modelling.

PCA and hierarchical clustering (Fig 2 A, C andEV Fig 1 A, B) of both datasets confirmed that samples primarily grouped according to biological features31/03/2026 18:18:00 rather than technical processing, indicating that batch effects did not drive the observed molecular patterns.

Finally, transcript–protein concordance is quantitatively assessed in Fig. 3A, where RNA–protein correlation coefficients are reported. To clarify this point further, we added references placing the observed level of concordance in the context of prior proteogenomic studies. It is well established that mRNA and protein abundances correlate only moderately, even in bulk measurements, with typical correlations explaining 30–50% of variance due to extensive post-transcriptional regulation, translational control, and protein stability mechanisms (e.g., Vogel & Marcotte doi: 10.1038/nrg3185., 2012; Liu et al., 2016 doi: 10.1016/j.cell.2016.03.014). Vogel et al explicitly state: "…the cellular concentrations of proteins correlate with the abundances of their corresponding mRNAs, but not strongly. They often show a squared Pearson correlation coefficient of ~0.40". In addition, renal cell carcinoma frequently exhibits large-scale copy-number alterations, and several proteogenomic studies have demonstrated that transcript-level changes associated with CNVs are partially buffered at the proteome level, which further diminishes transcript–protein correlations (Goncales et al. 2017 doi: 10.1016/j.cels.2017.08.013, Di et al. 2025 doi: 10.1016/j.molcel.2025.10.023). We therefore interpret the level of concordance observed in our dataset as consistent with prior proteogenomic observations with improved concordance at the pathway level, reflecting coordinated regulation of biological processes.

Together, these revisions provide a clearer description of multi-omics quality control, missing-data handling, and quantitative transcript–protein concordance in the revised manuscript.

4.Immune biology conclusions lack direct functional and spatial corroboration. The claim that rhabdoid-like tumor regions exhibit an immunosuppressive network (IFN-β, PD-L1, CD38, ITGB2) in a CD8-rich context-potentially driving T-cell exhaustion/functional suppression-relies mainly on expression, pathway, and network evidence, without functional readouts (e.g., spatial co-localization of exhaustion markers Ki67/GZMB/TOX/TIM-3/PD-1) or perturbation data. mIHC/IMC or spatial transcriptomics should be performed to verify spatial co-/anti-localization between CD38/ITGB2-high areas and CD8 exhaustion (PD-1/TOX/TIM-3) and cytotoxic markers (GZMB/IFN-γ), and to assess the impact of CD38 blockade or ITGB2 perturbation on T-cell effector function/migration.

We agree that the immune-related findings require careful interpretation. In the revised manuscript, we have therefore added orthogonal analyses and moderated mechanistic claims.

We performed IHC analyses in an extended cohort with morphology-defined regions. Particularly, in the rhabdoid areas, we observed high expression of CD38 and ITGB2 together with abundant CD8 $^{+}$ T-cell infiltration. Importantly, CD8/TOX co-staining evaluated in these same rhabdoid regions demonstrated TOX co-expression in the majority of cases, indicating spatial co-localization of CD38, ITGB2 and T cells with an exhaustion-associated phenotype. While not providing functional validation, these data offer direct morphology-matched spatial support for our observations.

We agree that approaches such as spatial transcriptomics or perturbation experiments would be valuable. However, these approaches require fresh frozen material or viable cells, whereas rhabdoid morphology can only be reliably identified in FFPE tissue based on histopathological criteria. The morphological features central to our study are not directly assessable in living cells and are not preserved in frozen sections, and no robust molecular markers currently exist to prospectively identify rhabdoid regions. As a result, it is not feasible to selectively interrogate these regions using conventional spatial transcriptomics or to isolate corresponding live cell populations for functional assays.

We have revised the manuscript to frame these findings more appropriately, while also providing an additional layer of support from our IHC analyses.

5.Underpowered clinical inference from CheckMate 025; need for external anchoring. The exploratory re-analysis shows elevated CD38 in rhabdoid cases, but the small sample size is insufficient to support conclusions about ICI benefit. Additional immunotherapy cohorts (or real-world mini-cohorts) should be included for directional concordance; alternatively, in non-ICI public datasets (e.g., TCGA-KIRC), a composite score based on CD38/ITGB2/FOXM1/ECM can be constructed to test associations with survival and immune infiltration, thereby strengthening clinical relevance indirectly.

We agree with the reviewer that the exploratory analysis of the CheckMate 025 cohort is underpowered for any conclusions regarding ICI benefit in rhabdoid ccRCC. We therefore position this analysis as supportive of the expression differences observed in our morphology-resolved cohort. In addition, we have now included additional factors associated with rhabdoid differentiation in our multi-omics data (CDH1, FOXM1, and ITGB2), which show consistent expression patterns across a larger cohort (N = 139) of rhabdoid tumors.

To address the reviewer's request for additional external anchoring, we analysed the TCGA-KIRC cohort. As rhabdoid differentiation is not explicitly annotated in this dataset, we used Grade 4 tumors as a surrogate, while acknowledging that only a subset of these cases exhibits rhabdoid features. Also in this cohort, we observed directionally consistent expression patterns, including increased FOXM1, CD38, and ITGB2 expression and decreased CDH1 expression (Fig. 6D), supporting the association of these markers with aggressive disease phenotypes.

To further investigate immune-related signals, we analysed the ESTIMATE immune score, which reflects immune cell admixture in bulk tumor samples (Yoshihara et al., 2013, doi: 10.1038/ncomms3612). Within Grade 4 tumors, higher immune scores showed a trend towards differences in overall survival; however, this did not reach statistical significance (p = 0.43), indicating that immune infiltration at the bulk level alone does not robustly stratify patient outcome, consistent with prior reports (Sobottka et al. doi.org/10.1016/j.modpat.2024.100558.). Consistently, a composite immune score based on CD38, ITGB2, and CD274 (PD-L1) did not significantly associate with overall survival (p = 0.45) and remained inferior to histologic grade in prognostic performance (see Reviewer 1, Question 11).

Figure for reviewers removed

We examined available treatment annotations in TCGA-KIRC and identified only a very small subset of Grade 4 cases with recorded ICI exposure (N = 10). Kaplan–Meier analysis in this subset is not interpretable due to the extremely limited sample size and is therefore included here only for illustration, but not in the manuscript.

While we agree that additional ICI-treated cohorts would be valuable, such datasets with both morphology-resolved annotation and RNA and/or proteomics profiling are currently extremely scarce. We have therefore added a literature overview to highlight this limitation.

Even in large-scale studies only a handful of rhabdoid ccRCC can be identified. Smaller studies that include rhabdoid cases typically rely on targeted sequencing or limited sample sizes, underscoring the lack of adequately powered cohorts for this question. Smaller studies using microdissection approaches provide higher-resolution insights but remain as limited by cohort size. Together, these observations highlight the current lack of adequately powered datasets for assessing ICI benefit in rhabdoid ccRCC.

**Study**	**Cohort**	**Rhabdoid RCC**	**Data**	**Comment**
**Large-scale Studies**				
TCGA KIRC Nature 2013	N=434 ccRCC (Bulk RNAseq data)	No information	NGS, Bulk RNAseq	No data on rhabdoid moprhology in the original design, unclear if rhabdoid areas were sampled
Braun et al. Nat Med 2021, Checkmate 025	N=261 RCC (Bulk RNAseq data)	N = 14	Bulk RNAseq, PD-L1 IHC, clincal outocmes (Everolimus vs. Nivolumab)	Rhabdoid RCC in Everolimus arm: N = 6, in Nivolumab arm: N=8, unclear how samples were processed and if rhabdoid areas were sampled
Bakouny et al., Nat Commun 2021	Re-analysis of Checkmate025 and TCGA cohorts.<br>N=1157 (Bulk RNAseq)	Not clear if all N=17 were included	Bulk RNAseq	Retrospective grading of TCGA KIRC cases revealed only 3 purely rhabdoid ccRCCs are present.
Li et al., Cancer Cell 2023	N = 213 ccRCCs	N = 3	NGS, proteogenomic and TME ITH characterization	morphology-resolved transcriptomic evidence derived from a single case
**Small-scale studies**				
Singh et al., Mod Pathol 2015	N=19 (RNAseq)	N=4	Targeted NGS, RNAseq, microarray	Morphology-resolved (macrodissection)
Lu et al. Cancers 2025	N=17	N=17	Targeted NGS	manual microdissection of samples, minimum of 20% rhabdoid tumor cell content

Finally, to provide additional external support, we examined markers reported in the CPTAC study (Li et al. Cancer Cell 2023), in which a snRNAseq subcluster with rhabdoid features (case C3N-01287) showed elevated expression of KIF2A, NAMPT, and GALNT2, a pattern supported by one additional independent rhabdoid case. We confirm consistent expression patterns of these markers in our cohort (EV Fig. 3D), further supporting the robustness of the identified transcriptional program.

While conclusions regarding ICI benefit unfortunately cannot be supported and the generation of an in-house mini-ICI cohort of rhabdoid ccRCCs with molecular profiling was not feasible during the timeframe of this revision, these analyses provide indirect support for the clinical relevance of the molecular alterations we identified for different grade morphologies of ccRCC.

<u>Referee #3 (Remarks for Author):</u>

This manuscript describes a "Deep Visual Multi-Omics" workflow (integrating AI-guided histology, laser capture microdissection (LMD), and multi-omics) to investigate the association between morphological heterogeneity and molecular mechanisms in clear cell renal cell carcinoma (ccRCC), with a focus on aggressive rhabdoid subtypes.
The study exhibits reasonable innovation and addresses a meaningful clinical question; however, it overrelies on AI and sequencing data for validation, with insufficient experimental verification, which limits its current practical utility. The manuscript should be published after appropriate revisions.

We thank the reviewer for the careful evaluation of our manuscript and for highlighting important aspects that require revision. We appreciate the overall positive assessment and address each point in detail below. Where similar concerns were raised by other reviewers, we refer to those responses to avoid redundancy and ensure consistency.

Major Concerns:

Interference of tumor microenvironment cells (e.g., fibroblasts, immune cells) on AI classification is not mentioned, and there is no data (e.g., immunofluorescence staining) to confirm the purity of LMD-isolated "tumor cells."

We would like to emphasize that morphology is the primary and most reliable diagnostic criterion in pathology, particularly for ccRCC grading and rhabdoid differentiation, and can only be assessed in HE-stained FFPE tissue. Experienced pathologists can reliably distinguish epithelial tumor cells from stromal and immune cells based on morphology alone, which represents the clinical gold standard. In this context, morphology constitutes a validated ground truth and does not require confirmation by immunofluorescence, for which no marker combination provides equivalent specificity for these phenotypes.

Moreover, in the revised manuscript, we state more clearly that AI-based classification was used to prioritize candidate cells for isolation, but final selection was based on AI-guided high-confidence ranking and additional pathological review, ensuring inclusion of only unequivocally defined cells. An “other/junk” class was explicitly incorporated during model training to capture stromal and immune components and minimize their inclusion (explained in more detail in response to Reviewer 2, Question 3). Moreover, imaging, classification, and laser microdissection were performed on the same section, enabling precise, morphology-guided isolation and further reducing contamination. Importantly, the resulting molecular profiles consistently recapitulate known ccRCC biology across both transcriptomic and proteomic layers, supporting the purity and specificity of the sampled cells.

Overall, our approach minimizes confounding from tumor microenvironment and immue components and provides a highly specific molecular characterization of grade 1/2, grade 3, and rhabdoid ccRCC cell states.

Quality control data is lacking due to low cell numbers and formalin effects; while the authors have provided justifications, examples from other tissue sites could enhance the reliability of the workflow.

We would like to refer to our detailed response to Reviewer 2 (Question 3), where we expanded the description of quality control and data robustness.

In brief, our study employs ultra-sensitive RNAseq and proteomics workflows specifically optimized for low-input FFPE material, combined with stringent filtering and exclusion of low-quality samples. We are well aware of the potential impact of formalin fixation on RNA and protein integrity.

Prior to the main study, we conducted an extensive evaluation of the relationship between sampling depth, data quality and biological representation. Specifically, we compared mRNA profiling results from

200, 500, and 1000 individually selected FFPE-derived cells. These tests showed that 1000 cells consistently yielded the most stable and biologically meaningful transcriptomic profiles, with acceptable quality control metrics. Regarding the number of cells selected for proteomics analysis we used the guidance we acquired during the RNA profiling and our previous studies in deep visual proteomics, where we thoroughly benchmarked the sensitivity as a function of cell number (Mund et al. 2022, Migh et al. 2025).

Despite technical challenges, we observed strong biological consistency across (i) independent molecular layers (RNA and protein), (ii) multiple patients, and (iii) well-established ccRCC biology (e.g., HIF signalling, metabolic rewiring). In the revised manuscript, data robustness is supported by several lines of evidence: PCA and clustering analyses (Fig. 2A, Fig. 2C; EV Fig. 2A–B) show clear separation of normal and tumor cells and consistent grouping by morphological grade across patients, indicating that biological signal exceeds technical noise. Differential expression analyses (Fig. 2B,D) reveal increasing numbers of deregulated genes and proteins with tumor grade, and cross-layer comparisons (Fig. 3A,B) demonstrate concordance between transcriptomic and proteomic data.

Regarding the reviewer's suggestion of examples from other tissue sites, we have previously performed a proof-of-concept analysis in a mixed-tissue context, where a ccRCC specimen was infiltrated by metastatic breast cancer cells. Our approach accurately distinguished ccRCC and breast cancer expression patterns (Figure below). This supports the broader robustness and applicability of the workflow.

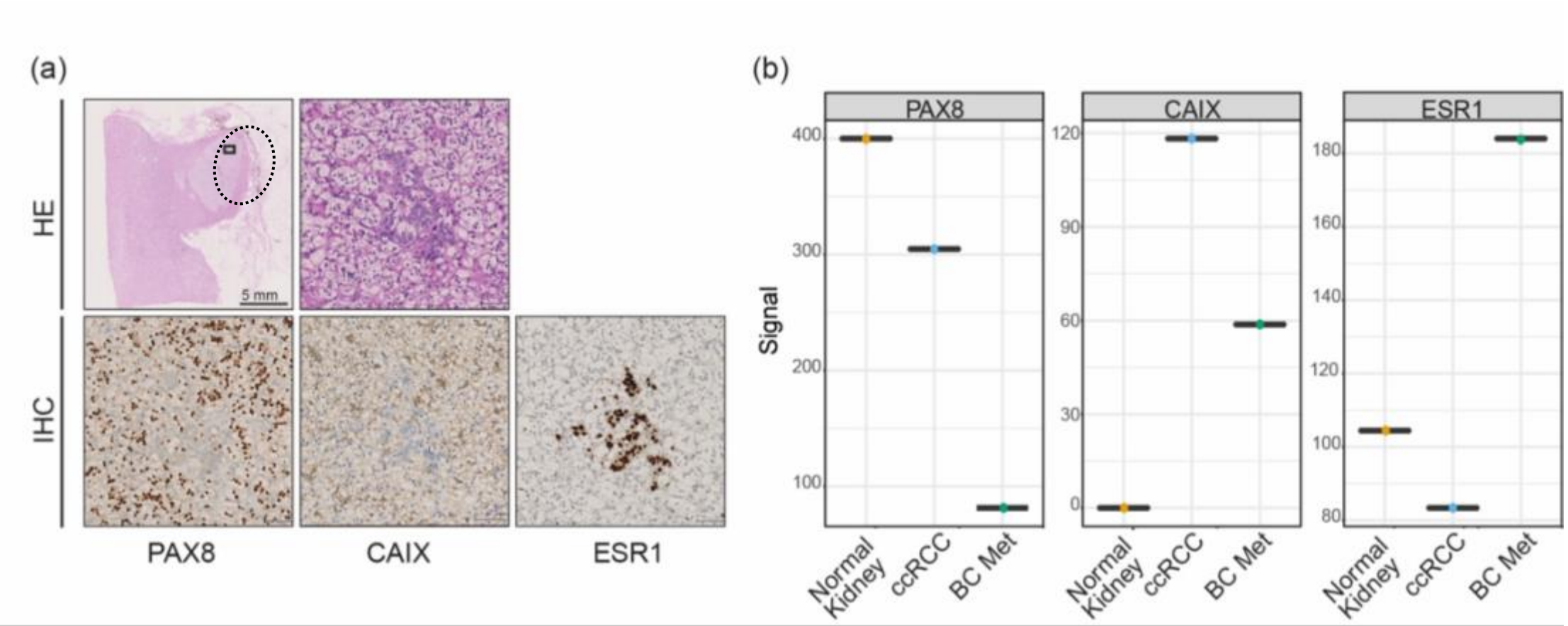

Assay Development Figure: *(a) HE staining depicting the presence of a breast cancer metastasis (black square) within a ccRCC (dotted line) as part of the kidney (whole section). The region in the black square is magnified and shows expression of breast cancer marker ESR1, the ccRCC marker CAIX and the kidney-specific protein Pax8. (b) Protein expression shown in (a) is reflected on the transcriptional level in individually excised cells from the corresponding regions by RNAseq. BC Met – breast cancer metastasis*

While cross-tissue benchmarking was not the primary aim of this study, we have now clarified this aspect.

The study relies heavily on AI and sequencing data, with insufficient experimental validation; immunofluorescence staining of selected specimens should be performed to verify pathway activation results. Conclusions overly depend on formalin-fixed specimens (raising concerns about reliability) and should be validated using fresh specimens, preferably supplemented with spatial transcriptomics analysis.

As detailed in our response to Reviewer 2, Comment 4, we have expanded experimental validation and adjusted the tone of our conclusions. Briefly, we performed IHC analyses in an extended cohort with morphology-defined regions, confirming regulation patterns of CD38, ITGB2, and CDH1. In addition, CD8/TOX co-staining demonstrated spatial co-localization of $CD8^+$ T cells with the exhaustion-associated marker TOX in rhabdoid areas, providing spatially resolved evidence that complements the expression-based analyses.

In addition, we provide independent support from external datasets, where key factors identified in our study (CD38, ITGB2, CDH1, FOXM1) show concordant dysregulation in rhabdoid cases in the

CheckMate 025 cohort and in high-grade tumors in TCGA, supporting the relevance of these observations despite their lack of morphological resolution.

As discussed above, our approach relies on histopathological identification of ccRCC cells with different grade morphology, such as rhabdoid differentiation, which can only be reliably assessed in FFPE tissue. These characteristic morphologies are not preserved in fresh frozen samples or viable cells, and no robust molecular markers exist to identify the corresponding cells in these specimens. This limits the applicability of immunofluorescence, spatial transcriptomics, or functional perturbation experiments. Moreover, potential artefacts associated with FFPE-derived material are well characterized, and such approaches are widely established for transcriptomic and proteomic analyses (for reference: link, Tüshaus et al, 2024, https://doi.org/10.1038/s44318-024-00289-w). Importantly, the integration of complementary RNAseq and proteomics data in our study provides an extra layer of substantiation.

Minor Concern:
In independent validation, the classification accuracy of Grade 3 (68%) and Grade 4 (69%) cells is significantly lower than that of Grade 1/2 (93%) and normal renal tubules (89%), and the authors do not explain why high-grade cells have lower classification accuracy.

We thank the reviewer for this observation and agree that this point was not sufficiently explained. The lower classification accuracy for Grade 3 and Grade 4 cells is mainly due to the biological continuum and morphological overlap between these high-grade phenotypes, which makes their separation inherently more challenging than distinguishing low-grade ccRCC cells. We have explained this better in the revised manuscript.

25th May 2026

Dear Dr. Horvath,

Thank you for submitting your revised manuscript, and please accept my apologies for the delay in getting back to you, as one referee needed more time to provide a report.
Referees #1 and #2 have now evaluated the revised version of your study. As you will see below, the referees are overall satisfied with the revisions, and I will therefore be able to accept your manuscript once the following points have been addressed:

1/ Referees' concerns:
Please address the remaining concerns from referee 1 in the abstract and introduction.

2/ Manuscript text:
- We think your manuscript would be better suited as a Resource and have changed the article type accordingly. No additional change is required from your side.
- Please remove the highlighted text and only keep in track changes mode any new modification.
- The manuscript sections should be in the following order: Title page - Abstract & Keywords - Introduction - Results - Discussion - Methods - Data Availability - Acknowledgments - Disclosure Statement & Competing Interests - References - Figure Legends - (Main Tables with legends if applicable) - Expanded View Figure Legends.
- Methods: Patient samples: Include a statement confirming that informed consent was obtained from all subjects and that the experiments conformed to the principles set out in the WMA Declaration of Helsinki and the Department of Health and Human Services Belmont Report.
- Data Availability: we only need one section and one title: Data Availability Statement. Sections such as Resource availability, Lead contact, Materials availability, etc. should be removed. Please note that this section is restricted to new primary data or code that are part of this study. Furthermore, according to the journal's data policy, if practically possible and compatible with the individual consent agreement, authors must deposit the human clinical datasets to public databases (for instance in EGA). Please see our guidelines at https://link.springer.com/partners/embo-press/editorial-policies#Data%20availability%20statement.
- A "Disclosure and Competing Interests Statement" should be provided in the main text. Employment in a biotech company should be stated in DCIS.
- Funding information: funders provided in the Comments box need to be removed and each should be provided as a separate entry in the system.
- References should be corrected to alphabetical order, with 10 author names listed before et al. and DOIs for published articles removed.
- Our journal encourages inclusion of *data citations in the reference list* to directly cite datasets that were re-used and obtained from public databases. Data citations in the article text are distinct from normal bibliographical citations and should directly link to the database records from which the data can be accessed. In the main text, data citations are formatted as follows: "Data ref: Smith et al, 2001" or "Data ref: NCBI Sequence Read Archive PRJNA342805, 2017". In the Reference list, data citations must be labeled with "[DATASET]". A data reference must provide the database name, accession number/identifiers and a resolvable link to the landing page from which the data can be accessed at the end of the reference.

3/ Figures:
- The nomenclature of EV figures should be changed to Figure EV1, etc. instead of Expanded View Figure 1, etc.
- Please make sure that all figures and figure panels are referenced in the manuscript text. Currently, a callout is missing for Fig. 5FG. The following should be corrected: Suppl Fig. 1; EV Tables 4 and 8, EV Table 9, EV Table 8.
- Supplementary Table 1-9 and Supplementary Table 10 should be made Dataset EV1 and Dataset EV2; the legend of Supplementary Table 10 should be provided in the Dataset EV2 file, as a separate sheet/tab.
- The Figure EV1B legend should be more detailed, as all images are reused in other figures. Figure re-use must be mentioned.
- Please address the queries from our data editors in the figure legends:
- Please note that the exact p values are not provided in the legend of figure 6D
- Please indicate the statistical test used for data analysis in the legends of figures 2B, D
- Please note that the box plots need to be defined in terms of minima, maxima, centre, bounds of box and whiskers, and percentile in the legends of figures 6D
- Please note that information related to n is missing in the legends of figures 2B, D

4/ Checklist: please fill in the section "Sample definition and in-laboratory replication".

5/ Provide "The paper explained": EMBO Molecular Medicine articles are accompanied by a summary of the articles to emphasize the major findings in the paper and their medical implications for the non-specialist reader. Please provide a draft summary of your article highlighting
- the medical issue you are addressing,

- the results obtained and
- their clinical impact.

This may be edited to ensure that readers understand the significance and context of the research. Please refer to any of our published articles for an example.

6/ Every published paper includes a 'Synopsis' to further enhance discoverability. Synopses are displayed on the journal webpage and are freely accessible to all readers. They include a short stand first (maximum of 300 characters, including space) as well as 2-5 one-sentences bullet points that summarizes the paper. Please write the bullet points to summarize the key NEW findings. They should be designed to be complementary to the abstract - i.e. not repeat the same text. We encourage inclusion of key acronyms and quantitative information (maximum of 30 words / bullet point). Please use the passive voice. Please attach these in a separate file or send them by email, we will incorporate them accordingly.

Please also suggest a striking image or visual abstract to illustrate your article as a PNG file 550 px wide x 300 px high.

7/ As part of the EMBO Publications transparent editorial process initiative (see our Editorial at http://embomolmed.embopress.org/content/2/9/329), EMBO Molecular Medicine will publish online a Review Process File (RPF) to accompany accepted manuscripts.

This file will be published in conjunction with your paper and will include the anonymous referee reports, your point-by-point response and all pertinent correspondence relating to the manuscript. Let us know whether you agree with the publication of the RPF and as here, if you want to remove or not any figures from it prior to publication.

Please note that the Authors checklist will be published at the end of the RPF.

I look forward to receiving your revised manuscript.

Yours sincerely,

Lise Roth

Lise Roth, PhD
Senior Editor
EMBO Molecular Medicine

***** Reviewer's comments *****

Referee #1 (Comments on Novelty/Model System for Author):

The authors have substantially addressed my prior concerns. The revised manuscript is more transparent regarding patient-level WGS pooling, AI model performance, sample size limitations, normal-control handling, gene-selection rationale, and the exploratory nature of the findings. The addition of morphology-resolved IHC validation and external dataset comparisons strengthens the work. I continue to view the study as primarily a technically innovative, hypothesis-generating resource rather than a definitive mechanistic study. Before acceptance, I recommend further softening of the abstract and introduction to avoid implying functional immune suppression, ICI-response prediction, or therapeutic actionability beyond the current data.

Referee #2 (Comments on Novelty/Model System for Author):

The manuscript has been improved considerably in revision, and most of my previous concerns have been addressed to a reasonable extent.

The authors have clarified the AI-based workflow, added validation details, and substantially moderated several of the original claims. I particularly appreciate that the discussion is now more balanced regarding the limited cohort size and the absence of direct functional validation. While not every point has been fully resolved experimentally, I think the revised version is much more rigorous and appropriately framed.

Overall, this is an interesting study with clear technical value, and I support publication in its present form.

The authors addressed the remaining editorial issues.

1st Jul 2026

Dear Dr. Horvath,

Thank you for sending your revised files. I am pleased to inform you that your manuscript is accepted for publication and is now being sent to our publisher to be included in the next available issue of EMBO Molecular Medicine.
Please note that all deposited data must be accessible before online publication of your manuscript.

Your manuscript will be processed for publication by EMBO Press. It will be copy edited and you will receive page proofs prior to publication. Please note that you will be contacted by Springer Nature Author Services to complete licensing and payment information.

You may qualify for financial assistance for your publication charges - either via a Springer Nature fully open access agreement or an EMBO initiative. Check your eligibility: https://link.springer.com/journal/44321/how-to-publish-with-us

Should you be planning a Press Release on your article, please notify the production team via this form in order to coordinate publication and release dates: https://forms.cloud.microsoft/e/eH4A8JVP6g

If you have any questions, please do not hesitate to contact the Editorial Office. Thank you for your contribution to EMBO Molecular Medicine.

Yours sincerely,

Lise Roth

Lise Roth, Ph.D
Senior Editor
EMBO Molecular Medicine

>>> Please note that it is EMBO Molecular Medicine policy for the transcript of the editorial process (containing referee reports and your response letter) to be published as an online supplement to each paper. If you do NOT want this, you will need to inform the Editorial Office via email immediately. More information is available here: https://link.springer.com/partners/embo-press/editorial-policies#Peer%20review

EMBO Press Author Checklist

Corresponding Author Name: Peter Horvath, Hella Bolck, Holger Moch
Journal Submitted to: EMBO Molecular Medicine
Manuscript Number: EMM-2025-22752

USEFUL LINKS FOR COMPLETING THIS FORM
The EMBO Journal - Author Guidelines
EMBO Reports - Author Guidelines
Molecular Systems Biology - Author Guidelines
EMBO Molecular Medicine - Author Guidelines

Reporting Checklist for Life Science Articles (updated January
This checklist is adapted from Materials Design Analysis Reporting (MDAR) Checklist for Authors. MDAR establishes a minimum set of requirements in transparent reporting in the life sciences (see Statement of Task: 10.31222/osf.io/9sm4x). Please follow the journal's guidelines in preparing your
Please note that a copy of this checklist will be published alongside your article.

Abridged guidelines for figures
1. Data
The data shown in figures should satisfy the following conditions:
- the data were obtained and processed according to the field's best practice and are presented to reflect the results of the experiments in an accurate and unbiased manner.
- ideally, figure panels should include only measurements that are directly comparable to each other and obtained with the same assay.
- plots include clearly labeled error bars for independent experiments and sample sizes. Unless justified, error bars should not be shown for technical
- if n<5, the individual data points from each experiment should be plotted. Any statistical test employed should be justified.
- Source Data should be included to report the data underlying figures according to the guidelines set out in the authorship guidelines on Data

2. Captions
Each figure caption should contain the following information, for each panel where they are relevant:
- a specification of the experimental system investigated (eg cell line, species name).
- the assay(s) and method(s) used to carry out the reported observations and measurements.
- an explicit mention of the biological and chemical entity(ies) that are being measured.
- an explicit mention of the biological and chemical entity(ies) that are altered/varied/perturbed in a controlled manner.
- the exact sample size (n) for each experimental group/condition, given as a number, not a range;
- a description of the sample collection allowing the reader to understand whether the samples represent technical or biological replicates (including how many animals, litters, cultures, etc.).
- a statement of how many times the experiment shown was independently replicated in the laboratory.
- definitions of statistical methods and measures:
  - common tests, such as t-test (please specify whether paired vs. unpaired), simple χ2 tests, Wilcoxon and Mann-Whitney tests, can be unambiguously identified by name only, but more complex techniques should be described in the methods section;
  - are tests one-sided or two-sided?
  - are there adjustments for multiple comparisons?
  - exact statistical test results, e.g., P values = x but not P values < x;
  - definition of 'center values' as median or average;
  - definition of error bars as s.d. or s.e.m.

Please complete ALL of the questions below.
Select "Not Applicable" only when the requested information is not relevant for your study.

Materials

Newly Created Materials	Information included in the manuscript?	In which section is the information available? (Reagents and Tools Table, Materials and Methods, Figures, Data Availability Section)
New materials and reagents need to be available; do any restrictions apply?	Not Applicable	

Antibodies	Information included in the manuscript?	In which section is the information available? (Reagents and Tools Table, Materials and Methods, Figures, Data Availability Section)
For **antibodies** provide the following information:<br>- Commercial antibodies: RRID (if possible) or supplier name, catalogue number and or/clone number<br>- Non-commercial: RRID or citation	Yes	Reagents and Tools Table, Materials and Methods

DNA and RNA sequences	Information included in the manuscript?	In which section is the information available? (Reagents and Tools Table, Materials and Methods, Figures, Data Availability Section)
**Short novel DNA or RNA including primers, probes**: provide the sequences.	Not Applicable	

Cell materials	Information included in the manuscript?	In which section is the information available? (Reagents and Tools Table, Materials and Methods, Figures, Data Availability Section)
**Cell lines:** Provide species information, strain. Provide accession number in repository **OR** supplier name, catalog number, clone number, and/**OR** RRID.	Not Applicable	
**Primary cultures:** Provide species, strain, sex of origin, genetic modification status.	Not Applicable	
Report if the cell lines were recently **authenticated** (e.g., by STR profiling) and tested for mycoplasma contamination.	Not Applicable	

Experimental animals	Information included in the manuscript?	In which section is the information available? (Reagents and Tools Table, Materials and Methods, Figures, Data Availability Section)
**Laboratory animals or Model organisms:** Provide species, strain, sex, age, genetic modification status. Provide accession number in repository **OR** supplier name, catalog number, clone number, **OR** RRID.	Not Applicable	
**Animal observed in or captured from the field:** Provide species, sex, and age where possible.	Not Applicable	
Please detail **housing and husbandry conditions**.	Not Applicable	

Plants and microbes	Information included in the manuscript?	In which section is the information available? (Reagents and Tools Table, Materials and Methods, Figures, Data Availability Section)
**Plants:** provide species and strain, ecotype and cultivar where relevant, unique accession number if available, and source (including location for collected wild specimens).	Not Applicable	
**Microbes:** provide species and strain, unique accession number if available, and source.	Not Applicable	

Human research participants	Information included in the manuscript?	In which section is the information available? (Reagents and Tools Table, Materials and Methods, Figures, Data Availability Section)
If collected and within the bounds of privacy constraints report on age, sex and gender or ethnicity for all study participants.	Yes	Table 1 and Suppl. Table 1, Ethnicities unknown.

Core facilities	Information included in the manuscript?	In which section is the information available? (Reagents and Tools Table, Materials and Methods, Figures, Data Availability Section)
If your work benefited from core facilities, was their service mentioned in the acknowledgments section?	Yes	Acknowledgement section and author list.

Design

Study protocol	Information included in the manuscript?	In which section is the information available? (Reagents and Tools Table, Materials and Methods, Figures, Data Availability Section)
If study protocol has been **pre-registered, provide DOI in the manuscript**. For clinical trials, provide the trial registration number **OR** cite DOI.	Not Applicable	
Report the **clinical trial registration number** (at ClinicalTrials.gov or equivalent), where applicable.	Not Applicable	

Laboratory protocol	Information included in the manuscript?	In which section is the information available? (Reagents and Tools Table, Materials and Methods, Figures, Data Availability Section)
Provide DOI OR other citation details if **external detailed step-by-step protocols** are available.	Yes	Material and Methods, References

Experimental study design and statistics	Information included in the manuscript?	In which section is the information available? (Reagents and Tools Table, Materials and Methods, Figures, Data Availability Section)
Include a statement about **sample size** estimate even if no statistical methods were used.	Yes	Methods
Were any steps taken to minimize the effects of subjective bias when allocating animals/samples to treatment (e.g. **randomization procedure**)? If yes, have they been described?	Not Applicable	
Include a statement about **blinding** even if no blinding was done.	Not Applicable	
Describe **inclusion/exclusion criteria** if samples or animals were excluded from the analysis. Were the criteria pre-established?<br><br>If sample or data points were omitted from analysis, report if this was due to attrition or intentional exclusion and provide justification.	Not Applicable	
For every figure, are **statistical tests** justified as appropriate? Do the data meet the assumptions of the tests (e.g., normal distribution)? Describe any methods used to assess it. Is there an estimate of variation within each group of data? Is the variance similar between the groups that are being statistically compared?	Yes	Figures, Figure legends and Material and Methods

Sample definition and in-laboratory replication	Information included in the manuscript?	In which section is the information available? (Reagents and Tools Table, Materials and Methods, Figures, Data Availability Section)
In the figure legends: state number of times the experiment was **replicated** in laboratory.	Yes	Figure legends
In the figure legends: define whether data describe **technical or biological replicates**.	Yes	Figure legends

Ethics

Ethics	Information included in the manuscript?	In which section is the information available? (Reagents and Tools Table, Materials and Methods, Figures, Data Availability Section)
Studies involving **human participants**: State details of **authority granting ethics approval** (IRB or equivalent committee(s), provide reference number for approval.	Yes	Material and Methods
Studies involving **human participants**: Include a statement confirming that **informed consent** was obtained from all subjects and that the experiments conformed to the principles set out in the WMA Declaration of Helsinki and the Department of Health and Human Services Belmont Report.	Yes	Material and Methods
Studies involving **human participants: For** publication of **patient photos**, include a statement confirming that consent to publish was obtained.	Not Applicable	
Studies involving experimental **animals**: State details of **authority granting ethics approval** (IRB or equivalent committee(s), provide reference number for approval. Include a statement of compliance with ethical regulations.	Not Applicable	
Studies involving **specimen and field samples:** State if relevant **permits** obtained, provide details of authority approving study; if none were required, explain why.	Not Applicable	

Dual Use Research of Concern (DURC)	Information included in the manuscript?	In which section is the information available? (Reagents and Tools Table, Materials and Methods, Figures, Data Availability Section)
Could your study fall under dual use research restrictions? Please check biosecurity documents and list of **select agents and toxins** (CDC): https://www.selectagents.gov/sat/list.htm	Not Applicable	
If you used a select agent, is the security level of the lab appropriate and reported in the manuscript?	Not Applicable	
If a study is subject to dual use research of concern regulations, is the name of the **authority granting approval and reference number** for the regulatory approval provided in the manuscript?	Not Applicable	

Reporting

The MDAR framework recommends adoption of discipline-specific guidelines, established and endorsed through community initiatives. Journals have their own policy about requiring specific guidelines and recommendations to complement MDAR.

Adherence to community standards	Information included in the manuscript?	In which section is the information available? (Reagents and Tools Table, Materials and Methods, Figures, Data Availability Section)
State if relevant guidelines or checklists (e.g., **ICMJE, MIBBI, ARRIVE, PRISMA**) have been followed or provided.	Not Applicable	
For **tumor marker prognostic studies,** we recommend that you follow the **REMARK** reporting guidelines (see link list at top right). See author guidelines, under 'Reporting Guidelines'. Please confirm you have followed these guidelines.	Not Applicable	
For **phase II and III randomized controlled trials**, please refer to the **CONSORT** flow diagram (see link list at top right) and submit the CONSORT checklist (see link list at top right) with your submission. See author guidelines, under 'Reporting Guidelines'. Please confirm you have submitted this list.	Not Applicable	

Data Availability

Data availability	Information included in the manuscript?	In which section is the information available? (Reagents and Tools Table, Materials and Methods, Figures, Data Availability Section)
Have **primary datasets** been deposited according to the journal's guidelines (see 'Data Deposition' section) and the respective accession numbers provided in the Data Availability Section?	Yes	Data and Code Availibility Statement
Were **human clinical and genomic datasets** deposited in a public access-controlled repository in accordance to ethical obligations to the patients and to the applicable consent agreement?	Yes	Data and Code Availibility Statement
Are **computational models** that are central and integral to a study available without restrictions in a machine-readable form? Were the relevant accession numbers or links provided?	Yes	Data and Code Availibility Statement
If publicly available data were reused, provide the respective **data citations in the reference list**.	Yes	Reagents and Tools Table, Materials and Methods
