# Supplementary material for: Deep visual multi-omics profiling links morphology and molecular programs in clear cell renal cell carcinoma
Source: EMBO Mol Med. 2026 Aug 6;18(9):3850–77. doi: 10.1038/s44321-026-00484-8 (PMC13562643; doi:10.1038/s44321-026-00484-8)
Supplement: Supplementary file 2 [file 44321_2026_484_MOESM4_ESM.pdf]

Expanded View Figures

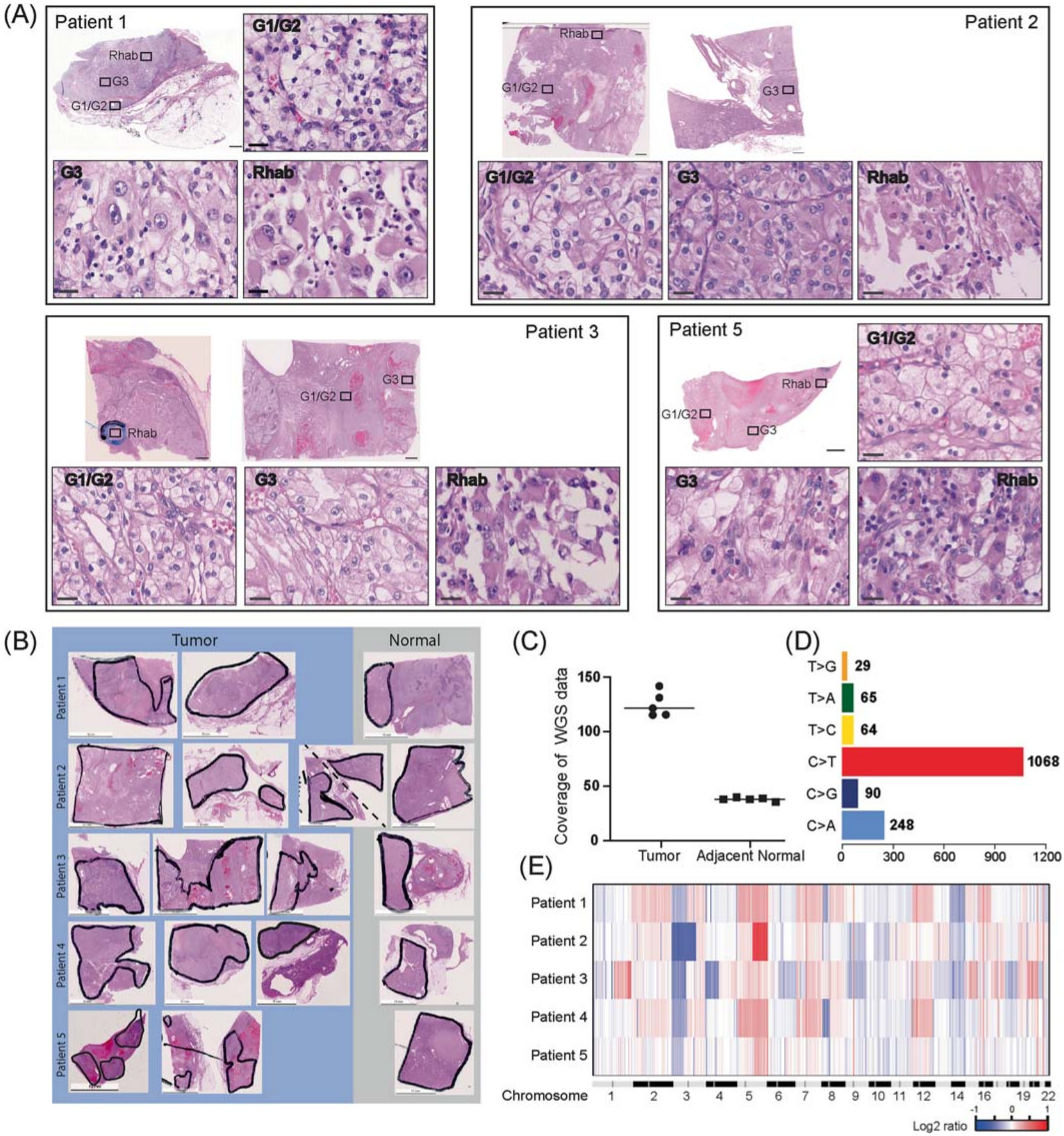

Figure EV1. Image-guided single-cell isolation and multi-omics analysis of ccRCC with pronounced morphological heterogeneity and rhabdoid de-differentiation.

(**A**) Representative H&E images indicating ITH of grade features: Grade 1/2 (low-grade, G1/2), grade 3 (high-grade, G3), and grade 4 ccRCC with rhabdoid differentiation (Rhab). Scale bars: WSI—2 mm, high magnification panels—100 μm. (**B**) Overview WSIs indicating the areas selected for dissection and subsequent WGS. The displayed sections were specifically prepared for region annotation on slides directly adjacent to those used for tissue dissection and WGS and are therefore distinct from the representative H&E sections shown in (**A**) and Fig. 1A. (**C**) Mean coverage of ccRCC and adjacent normal kidney in WGS data for all five cases. (**D**) Whole-genome mutation spectra indicating SNV classes, and their frequency, in our data, highlighting the presence of C > T transitions caused by cytosine deamination from formalin fixation of the tissue samples. (**E**) CNV profiles determined from WGS data. CNV loss/gain is indicated by color coding, blue indicates loss and red indicates gain.

© The Author(s)

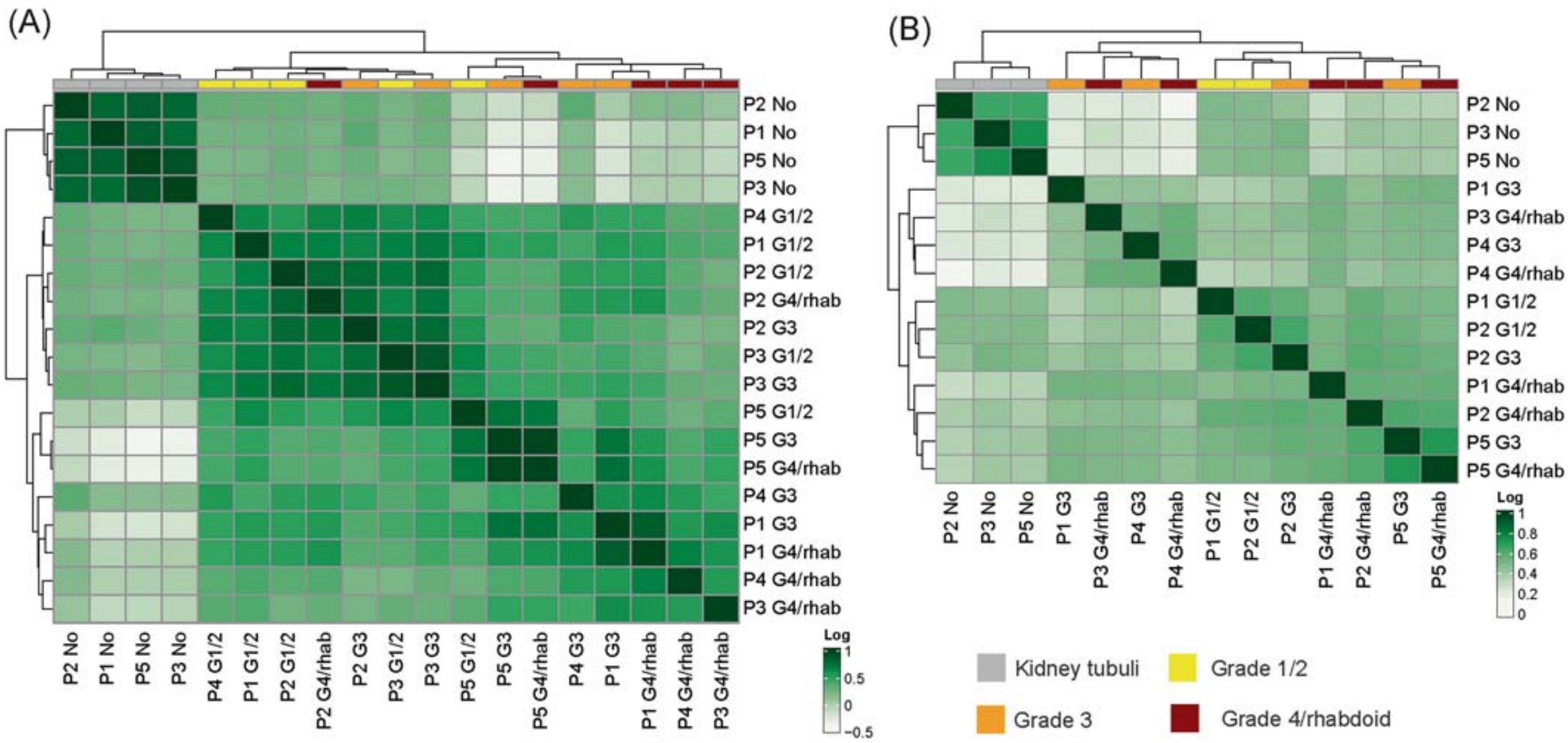

Figure EV2. Analysis of mRNA and protein expression from individually selected cells with different grade morphologies.

(**A**) Correlation analysis for top100 variance features of the transcriptomic dataset obtained from interrogating cells isolated from normal kidney tubuli tissue or ccRCC with distinct grade morphologies ($N = 19$). Correlation coefficients are indicated by the color code. (**B**) Correlation analysis for proteomics samples isolated from normal kidney tubuli tissue or ccRCC with distinct grade morphologies ($N = 14$). Correlation coefficients are indicated by the color code.

 © The Author(s)

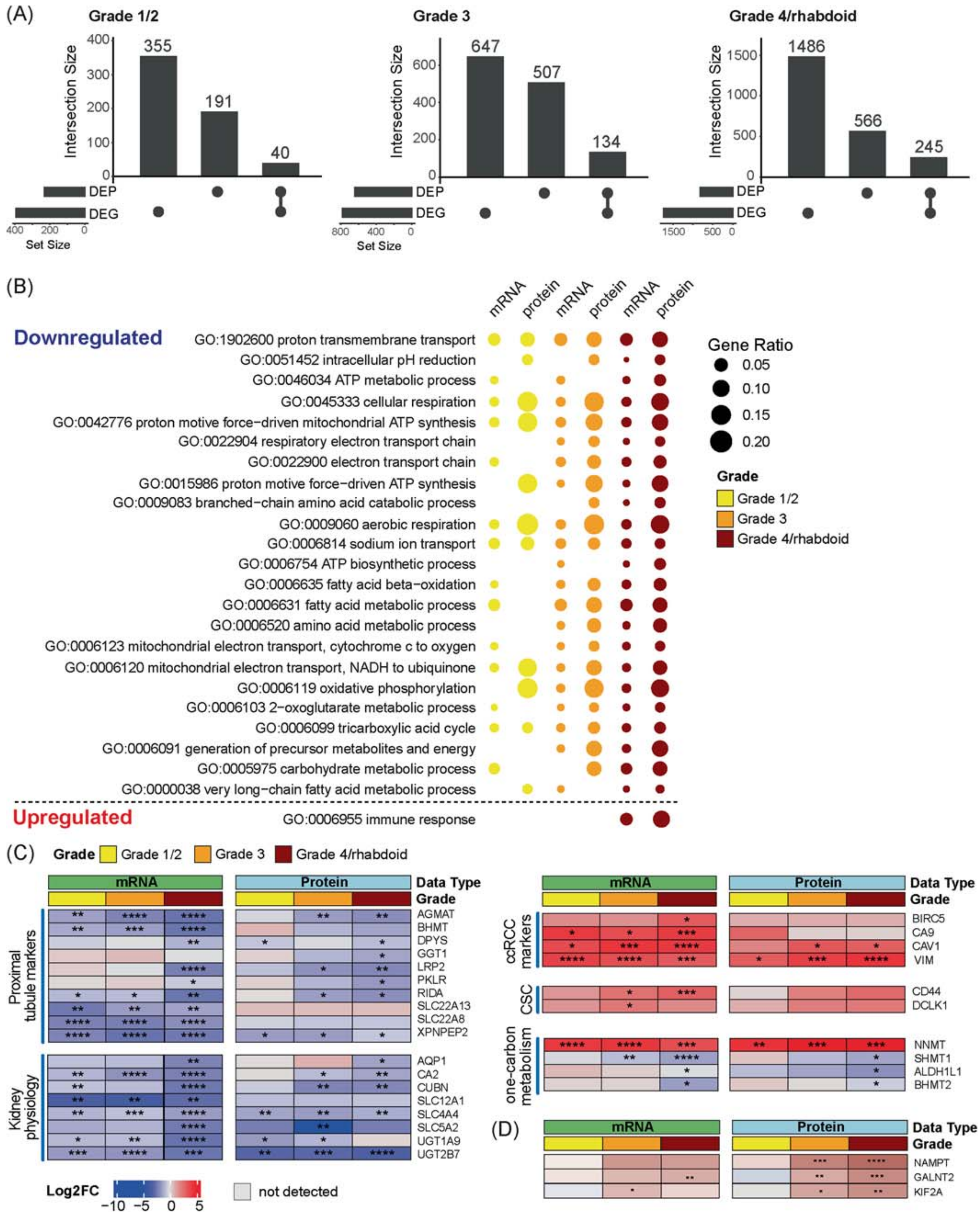

(A)
Grade 1/2
Grade 3
Grade 4/rhabdoid
Intersection Size
355
191
40
647
507
134
1486
566
245
DEP
DEG
Set Size
(B)
mRNA
protein
Downregulated
GO:1902600 proton transmembrane transport
GO:0051452 intracellular pH reduction
GO:0046034 ATP metabolic process
GO:0045333 cellular respiration
GO:0042776 proton motive force−driven mitochondrial ATP synthesis
GO:0022904 respiratory electron transport chain
GO:0022900 electron transport chain
GO:0015986 proton motive force−driven ATP synthesis
GO:0009083 branched−chain amino acid catabolic process
GO:0009060 aerobic respiration
GO:0006814 sodium ion transport
GO:0006754 ATP biosynthetic process
GO:0006635 fatty acid beta−oxidation
GO:0006631 fatty acid metabolic process
GO:0006520 amino acid metabolic process
GO:0006123 mitochondrial electron transport, cytochrome c to oxygen
GO:0006120 mitochondrial electron transport, NADH to ubiquinone
GO:0006119 oxidative phosphorylation
GO:0006103 2−oxoglutarate metabolic process
GO:0006099 tricarboxylic acid cycle
GO:0006091 generation of precursor metabolites and energy
GO:0005975 carbohydrate metabolic process
GO:0000038 very long−chain fatty acid metabolic process
Upregulated
GO:0006955 immune response
Gene Ratio
0.05
0.10
0.15
0.20
Grade
Grade 1/2
Grade 3
Grade 4/rhabdoid
(C)
Grade
Grade 1/2
Grade 3
Grade 4/rhabdoid
mRNA
Protein
Data Type
Grade
Proximal tubule markers
AGMAT
BHMT
DPYS
GGT1
LRP2
PKLR
RIDA
SLC22A13
SLC22A8
XPNPEP2
Kidney physiology
AQP1
CA2
CUBN
SLC12A1
SLC4A4
SLC5A2
UGT1A9
UGT2B7
ccRCC markers
BIRC5
CA9
CAV1
VIM
CSC
CD44
DCLK1
one-carbon metabolism
NNMT
SHMT1
ALDH1L1
BHMT2
Log2FC
−10 −5 0 5
not detected
(D)
mRNA
Protein
Data Type
Grade
NAMPT
GALNT2
KIF2A

Figure EV3. Functional overlap between mRNA and protein data.

(**A**) Upset plots indicating the number of significant for mRNA and proteomics datasets for each grade group and their overlap. (**B**) Bubble plot depicting all 24 shared GO terms for mRNA and protein differential expression in the grade 4/rhabdoid class of ccRCC cells and their overlap with samples from other grade morphologies. (**C**) Heatmaps depicting selected alterations that mark kidney cell identity (proximal tubule cells markers and factors implicated in physiological kidney functions) as well as common ccRCC markers, cancer stem cell markers, and factors implicated in one-carbon metabolism. Representative genes and proteins are depicted as log2FC with respect to normal cells. FDR is indicated by “****“ <= 0.0001, “***“ < = 0.001, “**“< = 0.01, “*“ < = 0.05. (**D**) Heatmaps showing expression of KIF2A, NAMPT, and GALNT2 are consistent with previously reported signatures. Genes and proteins are depicted as log2FC with respect to normal cells. FDR is indicated by “****“ < = 0.0001, “***“ < = 0.001, “**“< = 0.01, “*“ < = 0.05.

 © The Author(s)

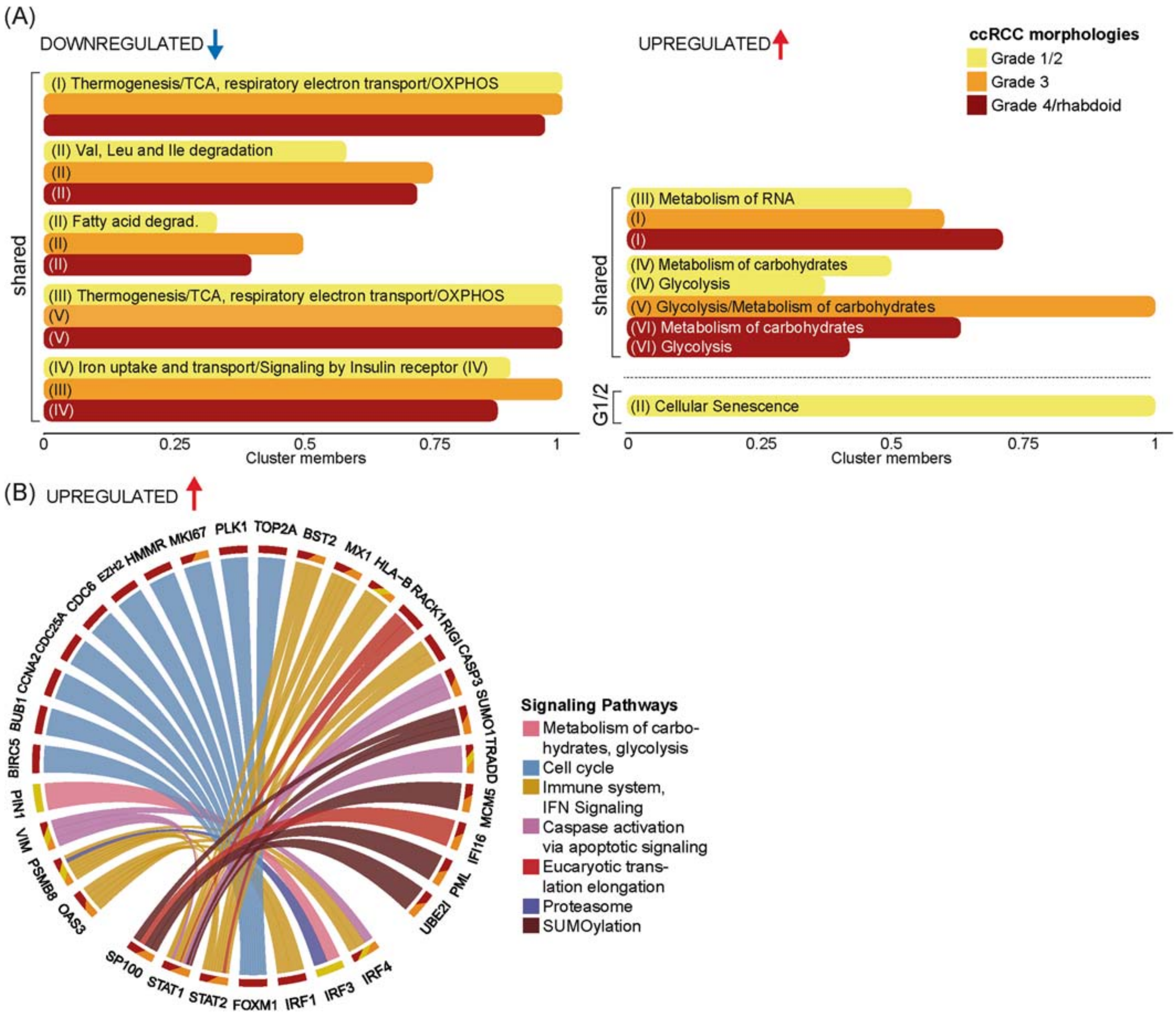

Figure EV4. Multi-omics data integration uncovers distinct deregulated processes in aggressive cells and their transcriptional regulation.

(**A**) Biological processes enriched in modules shared by Grade 1/2 ccRCC grade morphology with at least ten members following decomposition of the PPI networks in (**A**) based on KEGG and Reactome annotations. Enriched terms were determined by an FDR < 0.05 (Fisher's exact test with Benjamini–Hochberg correction). Only non-redundant terms are presented, except where additional terms provided complementary information. The fraction of proteins in each module with the corresponding annotation is provided. (**B**) Circular diagram highlighting the observed interaction between upregulated TFs (bottom) and their targets (top) within the top 30% nodes of the PPI networks based on the current-flow betweenness centrality for the three cell morphologies analyzed individually. Tracks mark the grade groups in which they were found to be significantly dysregulated (corresponds to the color-code in (**A**)) and the bands color-code for the associated signaling pathways.

© The Author(s)

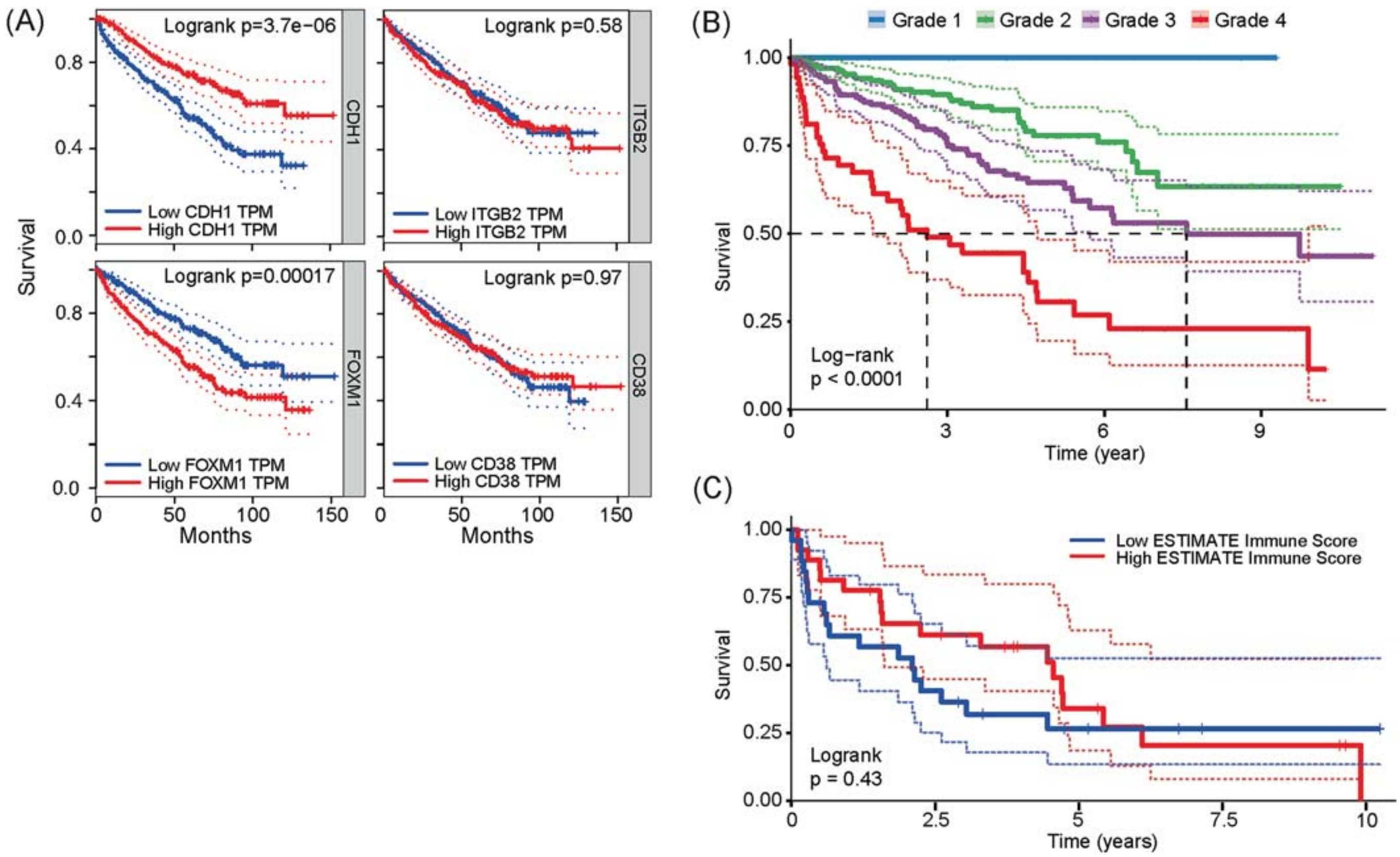

Figure EV5. Survival associations of molecules and tumor grade in ccRCC.

(**A**) Kaplan–Meier survival analysis in the TCGA-KIRC dataset ($N = 516$) showing overall survival. FOXM1 with HR (high) = 0.66 p(HR) = 0.0079. CDH1 with HR (high) = 0.48 p(HR) = 5.9e-06. CD38 with HR (high) = 1 p(HR) = 0.97. ITGB2 with HR (high) = 1.1 p(HR) = 0.58. (**B**) Kaplan–Meier survival curves stratified by tumor grade in the TCGA-KIRC cohort ($N = 434$). (**C**) Kaplan–Meier survival curves in Grade 4 ccRCC cases stratified by ESTIMATE immune score cohort ($N = 53$). HR (high) = 1 p(HR) = 0.64.

 © The Author(s)
